# *meta*-Selective olefination of fluoroarenes with alkynes using CO_2_ as a traceless directing group†

**DOI:** 10.1039/d0sc01138j

**Published:** 2020-03-31

**Authors:** Andrew R. A. Spencer, Rishi Korde, Marc Font, Igor Larrosa

**Affiliations:** School of Chemistry, University of Manchester Oxford Road Manchester M13 9PL UK igor.larrosa@manchester.ac.uk

## Abstract

Over the last few decades C–H olefination has received significant interest, due to the importance and usefulness of aryl olefins both as synthetic targets and intermediates. While a wide range of *ortho*-olefination protocols have been developed, only a small number of *meta*-olefinations are currently available. Importantly, the most common approach to *meta*-olefination, using a large *meta*-directing template, is not suitable for substrates such as fluorobenzenes, which cannot be derivatised. We report that the *meta*-selective olefination of fluoroarenes can be achieved *via* the use of CO_2_ as a traceless directing group, which can be easily installed and removed in a one-pot process. Furthermore, this approach avoids the use of stoichiometric Ag(i)-salts, commonly used in C–H olefinations, and affords complete *meta*- over *ortho*/*para*-regioselectivity.

## Abstract 2

A transient directing group approach using CO_2_ facilitates the direct *meta-*olefination of fluorobenzenes with alkynes.

## Graphical abstract

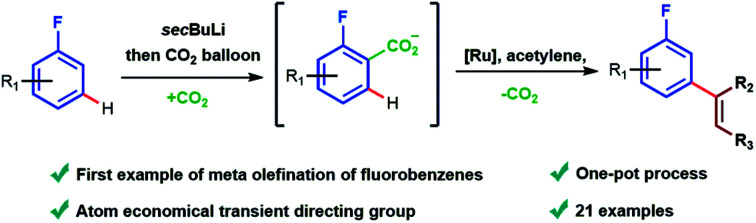

## Introduction

**Scheme 1:**
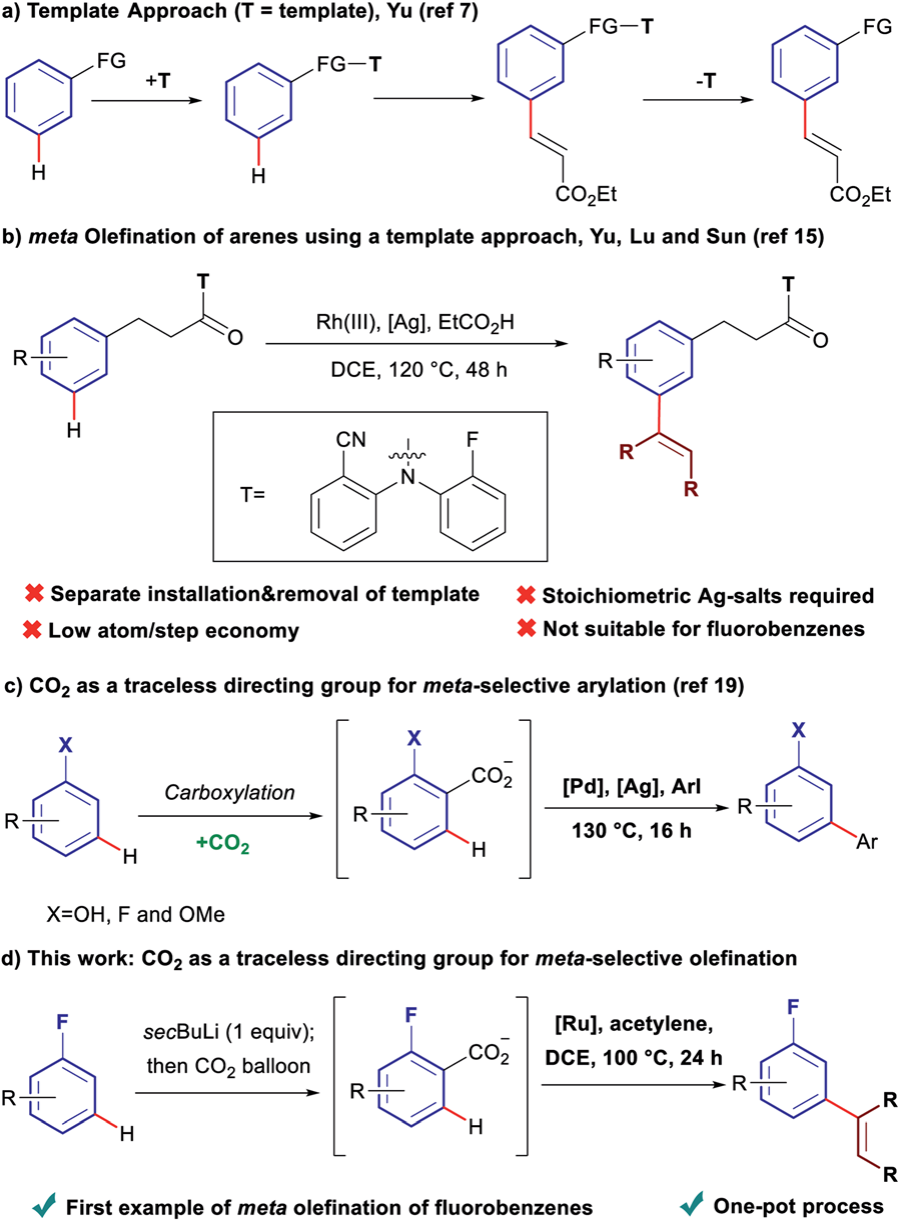
Comparison of the template approach and the traceless directing group relay strategy for the *meta* olefination of arenes.

Over the last decades, direct C–H bond activation has emerged as a powerful tool providing a wide variety of novel disconnections simplifying access to, and accelerating the synthesis of complex molecules.^1^ Aryl olefins are synthetically important motifs, as useful intermediates in synthesis,^2^ and also due to their widespread presence in bioactive molecules and pharmaceuticals.^3^ Consequently, large efforts have been devoted to the development of C–H olefination methodologies.^4^ While a large number of strategies have been developed for the olefination of C–H bonds *ortho* to a directing group,^5^ comparatively few exist for those in *meta* positions.^6^ To date, the only available ‘direct’ strategy for *meta*-olefination, involves the use of the U-shaped directing groups pioneered by the group of Yu (Scheme 1a).^7^ This approach has been used to perform *meta*-olefinations on derivatives of benzyl alcohols,^8^*N*-methyl anilines,^9^ phenyl acetic acids,^10^ benzyl sulfonyl esters,^11^ benzoic acids,^12^ aromatic carbonyls^13^ and aryl boronic acids,^14^ using alkenes as coupling partners. The major drawback of this approach arises from the need to install the large U-shaped directing group, covalently bound, and its subsequent removal after the C–H olefination, as separate synthetic steps. In addition, stoichiometric toxic Ag(i)-salts are required as terminal oxidants in these oxidative couplings. A recent report has expanded the applicability of this strategy to the Rh(iii)-catalysed *meta*-olefination of hydrocinnamic acid derivatives using alkynes as coupling partners (Scheme 1b).^15^ However, despite it being a redox neutral process, it still requires over three equivalents of Ag(i)-salts as an additive. Furthermore, in addition to the main *meta*-olefination product, 5–10% of the sometimes difficult to separate *ortho* and *para* olefination products are typically obtained. Additionally, the U-shaped directing group strategy is only applicable to aromatics containing a group that can be easily derivatised.

**Fig. 1:**
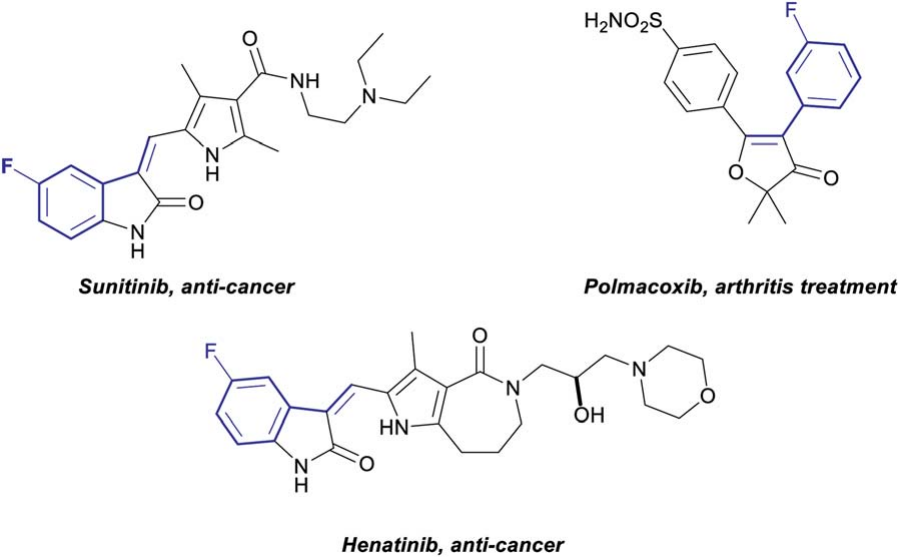
Commercially available pharmaceuticals containing a *meta* alkenyl fluoroarene.

Fluoroarenes are recurring structural motifs in pharmaceuticals, agrochemicals, organic materials and other biologically relevant compounds.^16^ Approximately 30% of pharmaceuticals and 40% of agrochemicals currently contain at least one fluorine atom, usually at the aromatic ring.^16^ Thus, the direct C–H functionalisation of monofluorobenzenes can provide straightforward access to valuable materials (Fig. 1).^3*b*–*d*^ While monofluoroarenes generally present low reactivity towards direct C–H olefination, a number of examples have been reported that use the arene as cosolvent to achieve *ortho*, *para*-selective olefination.^17^ Some pioneering methods for direct olefination using the fluoroarene as the limiting reagent have been reported by Yu *et al.*, but mixtures of *ortho, meta* and *para* substitution are obtained.^18^ However, *meta*-selective olefination has never been achieved. Importantly, the U-shaped directing group strategy cannot be applied to this class of substrates. We have previously shown that CO_2_ can be used as a traceless directing group for the *meta*-selective arylation of phenols, fluorobenzenes and anisoles (Scheme 1c).^19^ The process relies on the easy carboxylation of these aromatic substrates, to install a temporary carboxylate directing group. The carboxylate can then direct the arylation before it is cleaved, in a tandem process, thus allowing a one-pot *meta*-arylation to proceed. However, the CO_2_ traceless directing group approach has never been demonstrated on any other type of functionalisation. Herein we report the first example of a *meta*-olefination of fluorobenzenes (Scheme 1d). This ruthenium-catalysed process involves the *in situ* installation and removal of a carboxylate, from CO_2_, uses alkynes as coupling partners and avoids the need for stoichiometric use of Ag(i)-salts.

## Results and discussion

We have previously developed an optimised protocol for the lithiation/carboxylation of fluoroarenes suitable for combination in a one-pot process with a Pd-catalysed tandem arylation/protodecarboxylation, leading to the *meta-*arylation of fluoroarenes.^19*c*^ In 2016, three methods for the Ru-catalysed tandem *ortho*-olefination/protodecarboxylation of benzoic acids by hydroarylation of alkynes were reported by Hartwig and Zhao,^20^ Ackermann^21^ and Gooβen.^22^ Miura and co-workers have also reported numerous methods for the *ortho*-olefination/protodecarboxylation of benzoic acids using acrylates and styrenes as coupling partners.^23^ We envisaged these methods could be ideally adapted to operate in combination with the directed *ortho*-metalation/carboxylation approach to furnish the desired *meta*-olefination of fluorarenes.

We started our investigation by probing the decarboxylative olefination of the fluorotoluic acid **1a** with diphenyl acetylene (**2a**), using Ackermann's protocol (Table 1, entry 1).^21^ To evaluate the effect of the installation of the carboxylic acid using *ortho*-lithiation during the desired one-pot process we tested the addition of 2 equiv. of LiOAc (entry 2), revealing a significant negative effect in reactivity. Gratifyingly, addition of 3 equiv. of AcOH efficiently reversed the effect of the presence of the Li-salt (entry 3), providing a method to ensure compatibility of the protocol with the carboxylation step. In previous work on Ru-catalysed *ortho-*arylation of polyfluorobenzenes we observed an inhibitory effect of coordinated *p*-cymene, leading to the development of the arene-free Ru-precatalyst [Ru(*t*BuCN)_6_][BF_4_]_2_.^24^ However, the use of this catalyst in the olefination reaction led to no product formation (entry 4), suggesting the *η*^6^-coordinated arene is essential for reactivity towards olefination. Accordingly, the weaker coordinating benzene-complex led to poor reactivity (entry 5), whereas the highly coordinating C_6_Me_6_-bearing Ru complex gave an improved yield, an effect which has previously been observed by Gooβen.^25^

**Table 1:** Optimisation of catalyst. Yields were determined by ^19^F NMR analysis using 1-bromo-4-fluorobenzene as an internal standard

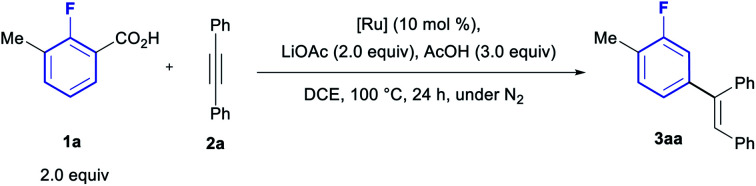		
Entry	[Ru]	**3aa** (yield%)
1^a^	Ru(*p*-cymene)(CO_2_Mes)_2_	81
2^b^	Ru(*p*-cymene)(CO_2_Mes)_2_	60
3	Ru(*p*-cymene)(CO_2_Mes)_2_	86
4	[Ru(*t*BuCN)_6_][BF_4_]_2_	0
5	[Ru(C_6_H_6_)(OPiv)_2_]	25
**6**	**[Ru(C**_**6**_ **Me**_**6**_ **)(OAc)**_**2**_ **]**	**93**

a Without LiOAc and AcOH.

b Without AcOH.

We then moved to optimize the full one-pot protocol, starting from *ortho*-fluorotoluene (**4a**, Table 2). Carboxylation of the fluoroarene was observed to occur in nearly quantitative conversion using *sec*BuLi at −78 °C for 30 min, followed by quenching with CO_2_. Subsequent addition to the same flask of AcOH (3 equiv.), alkyne **2a** and 5 mol% Ru(C_6_Me_6_)(OAc)_2_ in DCE led to the formation of the *meta*-olefinated product **3aa** in an excellent 85% yield (entry 1). The use of 4 equiv. or 5 equiv. of AcOH led to reduced yields (entries 2 and 3). Examination of other organic acids also led to lower yields (entries 4–6), revealing AcOH as the optimal acid to facilitate this one-pot process.

**Table 2:** Optimisation of one-pot protocol. Yields were determined by ^19^F NMR analysis using 1-bromo-4-fluorobenzene as an internal standard

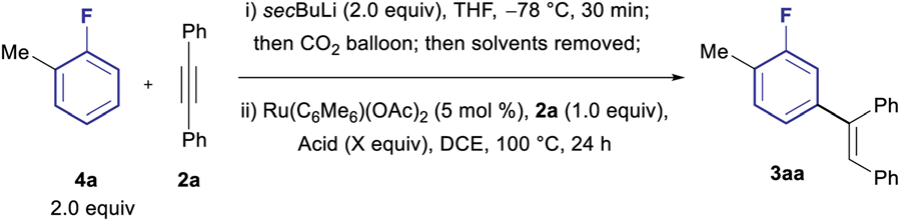		
Entry	Acid (equiv.)	**4aa** (yield%)
**1**	**AcOH (3)**	**85**
2	AcOH (4)	75
3	AcOH (5)	72
4	PivOH (3)	1
5	*i*BuCOOH (3)	71
6	TFA (3)	5

**Scheme 2:**
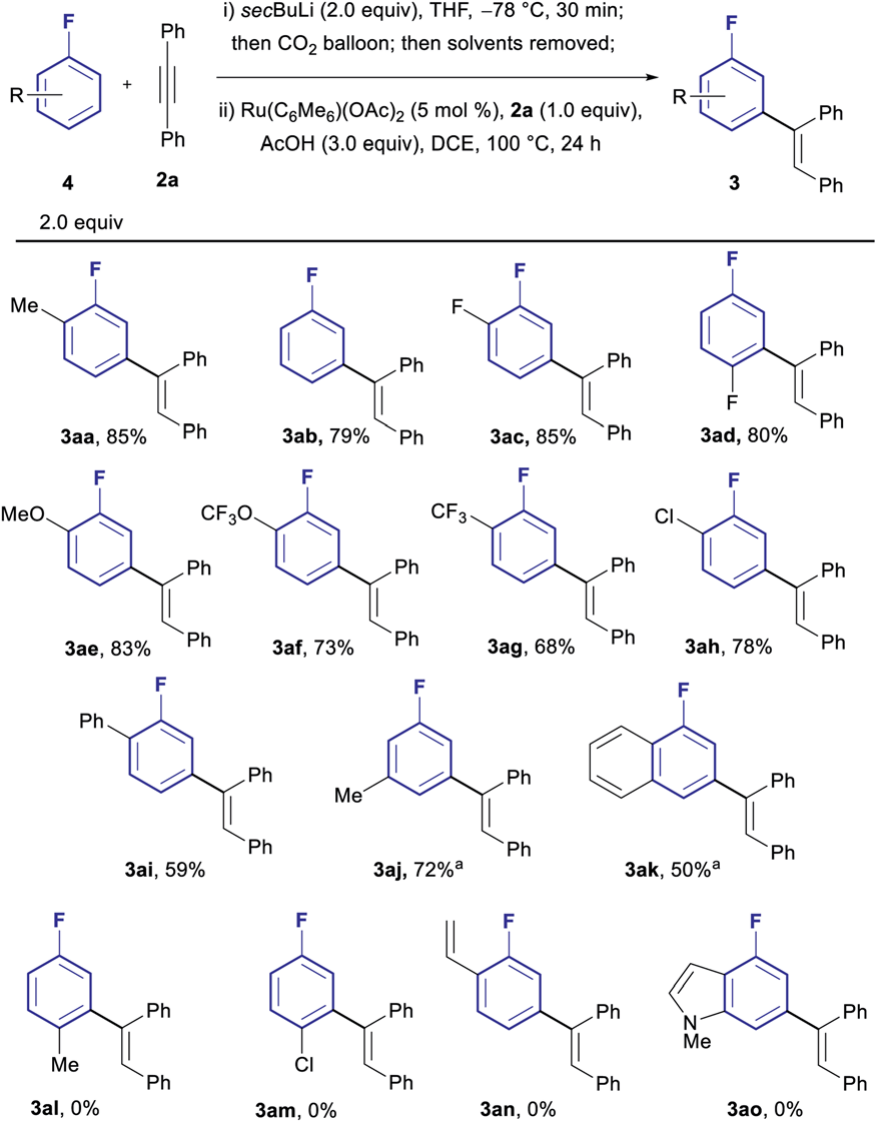
Scope in fluoroarene core. ^*a*^10 mol% catalyst used.

With the optimised conditions in hand, we investigated the generality of the process with regards to the fluoroarene core (Scheme 2). Substitution patterns in *ortho*, *meta* or *para* positions were all tolerated, albeit only the relatively small F-atom was compatible in *para* (**3ad**). When larger groups were installed in the *para* position such as Me and Cl, no reactivity could be observed (**3al** and **3am**). Furthermore, the reaction is in all cases completely selective towards mono-olefination and towards the *meta* position, with no traces of neither bisolefination nor other regioisomers observed by NMR and GCMS analysis of the reactions, even for simple fluorobenzene (**3ab**). Both electron withdrawing groups (**3ac**, **3ad**, **3ag** and **3ah**) and donating groups (**3aa**, **3ae**, **3af** and **3aj**) were compatible with the procedure. Chloroarenes (**3ah**) were also tolerated with no traces of de-halogenated products. Biaryl and naphthyl-based aromatic systems were also suitable substrates (**3ai** and **3ak**).

**Scheme 3:**
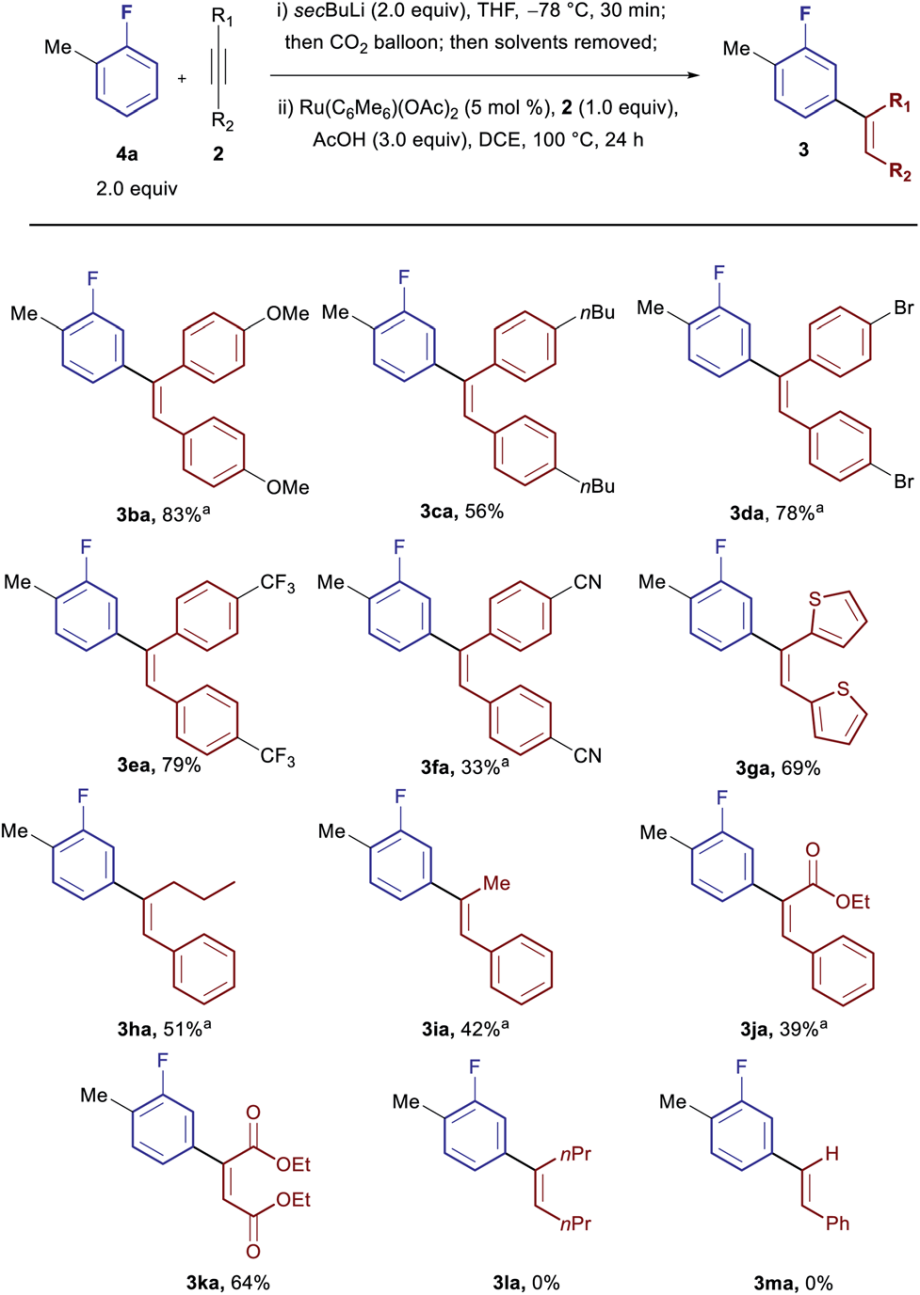
Scope in acetylene. ^*a*^10 mol% catalyst used.

Subsequently we investigated the scope with respect to the alkyne coupling partner (Scheme 3). Both electron donating (**3ba** and **3ca**) and electron withdrawing groups (**3da**, **3ea** and **3fa**) were reactive giving excellent yields. Heterocyclic moieties were also tolerated (**3ga**). While bisalkyl acetylenes were incompatible with the procedure (**3la**), unsymmetrical alkyl, aryl-acetylenes led to completely regioselective addition at the carbon adjacent to the alkyl group (**3ha** and **3ia**). Diesters and unsymmetrical ester, aryl-acetylenes were also tolerated offering a handle for further functionalisation (**3ja** and **3ka**) with ethyl phenyl propiolate preferentially forming the α-aryl ester (**3ja**). No product was observed when terminal acetylenes were used (**3ma**).

This new *meta*-olefination methodology can be easily scaled up with, for example, **3aa** being formed in 70% yield (1.10 g) without any changes to the protocol.

**Scheme 4:**
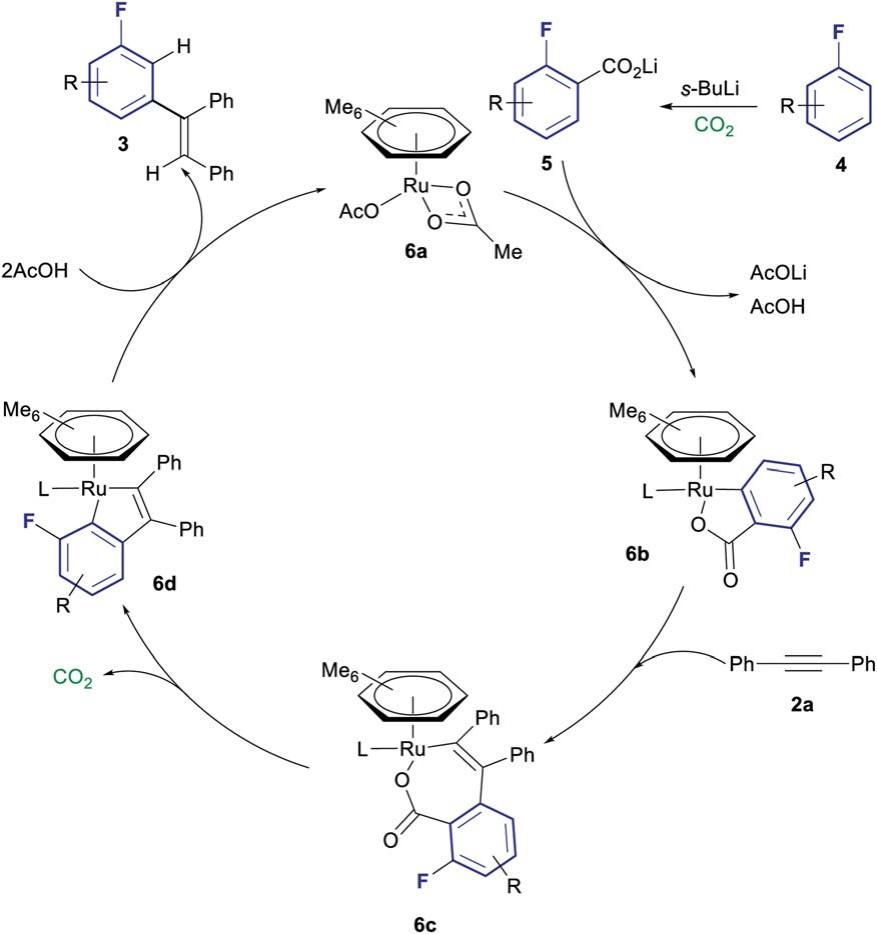
Plausible mechanism for the Ru catalysed *meta* olefination of fluoroarenes.

A plausible mechanism for this transformation is shown in Scheme 4, based on the mechanistic studies performed by Hartwig and Zhao.^20^*ortho*-Lithiation and carboxylation of fluoroarene **4** affords lithium benzoate **5**. **o*rtho*-C–H activation of lithium benzoate **5** with ruthenium complex **6a** affords cyclometallated complex **6b**. Insertion of alkyne **2a** into the Ru–C of **6b** forms complex **6c**, which can in turn decarboxylate to from the 5-membered metallocycle in complex **6d**. Protonation of this complex with 2 equiv. of AcOH liberates the final product **3** and reforms complex **6a**, thus closing the catalytic cycle.

## Conclusions

In conclusion, we have developed the first example of a methodology for the *meta*-selective olefination of fluoroarenes. The natural *ortho*, *para*-reactivity of this class of substrates has been overcome by employing CO_2_ as a traceless directing group, that can be installed, used to control reactivity and then seamlessly removed in a one-pot process. Good to excellent yields can be obtained with a variety of functional groups and substitution patterns in both fluoroarene and alkyne, and in all cases complete *meta*-regioselectivity is observed.

## Conflicts of interest

There are no conflicts to declare.

## Supplementary Material

SC-011-D0SC01138J-s001
