# Supplementary material for: meta-Selective olefination of fluoroarenes with alkynes using CO2 as a traceless directing group
Source: Chem Sci. 2020 Mar 31;11(16):4204–8. doi: 10.1039/d0sc01138j (PMC8152615; doi:10.1039/d0sc01138j)

Electronic Supplementary Material (ESI) for Chemical Science.
This journal is © The Royal Society of Chemistry 2020

Supporting Information

meta- Selective olefination of fluoroarenes with alkynes using CO_2 as a traceless directing group

Andrew R. A. Spencer, Rishi. K. Korde, Marc Font and Igor Larrosa*

School of Chemistry, University of Manchester, Oxford Road, M13 9PL, Manchester (United Kingdom)

Email: igor.larrosa@manchester.ac.uk

Table of Contents

1.1 General experimental information S2

1.2 Experimental procedures and characterisation data for *m*-olefinated fluoroarenes S2

1.3 References S14

1.4 NMR spectral data S14

1.1 General experimental information

THF was freshly distilled over sodium and benzophenone prior to its use. Commercial solutions of *sec*-butyl lithium were titrated using N-benzylbenzamide as indicator. All other solvents and reagents were purchased from commercial suppliers and used without further purification unless otherwise stated. $Ru(C_6Me_6)(OAc)_2$ was prepared according to the procedure described by Stephenson and Bennet.[1] Alkynes **2b**, **2e**, **2f** and **2g** was prepared according to the method described by Wacharasindhu.[2] All air and moisture-sensitive reactions and lithiation reactions were carried out under dry argon or nitrogen atmosphere.

Column chromatography was performed on silica gel (40-63 μm) unless otherwise stated. Thin layer chromatography (TLC) was carried out on pre-coated silica gel F_{254} plates with visualisation under UV light or using an aqueous basic $KMnO_4$ solution.

Melting points (mp) are uncorrected and were obtained using a Stuart SMP11 apparatus. IR spectra were recorded using a Thermo Scientific Nicolet iS5 FTIR spectrometer and the relevant peaks are quoted in cm^{-1}. NMR data was collected on a Bruker Avance III 400 MHz or Bruker AvanceII+ 500 MHz spectrometers. Chemical shifts are given in ppm (δ) and are referenced to the residual $CDCl_3$ solvent peak at 7.26 ppm (^1H NMR) and 77.16 ppm (^{13}C NMR). Conventional one-dimensional (1D) ^1H NMR, ^{19}F NMR, were recorded at room temperature under routine conditions. High Resolution Mass Spectra (HRMS) were performed by the School of Chemistry Mass Spectrometry Service of the University of Manchester on a Thermo Finnigan MAT95XP spectrometer. All E/Z ratios determined by ^{19}F NMR spectroscopy.

1.2 Experimental procedures and characterisation data for *meta*-olefinated fluoroarenes

General experimental procedure

A flame-dried crimpable glass schlenk vial (CEM Microwave Technologies, 10 mL volume) was loaded with the fluoroarene (if solid), capped with a rubber septum, evacuated and filled with nitrogen three times. The vial was loaded with 2.5 mL of a 0.4 M solution of fluoroarene in dry THF if liquid or 2.5 mL of dry THF if solid. The solution was cooled down to −78 °C and 0.72 mL of *sec*-butyl lithium (1.4 M in cyclohexane) were added dropwise (careful: avoid the sides of the reaction vessel to prevent the *sec*-butyl lithium freezing). After 30 min at –78 °C, CO_2 was bubbled into the reaction mixture for 10 seconds The resulting mixture was warmed up to room temperature and the solvents were removed under a stream of nitrogen. To the resulting white solid, $Ru(C_6Me_6)(OAc)_2$ (9.5 mg, 0.025 mmol), acetylene (0.5 mmol), acetic acid (86 μL, 1.5 mmol) and dichloroethane (2 mL) were added under positive pressure of nitrogen. The vial was capped with a crimpable cap septum under a positive pressure of nitrogen and the suspension was heated at 100 °C for 24 h. The reaction was quenched with

1 mL of HCl (3 M in CPME), filtered through a short plug of silica gel and concentrated *in vacuo.* Purification by column chromatography loading the crude product absorbed on silica and using the eluents specified afforded the pure *meta*-olefinated products.

Experimental procedure for 5mmol scale reaction

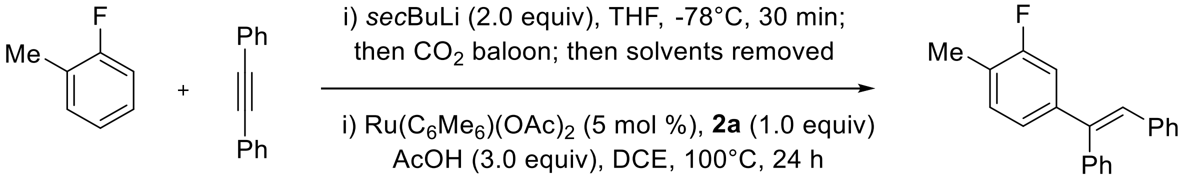

4a **2a** **3aa**

A 100 mL Ace pressure tube was capped with a rubber septum, flame dried with a blow torch, evacuated and filled with nitrogen three times. The tube was loaded with 2-fluorotoluene **4a** (1.10 mL, 10.0 mmol) and 25 mL of dry THF. The solution was cooled down to –78 °C and 7.2 mL of *sec*-butyl lithium (1.4 M in cyclohexane) were added dropwise (careful: avoid the sides of the reaction vessel to prevent the *sec*-butyl lithium freezing). After 30 min at –78 °C, CO_2 was bubbled into the reaction mixture for 20 s. The resulting mixture was warmed up to room temperature and the solvents were removed under a stream of nitrogen, and under high vacuum overnight. To the resulting white solid, $Ru(C_6Me_6)(OAc)_2$ (95.0 mg, 0.25 mmol), diphenylacetylene **2a** (891 mg, 5.0 mmol), acetic acid (0.86 mL, 15.0 mmol) and dichloroethane (20 mL) were added under a funnel of nitrogen. The vial was sealed with a screw cap and the suspension was heated at 100 °C for 24 h. The reaction was quenched with 10 mL of HCl (3 M in CPME), filtered through a short plug of silica gel and concentrated *in vacuo.* The crude product was purified by column chromatography (hexanes) to afford 1-(3-fluoro-4-methylphenyl)ethene-1,2-diyl)dibenzene (**3aa**) as a white solid (1.01 g, 70%, E/Z ratio 99:1).

1-(3-fluoro-4-methylphenyl)ethene-1,2-diyl)dibenzene

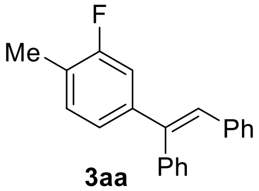

3aa

Following the general procedure, 2-fluorotoluene **4a** (110.0 µL, 1.00 mmol) was reacted with diphenyl acetylene **2a** (89.1 mg, 0.50 mmol). The crude product was purified by column chromatography (hexanes) to afford 1-(3-fluoro-4-methylphenyl)ethene-1,2-diyl)dibenzene (**3aa**) as a colourless solid (122.0 mg, 85%, E/Z ratio 99:1). **m.p.** = 70–73 °C. **^{1}H NMR** (400 MHz, $CDCl_3$) δ, ppm: 7.38 (dd, *J* = 5.0, 1.9 Hz, 3H), 7.25 (dd, *J* = 5.9, 2.4 Hz, 2H), 7.20–7.11 (m, 4H), 7.09–7.01 (m, 5H), 2.32 (d, *J* = 1.9 Hz, 3H). **^{13}C NMR** (101 MHz, $CDCl_3$) δ, ppm: 161.2 (d, *J* = 244.1 Hz), 143.1 (d, *J* = 7.4 Hz), 141.5 (d,

J = 2.2 Hz), 140.0, 137.1, 131.1 (d, J = 5.6 Hz), 130.3, 129.6, 128.8, 128.2, 128.0, 127.6, 126.9, 124.0 (d, J = 17.4 Hz), 122.9 (d, J = 3.0 Hz), 114.0 (d, J = 23.1 Hz), 14.4 (d, J = 3.3 Hz). **^{19}F NMR** (376 MHz, $CDCl_3$) δ, ppm: −117.7 (ddd, J = 10.6, 8.0, 2.1Hz). **HRMS** (ESI **$[MH]^+$**) *m/z* calculated for $C_{21}H_{18}F^+$, 289.1387, found: 289.1384. **IR** (ATR), ν, cm^{-1}: 865, 797, 775, 715, 695, 659, 545, 444.

1-(3-fluorophenyl)ethene-1,2-diyl)dibenzene

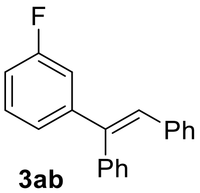

3ab

Following the general procedure, fluorobenzene **4b** (94.0 µL, 1.00 mmol) was reacted with diphenyl acetylene **2a** (89.1 mg, 0.50 mmol). The crude product was purified by column chromatography on silica (hexanes) to afford 1-(3-fluorophenyl)ethene-1,2-diyl)dibenzene (**3ab**) as a colourless solid (108.8 mg, 79%, E/Z ratio 99:1). NMR data matched the previously reported data.[3] **^{1}H NMR** (400 MHz, $CDCl_3$) δ, ppm: 7.36 (dd, J = 4.9, 1.9 Hz, 3H), 7.28 (td, J = 8.0, 6.1 Hz, 1H), 7.22 (dd, J = 6.6, 3.0 Hz, 2H), 7.18–7.11 (m, 4H), 7.08–6.95 (m, 5H). **^{13}C NMR** (101 MHz, $CDCl_3$) δ, ppm: 162.9 (d, J = 245.3 Hz), 145.9 (d, J = 7.4 Hz), 141.5 (d, J = 2.4 Hz), 139.9, 137.0, 130.4, 129.8, 129.6, 129.2, 128.9, 128.2, 127.8, 127.2, 123.3 (d, J = 2.8 Hz), 114.6 (d, J = 15.4 Hz), 114.4 (d, J = 14.7 Hz). **^{19}F NMR** (376 MHz, $CDCl_3$) δ, ppm: −113.5 (ddd, J = 10.5, 8.3, 6.0 Hz).

1-(3,4-difluorophenyl)ethene-1,2-diyl)dibenzene

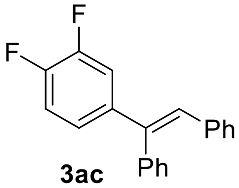

3ac

Following the general procedure, 1,2-difluorobenzene **4c** (99.0 µL, 0.50 mmol) was reacted with diphenyl acetylene **2a** (89.1 mg, 0.50 mmol). The crude product was purified by column chromatography (hexanes) to afford 1-(3,4-difluorophenyl)ethene-1,2-diyl)dibenzene (**3ac**) as a colourless oil (124.0 mg, 85%, E/Z ratio 98:2). **^{1}H NMR** (400 MHz, $CDCl_3$) δ, ppm: 7.36 (t, J = 3.2 Hz, 3H), 7.22–7.17 (m, 2H), 7.17–7.08 (m, 5H), 7.08–7.01 (m, 3H), 6.93 (s, 1H). **^{13}C NMR** (101 MHz, $CDCl_3$) δ, ppm: 151.2 (dd, J = 247.5, 12.8 Hz), 148.7 (dd, J = 248.5, 13.0 Hz), 140.6, 140.5, 139.6, 136.8, 130.3, 129.6, 128.9, 128.1, 127.8, 127.1, 123.6 (d, J = 3.3 Hz), 123.5 (d, J = 3.3 Hz), 116.9 (d, J = 17.1 Hz), 116.4 (d, J = 17.8 Hz). **^{19}F-NMR** (376 MHz, $CDCl_3$) δ, ppm: −19.3 (d, J = 17.6 Hz), −120.0 (d, J = 17.6 Hz). **HRMS** (**$[M]^+$**) *m/z* calculated for $C_{20}H_{14}F_2^+$, 292.1058, found: 292.1061. **IR** (ATR), ν, cm^{-1}: 1445, 1222, 864, 775, 739, 718, 693, 650, 632, 532.

1-(2,5-difluorophenyl)ethene-1,2-diyl)dibenzene

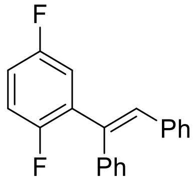

3ad

Following the general procedure, 1,4-difluorobenzene **4d** (103.0 μL, 1.00 mmol) was reacted with diphenyl acetylene **2a** (89.1 mg, 0.50 mmol). The crude product was purified by column chromatography (hexanes) to afford 1-(2,5-difluorophenyl)ethene-1,2-diyl)dibenzene (**3ad**) pale pink solid (116.8 mg, 80%, E/Z ratio 99:1). NMR data matched the previously reported data.[4] **^{1}H NMR** (400 MHz, $CDCl_3$) δ, ppm: 7.34–7.29 (m, 3H), 7.22 (dd, *J* = 6.7, 3.0 Hz, 2H), 7.18 (dd, *J* = 5.3, 1.9 Hz, 3H), 7.09 (dd, *J* = 6.9, 2.9 Hz, 2H), 7.03 (dt, *J* = 9.3, 4.7 Hz, 1H), 6.99–6.91 (m, 3H).**^{13}C NMR** (101 MHz, $CDCl_3$) δ, ppm: 158.6 (dd, *J* = 209.3, 2.0 Hz), 156.2 (dd, *J* = 212.3, 2.0 Hz), 139.6, 136.6, 136.1, 132.9 (dd, *J* = 14.9, 7.6 Hz), 132.5 (d, *J* = 4.4 Hz), 129.8, 129.6, 128.7, 128.1, 127.7, 127.3, 117.5 (dd, *J* = 24.2, 3.6 Hz), 117.0 (dd, *J* = 25.9, 8.8 Hz), 115.2 (dd, *J* = 24.0, 8.7 Hz).**^{19}F NMR** (376 MHz, $CDCl_3$) δ, ppm: −119.3 (d, *J* = 17.7 Hz), −120.0 (d, *J* = 17.6 Hz).

(1-(3-fluoro-4-methoxyphenyl)ethene-1,2-diyl)dibenzene

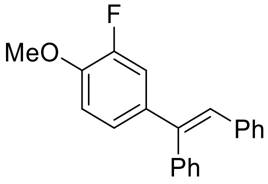

3ae

Following the general procedure, 2-fluoroanisole **4e** (112.2 μL, 1.00 mmol) was reacted with diphenyl acetylene **2a** (89.1 mg, 0.50 mmol). The crude product was purified by column chromatography with a gradient of 0% to 2% EtOAc:Hexanes to afford (1-(3-fluoro-4-methoxyphenyl)ethene-1,2-diyl)dibenzene (**3ae**) as an orange solid (126.3 mg, 83%, E/Z ratio 96:4). **^{1}H NMR** (500 MHz, $CDCl_3$) δ, ppm: δ 7.36–7.32 (m, 1H), 7.21–7.17 (m, 1H), 7.16–6.98 (m, 2H), 6.90 (t, *J* = 4.3 Hz, 1H), 3.90 (s, 1H). **^{13}C NMR** (126 MHz, $CDCl_3$) δ, ppm: 152.1 (d, *J* = 245.3 Hz), 147.1 (d, *J* = 11.1 Hz), 141.1 (d, *J* = 1.9 Hz), 139.9, 137.2, 136.8 (d, *J* = 6.0 Hz), 130.3, 129.5, 128.8, 128.0, 127.6, 127.4, 126.8, 123.4 (d, *J* = 3.3 Hz), 115.2 (d, *J* = 19.0 Hz), 112.9 (d, *J* = 2.2 Hz), 56.3. **^{19}F NMR** (376 MHz, $CDCl_3$) δ, ppm: −135.5 (dd, *J* = 12.8, 8.8 Hz).

1-(3-fluoro-4-(trifluoromethoxy)phenyl)ethene-1,2-diyl)dibenzene

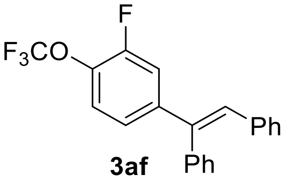

3af

Following the general procedure, 1-fluoro-2-(trifluoromethoxy)benzene **4f** (136.0 μL, 1.00 mmol) was reacted with diphenyl acetylene **2a** (89.1 mg, 0.50 mmol). The crude product was purified by column chromatography (hexanes) to afford 1-(3-fluoro-4-(trifluoromethoxy)phenyl)ethene-1,2-diyl)dibenzene (**3af**) as a colourless oil (130.1 mg, 73%, E/Z ratio 97:3). **^{1}H NMR** (400 MHz, $CDCl_3$) δ, ppm: 7.39 (m, 3H), 7.21–7.28 (m, 3H), 7.14–7.18 (m, 5H), 7.09–7.13 (m, 2H), 7.01 (s, 1H).**^{13}C NMR** (101 MHz, $CDCl_3$) δ, ppm: 154.2 (d, *J* = 252.0 Hz), 144.2 (d, *J* = 6.4 Hz), 140.3 (d, *J* = 2.0 Hz), 139.3, 136.6, 135.6 (d, *J* = 13.0 Hz), 130.2, 129.8, 129.7, 129.0, 128.1, 128.0, 127.4, 124.4 (q, *J* = 258.7 Hz) 123.4 (d, *J* = 3.4 Hz), 123.3–123.1 (m), 116.2 (d, *J* = 19.5 Hz).**^{19}F-NMR** (376 MHz, $CDCl_3$) δ, ppm: −58.7 (d, *J* = 4.7 Hz), −129.0 (q, *J* = 4.8 Hz). **HRMS** (APCI **[M]$^+$**) *m/z* calculated for $C_{21}H_{14}OF_4^+$, 358.0975, found: 358.0982. **IR** (ATR), ν, cm^{-1}: 1588, 1251, 1213, 1170, 1112, 907, 731, 717, 692, 650.

1-(3-fluoro-4-(trifluoromethyl)phenyl)ethene-1,2-diyl)dibenzene

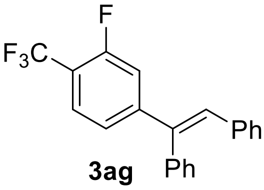

3ag

Following the general procedure, 1-fluoro-2-(trifluoromethyl)benzene **4g** (127.0 μL, 1.00 mmol) was reacted with diphenyl acetylene **2a** (89.1 mg, 0.50 mmol). The crude product was purified by column chromatography (hexanes) to afford 1-(3-fluoro-4-(trifluoromethyl)phenyl)ethene-1,2-diyl)dibenzene (**3ag**) as a colourless oil (117.1 mg, 68%, E/Z ratio 95:5). **^{1}H NMR** (400 MHz, $CDCl_3$) δ, ppm: 7.54 (t, *J* = 7.8 Hz, 1H), 7.41–7.35 (m, 3H), 7.28–7.11 (m, 7H), 7.06–7.04 (m, 3H).**^{13}C NMR** (126 MHz, $CDCl_3$) δ, ppm: 159.8 (d, *J* = 255.4 Hz), 149.8 (d, *J* = 7.9 Hz), 140.3, 139.1, 136.5, 131.0, 130.3, 129.9, 129.2, 128.9–128.4 (m), 128.3, 128.2, 127.8, 126.7-127.0 (m), 123.0 (d, *J* = 3.3 Hz), 122.8 (q, *J* = 240.6 Hz),115.8 (d, *J* = 21.3 Hz).**^{19}F-NMR** (376 MHz, $CDCl_3$) δ, ppm: −61.1 (d, *J* = 12.3 Hz), −113.2 – −116.4 (m). **HRMS** (APCI **[M]$^+$**) *m/z* calculated for $C_{21}H_{14}F_4^+$, 342.1026, found: 342.1011. **IR** (ATR), ν, cm^{-1}: 1625, 1417, 1317, 1125, 1044, 829, 755, 737, 718, 692, 536.

1-(4-chloro-3-fluorophenyl)ethene-1,2-diyl)dibenzene

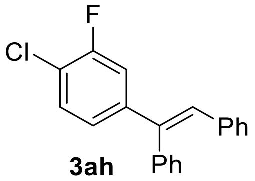

3ah

Following the general procedure, 1-Chloro-2-fluorobenzene **4h** (105.0 μL, 1.00 mmol) was reacted with diphenyl acetylene **2a** (89.1 mg, 0.50 mmol). The crude product was purified by column chromatography (petroleum ether) to afford 1-(4-chloro-3-fluorophenyl)ethene-1,2-diyl)dibenzene (**3ah**) as a colourless solid (120.4 mg, 78%, E/Z ratio 98:2). **m.p.**= 84–86 ℃. **^{1}H NMR** (400 MHz, $CDCl_3$) δ, ppm: 7.39–7.30 (m, 4H), 7.19 (dd, *J* = 6.6, 2.9 Hz, 2H), 7.15 (dd, *J* = 5.2, 1.9 Hz, 3H), 7.13–7.05 (m, 2H), 7.05–7.02 (m, 2H), 6.98 (s, 1H).**^{13}C NMR** (101 MHz, $CDCl_3$) δ, ppm: 157.9 (d, *J* = 248.0 Hz), 144.1 (d, *J* = 6.7 Hz), 140.5 (d, *J* = 2.0 Hz), 139.4, 136.7, 130.3, 130.2, 129.7, 129.4, 129.0, 128.1, 127.9, 127.3, 123.8 (d, *J* = 3.4 Hz), 119.8 (d, *J* = 17.9 Hz), 115.6 (d, *J* = 21.6 Hz).**^{19}F-NMR** (376 MHz, $CDCl_3$) δ, ppm: −115.7 (dd, *J* = 10.5, 7.7 Hz). **HRMS** (APCI **[MH]$^+$**) *m/z* calculated for $C_{20}H_{15}ClF^+$, 309.0841, found: 309.0839. **IR** (ATR), ν, cm^{-1}: 1483, 866, 733, 702, 690, 655, 550, 531.

4-(1,2-diphenylvinyl)-2-fluoro-1,1'-biphenyl

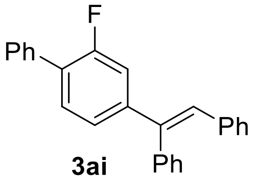

3ai

Following the general procedure, 2-fluoro-1,1'-biphenyl **4i** (172.0 mg, 1.00 mmol) was reacted with diphenyl acetylene **2a** (89.1 mg, 0.50 mmol). The crude product was purified by preparative thin layer chromatography (hexanes) to afford 4-(1,2-diphenylvinyl)-2-fluoro-1,1'-biphenyl (**3ai**) as a colourless solid (104.2 mg, 59%, E/Z ratio 96:4). **m.p.=** 115–118 °C. **^{1}H NMR** (400 MHz, $CDCl_3$) δ, ppm: 7.58 (d, *J* = 7.0 Hz, 2H), 7.46 (t, *J* = 7.5 Hz, 2H), 7.43–7.34 (m, 5H), 7.28–7.18 (m, 3H), 7.17–7.10 (m, 4H), 7.08–7.00 (m, 3H). **^{13}C NMR** (101 MHz, $CDCl_3$) δ, ppm: 159.8 (d, *J* = 247.6 Hz), 144.7 (d, *J* = 7.8 Hz), 141.2 (d, *J* = 2.2 Hz), 139.8, 137.1, 135.7, 130.4, 129.8, 129.1, 129.0, 129.0, 128.6, 128.2, 128.1, 128.0, 127.9, 127.8, 127.2, 123.5 (d, *J* = 3.0 Hz), 115.2 (d, *J* = 23.9 Hz). **^{19}F-NMR** (376 MHz, $CDCl_3$) δ, ppm: −118.3 (dd, *J* = 12.2, 8.3 Hz). **HRMS** (ESI **[MH]$^+$**) *m/z* calculated for $C_{26}H_{20}F^+$, 351.1544, found: 351.1544. **IR** (ATR), ν, cm^{-1}: 1179, 811, 768, 757, 715, 699, 689, 670, 606, 566, 457.

1-(3-fluoro-5-methylphenyl)ethene-1,2-diyl)dibenzene

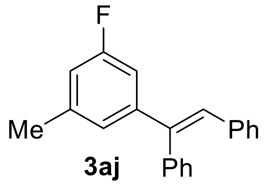

3aj

Following the general procedure, 3-fluorotoluene **4j** (111.0 µL, 1.00 mmol) was reacted with diphenyl acetylene **2a** (89.1 mg, 0.50 mmol) using 10 mol% catalyst loading. The crude product was purified by column chromatography (hexanes) to afford 1-(3-fluoro-5-methylphenyl)ethene-1,2-diyl)dibenzene (**3aj**) as a colourless oil (104.0 mg, 72%, E/Z ratio 98:2). **^{1}H NMR** (400 MHz, $CDCl_3$) δ, ppm: 7.24 (dd, *J* = 4.9, 1.9 Hz, 3H), 7.10 (dd, *J* = 6.6, 3.0 Hz, 2H), 7.07–7.00 (m, 3H), 6.92 (dd, *J* = 7.6, 2.2 Hz, 2H), 6.87 (s, 1H), 6.85 (s, 1H), 6.71 (dt, *J* = 9.6, 2.1 Hz, 2H), 2.23 (s, 3H).**^{13}C NMR** (126 MHz, $CDCl_3$) δ, ppm: 162.8 (d, *J* = 244.4 Hz), 145.5 (d, *J* = 8.2 Hz), 141.7 (d, *J* = 2.5 Hz), 140.0, 139.9, 137.1, 130.3, 129.6, 128.9, 128.8, 128.0, 127.6, 127.0, 123.9 (d, *J* = 2.4 Hz), 115.0 (d, *J* = 21.4 Hz), 111.7 (d, *J* = 22.1 Hz), 21.5 (d, *J* = 2.0 Hz).**^{19}F-NMR** (376 MHz, $CDCl_3$) δ, ppm: −114.7 (td, *J* = 9.9, 2.5 Hz). **HRMS** (ESI [**MH**]$^{+}$) *m/z* calculated for $C_{21}H_{18}F^{+}$, 289.1387, found: 289.1383. **IR** (ATR), ν, cm^{-1}: 1585, 1443, 965, 870, 845, 750, 706, 691, 654, 564, 512.

3-(1,2-diphenylvinyl)-1-fluoronaphthalene

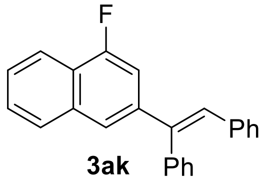

3ak

Following the general procedure, 1-fluoronapthalene **4k** (129.0 µL, 1.00 mmol) was reacted with diphenyl acetylene **2a** (89.1 mg, 0.50 mmol) using 10 mol% catalyst loading. The crude product was purified by column chromatography (2% DCM:hexanes) to afford 3-(1,2-diphenylvinyl)-1-fluoronaphthalene (**3ak**) as a colourless oil (80.7 mg, 50%, E/Z ratio 94:6). **^{1}H NMR** (500 MHz, $CDCl_3$) δ, ppm: 8.12–8.06 (m, 1H), 7.83–7.77 (m, 1H), 7.55–7.50 (m, 3H), 7.45–7.36 (m, 3H), 7.33–7.21 (m, 4H), 7.18 (d, *J* = 7.4 Hz, 2H), 7.13 (s, 1H), 7.09 (d, *J* = 7.2 Hz, 2H). **^{13}C NMR** (126 MHz, $CDCl_3$) δ, ppm: 158.8 (d, *J* = 251.2 Hz), 141.8, 141.3, 139.9, 137.2, 134.7 (d, *J* = 5.3 Hz), 130.6, 129.8, 129.3, 129.0, 128.2, 128.1 (d, *J* = 3.3 Hz), 127.9, 127.3, 127.2, 126.4, 123.3, 122.7 (d, *J* = 3.4 Hz), 120.6 (d, *J* = 4.8 Hz), 109.2 (d, *J* = 20.9 Hz).**^{19}F-NMR** (376 MHz, $CDCl_3$) δ, ppm: −123.7 (d, *J* = 12.2 Hz). **HRMS** (ESI [**MH**]$^{+}$) *m/z* calculated for $C_{24}H_{18}F^{+}$, 325.1387, found: 325.1387. **IR** (ATR), ν, cm^{-1}: 1572, 1337, 1022, 875, 791, 768, 750, 739, 704, 692, 672, 650, 541, 513.

4,4'-(1-(3-fluoro-4-methylphenyl)ethene-1,2-diyl)bis(methoxybenzene)

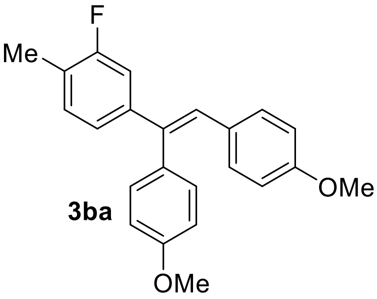

3ba

Following the general procedure, 2-fluorotoluene **4a** (110.0 μL, 1.00 mmol) was reacted with 1,2-bis(4-methoxyphenyl)ethyne **2b** (119.1 mg, 0.5 mmol) using 10 mol % catalyst. The crude product was purified by column chromatography (30% DCM:hexanes) to afford 4,4'-(1-(3-fluoro-4-methylphenyl)ethene-1,2-diyl)bis(methoxybenzene) (**3ba**) as a yellow oil (145.1 mg, 83%, E/Z ratio 92:8). **^{1}H NMR** (400 MHz, $CDCl_3$) δ, ppm: 7.11–7.06 (m, 3H), 7.02–6.92 (m, 4H), 6.91–6.82 (m, 3H), 6.68 (d, *J*=8.0 Hz, 2H), 3.85–3.82 (m, 3H), 3.76–3.73 (m, 3H), 2.26 (s, 3H). **^{13}C NMR** (126 MHz, $CDCl_3$) δ, ppm: 161.2 (d, *J* = 243.9 Hz), 158.7 (d, *J* = 56.7 Hz), 143.7 (d, *J* = 7.3 Hz), 139.1 (d, *J* = 2.3 Hz), 132.4, 131.6, 131.0 (d, *J* = 5.5 Hz), 130.8, 130.1, 127.4, 123.6 (d, *J* = 17.5 Hz), 122.7 (d, *J* = 3.0 Hz), 114.2, 113.9 (d, *J* = 23.0 Hz), 113.6, 113.5, 55.2, 55.2, 14.4 (d, *J* = 3.4 Hz).**^{19}F-NMR** (376 MHz, $CDCl_3$) δ, ppm: −117.9 – −118.2 (m). **HRMS** (APCI **[MH]$^+$**) *m/z* calculated for $C_{23}H_{22}O_2F^+$, 349.1598, found: 349.1597. **IR** (ATR), ν, cm^{-1}: 1738, 1569, 1287, 1243, 1173, 1103, 936, 866, 825, 815, 729, 528.

4,4'-(1-(3-fluoro-4-methylphenyl)ethene-1,2-diyl)bis(butylbenzene)

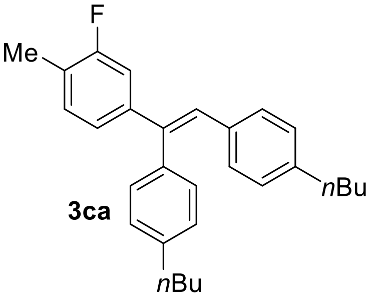

3ca

Following the general procedure, 2-fluorotoluene **4a** (110.0 μL, 1.00 mmol) was reacted 1,2-bis(4-butylphenyl)ethyne **2c** (145.6 mg, 0.5 mmol). The crude product was purified by column chromatography (petroleum ether) to afford 4,4'-(1-(3-fluoro-4-methylphenyl)ethene-1,2-diyl)bis(butylbenzene) (**3ca**) as a colourless oil (113.0 mg, 56%, E/Z ratio 97:3). **^{1}H NMR** (400 MHz, $CDCl_3$) δ, ppm: 7.15 (d, *J* = 8.1 Hz, 2H), 7.11–7.08 (m, 3H), 7.01 (dd, *J* = 7.9, 1.8 Hz, 1H), 6.99–6.88 (m, 6H), 2.65 (t, *J* = 7.9 Hz, 2H), 2.52 (t, *J* = 7.8 Hz ,2H), 2.27 (d, *J* = 1.8 Hz, 3H), 1.70–1.60 (m, 2H), 1.58–1.50 (m, 2H), 1.43–1.27 (m,4H), 0.96 (t, *J* = 7.3 Hz, 3H), 0.90 (t, *J* = 7.3 Hz, 3H). **^{13}C NMR** (101 MHz, $CDCl_3$) δ, ppm: 161.3 (d, *J* = 244.0 Hz), 143.6 (d, *J* = 7.3 Hz), 142.3, 141.8, 140.6, 137.4, 134.7, 131.1 (d, *J* = 5.4 Hz), 130.2, 129.6, 128.9, 128.2, 128.0, 123.8 (d, *J* = 17.6 Hz), 122.9 (d, *J* = 3.4 Hz),

114.1 (d, J = 22.9 Hz), 35.6, 35.5, 33.7, 33.5, 22.5, 14.5, 14.5, 14.2, 14.1. **^{19}F-NMR** (376 MHz, $CDCl_3$) δ, ppm: −118.09 (ddd, J = 11.5, 8.1, 2.0 Hz). **HRMS** (APCI **[MH]$^+$**) m/z calculated for $C_{29}H_{34}F^+$, 401.2639, found: 401.2632. **IR** (ATR), ν, cm^{-1}: 2955, 2935, 2856, 1456, 1073, 865, 817, 756, 561.

4,4'-(1-(3-fluoro-4-methylphenyl)ethene-1,2-diyl)bis(bromobenzene)

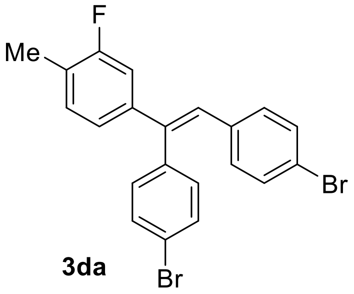

Following the general procedure, 2-fluorotoluene **4a** (110.0 μL, 1.00 mmol) was reacted with 1,2-bis(4-bromophenyl)ethyne **2d** (167.0 mg, 0.50 mmol) using 10 mol% catalyst. The crude product was purified by column chromatography (hexanes) to afford 4,4'-(1-(3-fluoro-4-methylphenyl)ethene-1,2-diyl)bis(bromobenzene) (**3da**) as an off-white solid (175.1 mg, 78%, E/Z ratio 96:4). **m.p.=** 101–106°C. **^{1}H NMR** (400 MHz, $CDCl_3$) δ, ppm: 7.47 (d, J = 8.1 Hz, 2H), 7.28 (d, J = 8.3 Hz, 2H), 7.12 (t, J = 8.0 Hz, 1H), 7.04 (d, J = 8.1 Hz, 2H), 6.93 (ddd, J = 12.6, 10.3, 1.8 Hz, 2H), 6.87 (d, J = 8.4 Hz, 3H), 2.28 (d, J = 1.8 Hz, 3H). **^{13}C NMR** (126 MHz, $CDCl_3$) δ, ppm: 161.2 (d, J = 244.5 Hz), 142.2, 141.1, 138.5, 135.7, 132.1 (d, J = 6.3 Hz), 131.3, 131.2 (d, J = 5.6 Hz), 131.0, 127.4, 124.7, 124.5, 122.9 (d, J = 3.3 Hz), 122.0, 121.0, 114.0 (d, J = 23.4 Hz), 14.4 (d, J = 3.4 Hz). **^{19}F-NMR** (376 MHz, $CDCl_3$) δ, ppm: −113.2 – −116.4 (m). **HRMS** (APCI, **[M]$^+$**) m/z calculated for $C_{21}H_{15}Br_2F^+$, 443.9519, found: 443.9502. **IR** (ATR), ν, cm^{-1}: 1484, 1065, 1009, 866, 857, 792, 749, 705, 491, 465.

4,4'-(1-(3-fluoro-4-methylphenyl)ethene-1,2-diyl)bis((trifluoromethyl)benzene)

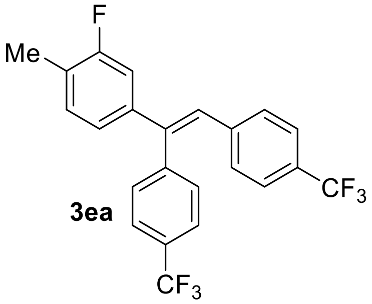

Following the general procedure, 2-fluorotoluene **4a** (110.0 μL, 1.00 mmol) was reacted 1,2-bis(4-(trifluoromethyl)phenyl)ethyne **2e** (157.0 mg, 0.5 mmol). The crude product was purified by column chromatography (hexanes) to afford 4,4'-(1-(3-fluoro-4-methylphenyl)ethene-1,2-diyl)bis((trifluoromethyl)benzene) (**3ea**) as an off-white solid (167.1 mg, 79%, E/Z ratio 99:1). **m.p.=** 97–99°C. **^{1}H NMR** (400 MHz, $CDCl_3$) δ, ppm: 7.61 (d, J = 8.0 Hz, 2H), 7.41 (d, J = 8.2 Hz, 2H), 7.30

(d, J = 7.9 Hz, 2H), 7.15 (t, J = 8.3 Hz 1H), 7.08 (d, J = 8.2 Hz, 2H), 7.02 (s, 1H), 6.99–6.89 (m, 2H), 2.29 (d, J = 1.8 Hz, 3H). **^{13}C NMR** (101 MHz, $CDCl_3$) δ, ppm: 161.4 (d, J = 245.0 Hz), 143.3, 142.6 (d, J = 2.2 Hz), 141.8, 141.6, 140.2, 131.5 (d, J = 5.6 Hz), 130.9, 130.3 (d, J = 32.6 Hz), 129.8, 129.1 (d, J = 32.4 Hz), 127.8, 126.0 (q, J = 3.8 Hz), 125.3 (td, J = 6.9, 3.0 Hz), 124.2 (q, J = 272.2 Hz), 124.2 (q, J = 272.0 Hz), 123.2 (d, J = 3.1 Hz), 114.3 (d, J = 23.4 Hz), 14.5 (d, J = 3.4 Hz). **^{19}F-NMR** (376 MHz, $CDCl_3$) δ, ppm: −62.5, −62.6, −117.1 (ddq, J = 9.9, 7.9, 2.0 Hz). **HRMS** (APCI **[M]$^+$**) *m/z* calculated for $C_{23}H_{15}F_7^+$, 424.1056, found: 424.1053. **IR** (ATR), ν, cm^{-1}: 2221, 1407, 1232, 1210, 1186, 1175, 898, 888, 851, 830, 782, 552.

4,4'-(1-(3-fluoro-4-methylphenyl)ethene-1,2-diyl)dibenzonitrile

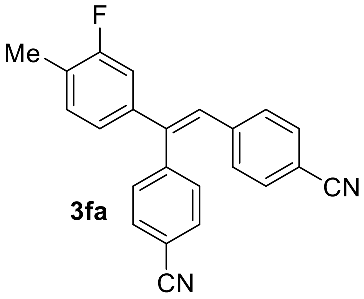

3fa

Following the general procedure, 2-fluorotoluene **4a** (55.0 µL, 0.50 mmol) was reacted 4,4'-(ethyne-1,2-diyl)dibenzonitrile **2f** (57.0 mg, 0.25 mmol). The crude product was purified by column chromatography (13% EtOAc:hexanes) to afford 4,4'-(1-(3-fluoro-4-methylphenyl)ethene-1,2-diyl)dibenzonitrile (**3fa**) as a yellow solid (28.0 mg, 33%, E/Z ratio 97:3). **m.p.=** 128–140°C. **^{1}H NMR** (400 MHz, $CDCl_3$) δ, ppm: 7.64 (d, J = 8.4 Hz, 2H), 7.44 (d, J = 8.4 Hz, 2H), 7.28 (d, J = 8.3 Hz, 2H), 7.16 (t, J = 7.8 Hz 1H), 7.06 (d, J = 8.3 Hz, 2H), 7.00 (s, 1H), 6.95–6.87 (m, 2H), 2.29 (d, J = 1.8 Hz, 3H). **^{13}C NMR** (101 MHz, $CDCl_3$) δ, ppm: 161.4 (d, J = 245.6 Hz), 144.2, 143.2, 141.0, 141.0, 132.8, 132.2, 131.7, 131.7, 131.2, 130.1, 127.8, 125.8 (d, J = 17.4 Hz), 123.3 (d, J = 3.3 Hz), 118.6 (d, J = 19.3 Hz), 114.4 (d, J = 23.4 Hz), 112.3, 110.9, 14.6 (d, J = 3.3 Hz).**^{19}F-NMR** (376 MHz, $CDCl_3$) δ, ppm: −116.7 (ddd, J = 10.1, 8.1, 2.1 Hz). **HRMS** (APCI **[M]$^+$**) *m/z* calculated for $C_{23}H_{15}F_7^+$, 424.1056, found: 424.1053. **IR** (ATR), ν, cm^{-1}: 2210, 1507, 1407, 897, 887, 852, 832, 805, 529, 423, 411.

2,2'-(1-(3-fluoro-4-methylphenyl)ethene-1,2-diyl)dithiophene

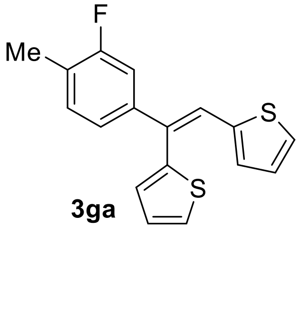

3ga

Following the general procedure, 2-fluorotoluene **4a** (110.0 μL, 1.00 mmol) was reacted 1,2-di(thiophen-2-yl)ethyne **2g** (95.0 mg, 0.50 mmol). The crude product was purified by column chromatography (hexanes) to afford 2,2'-(1-(3-fluoro-4-methylphenyl)ethene-1,2-diyl)dithiophene (**3ga**) as a green oil (103.0 mg, 69%, E/Z ratio 73:27). ^{1}**H NMR** (400 MHz, $CDCl_3$) δ, ppm: 7.54 (dd, *J* = 5.1, 1.2 Hz, 1H Major), 7.36 (s, 1H Major), 7.30 (t, *J* = 8.4Hz 1H Minor), 7.25 (s, 1H Minor), 7.21–6.98 (m, 7H Major, 4H Minor), 6.93 (m, 1H Major, 2H Minor), 6.88 (dd, *J* = 5.1, 3.6 Hz, 1H Minor), 6.65 (dd, *J* = 3.6, 1.2 Hz, 1H Minor), 2.39 (d, *J* = 1.9 Hz, 3H Minor), 2.27 (d, *J* = 1.9 Hz, 3H Major). ^{13}**C NMR** (101 MHz, $CDCl_3$) δ, ppm: 162.0 (d, *J* = 246 Hz, Minor), 161.4 (d, *J* = 245 Hz, Major), 146.9 (Minor), 141.5 (d, *J* = 7.4 Hz, Major), 140.7 (Major), 140.4 (Minor), 139.3 (Major), 137.8 (d, *J* = 7.8 Hz, Minor), 132.9 (d, *J* = 1.7 Hz, Minor), 132.5 (d, *J* = 5.5 Hz, Minor), 131.3 (d, *J* = 5.5 Hz, Major), 130.7 (d, *J* = 2.5 Hz, Major), 130.1 (Major),129.1 (Minor),129.1 (Major), 127.9 (Major), 127.9 (Major), 127.8 (Minor), 127.6 (Major), 126.7 (Minor), 126.5 (Minor), 126.4 (Major) , 126.0 (Minor), 125.6 (d, *J* = 3.4 Hz, Minor), 125.3 (d, *J* = 17.1 Hz, Minor), 124.8 (Minor), 124.5 (Major), 124.3 (d, *J* = 17.6 Hz, Major), 121.8 (d, *J* = 3.2 Hz, Major), 119.9 (Minor), 116.8 (d, *J* = 22.4 Hz, Minor), 113.1 (d, *J* = 23.8 Hz, Major), 14.8 (d, *J* = 3.5Hz, Minor), 14.5 (d, *J* = 3.2Hz, Major). ^{19}**F NMR** (376 MHz, $CDCl_3$-*d*) δ, ppm: −116.46 (tq, *J* = 8.2, 2.0 Hz, Major), −117.63 (tq, *J* = 7.3, 2.0 Hz Minor). **HRMS** (APCI **[MH]**$^+$) *m/z* calculated for $C_{17}H_{14}FS_2^+$, 301.0515, found: 301.0509. **IR** (ATR), ν, cm^{-1}: 1498, 1409, 1264, 1125, 860, 851, 818, 693, 624, 611, 579, 539, 499.

2-fluoro-1-methyl-4-(1-phenylpent-1-en-2-yl)benzene

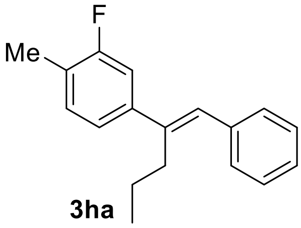

3ha

Following the general procedure, 2-fluorotoluene **4a** (110.0 μL, 1.00 mmol) was reacted with pent-1-yn-1-ylbenzene **2h** (80.0 μL, 0.50 mmol) using 10 mol% catalyst. The crude product was purified by column chromatography (hexanes) to afford 1-(3-fluoro-4-(trifluoromethyl)phenyl)ethene-1,2-diyl)dibenzene (**3ha**) as a colourless solid (65.1 mg, 51%). **m.p.=** 45–46°C. ^{1}**H NMR** (400 MHz, $CDCl_3$) δ, ppm: 7.39 (t, *J* = 7.5 Hz, 2H), 7.32 (d, *J* = 7.6 Hz, 2H), 7.27 (d, *J* = 2.3 Hz, 1H), 7.22–7.08 (m, 3H), 6.72 (s, 1H), 2.73–2.61 (m, 2H), 2.31 (d, *J* = 1.8 Hz, 3H), 1.46 (dt, *J* = 15.0, 7.5 Hz, 2H), 0.92 (t, *J* = 7.3 Hz, 3H). ^{13}**C NMR** (126 MHz, $CDCl_3$) δ, ppm: 161.3 (d, *J* = 244.0 Hz), 142.7 (d, *J* = 7.4 Hz), 142.0 (d, *J* = 2.0 Hz), 138.1, 131.2 (d, *J* = 5.8 Hz), 128.8, 128.3, 128.3, 126.6, 123.5 (d, *J* = 17.6 Hz), 121.9 (d, *J* = 3.0 Hz), 113.1 (d, *J* = 22.8 Hz), 32.0, 22.0, 14.3 (d, *J* = 3.4 Hz), 14.2. ^{19}**F-NMR** (376 MHz, $CDCl_3$) δ, ppm: −117.9 – −118.0 (m). **HRMS** (ESI **[M]**$^+$) *m/z* calculated for $C_{18}H_{19}F^+$ 254.1465, found: 254.1467. **IR** (ATR), ν, cm^{-1}: 1413, 1129, 863, 811, 743, 704, 696, 443.

2-fluoro-1-methyl-4-(1-phenylprop-1-en-2-yl)benzene

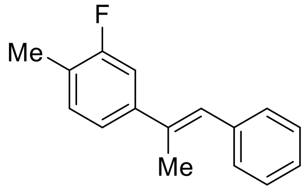

3ia

Following the general procedure, 2-fluorotoluene **4a** (110.0 μL, 1.00 mmol) was reacted with prop-1-yn-1-ylbenzene **2i** (62.6 μL, 0.50 mmol) using 10 mol % catalyst. The crude product was purified by column chromatography (0.5% DCM:hexanes) to afford 2-fluoro-1-methyl-4-(1-phenylprop-1-en-1-yl)benzene (**3ia**) as a colourless solid (47.0 mg, 42%, E/Z ratio 99:1). **m.p.=** 74–82°C. ^{1}**H NMR** (400 MHz, $CDCl_3$) δ, ppm: 7.41–7.35 (m, 4H), 7.30–7.22 (m, 1H), 7.23–7.14 (m, 3H), 6.85 (s, 1H), 2.30 (s, 3H), 2.26 (s, 3H). ^{13}**C NMR** (126 MHz, $CDCl_3$) δ, ppm: 161.4 (d, *J* = 244.0 Hz), 143.6 (d, *J* = 7.3 Hz), 138.2, 136.3 (d, *J* = 2.0 Hz), 131.3 (d, *J* = 5.7 Hz), 129.3, 128.3, 127.8, 126.7, 123.7 (d, *J* = 17.5 Hz), 121.3 (d, *J* = 3.1 Hz), 112.6 (d, *J* = 23.0 Hz), 17.5, 14.5 (d, *J* = 3.4 Hz).^{19}**F-NMR** (376 MHz, $CDCl_3$) δ, ppm: −117.9 (t, *J* = 9.7 Hz). **HRMS** (APCI **[MH]**$^+$) *m/z* calculated for $C_{16}H_{16}F^+$, 227.1231, found: 227.1221. **IR** (ATR), ν, cm^{-1}: 1561, 1445, 1269, 1128, 863, 812, 723, 699, 559.

ethyl-2-(3-fluoro-4-methylphenyl)-3-phenylacrylate

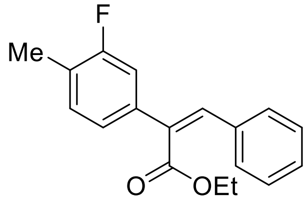

3ja

Following the general procedure, 2-fluorotoluene **4a** (110.0 μL, 1.00 mmol) was reacted ethyl 3-phenylpropiolate **2j** (82.6 μL, 0.50 mmol). The crude product was purified by preparatory thin layer chromatography (hexanes) ethyl-2-(3-fluoro-4-methylphenyl)-3-phenylacrylate (**3ja**) to afford ethyl-2-(3-fluoro-4-methylphenyl)-3-phenylacrylate as an off-white oil (55.0 mg, 39%, E/Z ratio 99:1)(19% of other isomer determined by ^{19}F NMR). ^{1}**H NMR** (400 MHz, $CDCl_3$) δ, ppm: 7.40–7.29 (m, 5H), 7.22–7.11 (m, 3H), 7.02 (s, 1H), 4.27 (q, *J* = 7.2 Hz, 2H), 2.29 (d, *J* = 1.9 Hz, 3H), 1.20 (t, *J* = 7.1 Hz, 3H). ^{13}**C NMR** (101 MHz, $CDCl_3$) δ, ppm: 169.4, 161.5 (d, *J* = 245.1 Hz), 136.6, 135.7, 134.3 (d, *J* = 2.5Hz), 131.7 (d, *J* = 5.6 Hz), 131.4, 128.6, 128.5, 128.4, 125.2 (d, *J* = 17.5 Hz), 121.9 (d, *J* = 3.2 Hz), 113.1 (d, *J* = 23.6 Hz), 61.6, 14.5 (d, *J* = 3.3 Hz), 14.0.^{19}**F NMR** (376 MHz, $CDCl_3$) δ, ppm: −116.9 – −117.1

(m). **HRMS** (APCI **[MH]**$^+$) *m/z* calculated for $C_{18}H_{18}O_2F^+$, 285.1285, found: 285.1283. **IR** (ATR), ν, cm^{-1}: 1718, 1214, 1194, 1153, 1120, 1025, 812, 752, 694.

diethyl 2-(3-fluoro-4-methylphenyl)maleate

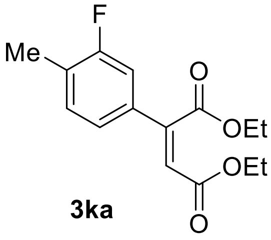

3ka

Following the general procedure, 2-fluorotoluene **4a** (110.0 µL 1.00 mmol) was reacted diethyl but-2-ynedioate **2k** (80.0 µL, 0.50 mmol). The crude product was purified by preparatory thin layer chromatography (4% EtOAc:hexanes) to afford ethyl-2-(3-fluoro-4-methylphenyl)-3-phenylacrylate (**3ka**) as a colourless oil (90.0 mg, 64%, E/Z ratio 98:2). **^{1}H NMR** (400 MHz, $CDCl_3$) δ, ppm: 7.23–7.11 (m, 3H), 6.26 (s, 1H), 4.42 (q, *J* = 7.2 Hz, 2H), 4.24 (q, *J* = 7.1 Hz, 2H), 2.29 (d, *J* = 1.9 Hz, 3H), 1.37 (t, *J* = 7.2 Hz, 3H), 1.31 (t, *J* = 7.1 Hz, 3H). **^{13}C NMR** (101 MHz, $CDCl_3$) δ, ppm: 167.7, 164.9, 161.5 (d, *J* = 246.1 Hz), 147.7 (d, *J* = 2.7 Hz), 133.0 (d, *J* = 7.9 Hz), 132.1 (d, *J* = 5.4 Hz), 127.9 (d, *J* = 17.5 Hz), 122.4 (d, *J* = 3.3 Hz), 117.7, 113.4 (d, *J* = 24.1 Hz), 62.1, 61.1, 14.7 (d, *J* = 3.4 Hz), 14.3, 14.1. **^{19}F NMR** (376 MHz, $CDCl_3$) δ, ppm: −116.0 (ddt, *J* = 10.0, 6.0, 2.1 Hz). **HRMS** (ESI **[M-Na]**$^+$) *m/z* calculated for $C_{15}H_{17}O_4FNa^+$, 303.1003, found: 303.0999. **IR** (ATR), ν, cm^{-1}: 1716, 1370, 1231, 1205, 1178, 1158, 1122, 1030, 867.

1.3 References

1. D. A. Tocher, R. O. Gould, T. A. Stephenson, M. A. Bennett, J. P. Ennett, T. W. Matheson, L. Sawyer and V. K. Shah, *J. Chem. Soc., Dalton Trans.*, 1983, 1571–1581.

2. P. Chuentragool, K. Vongnam, P. Rashatasakhon, M. Sukwattanasinitt and S. Wacharasindhu, *Tetrahedron*, 2011, **67**, 8177–8182.

3. J. Zhang, R. Shrestha, J. F. Hartwig and P. Zhao, *Nat. Chem.*, 2016, **8**, 1144–1151.

4. R. C. Jones, M. Gałęzowski and D. F. O'Shea, *J. Org. Chem.*, 2013, **78**, 8044–8053.

NMR spectral data

^{1}H NMR (400MHz, $CDCl_3$)

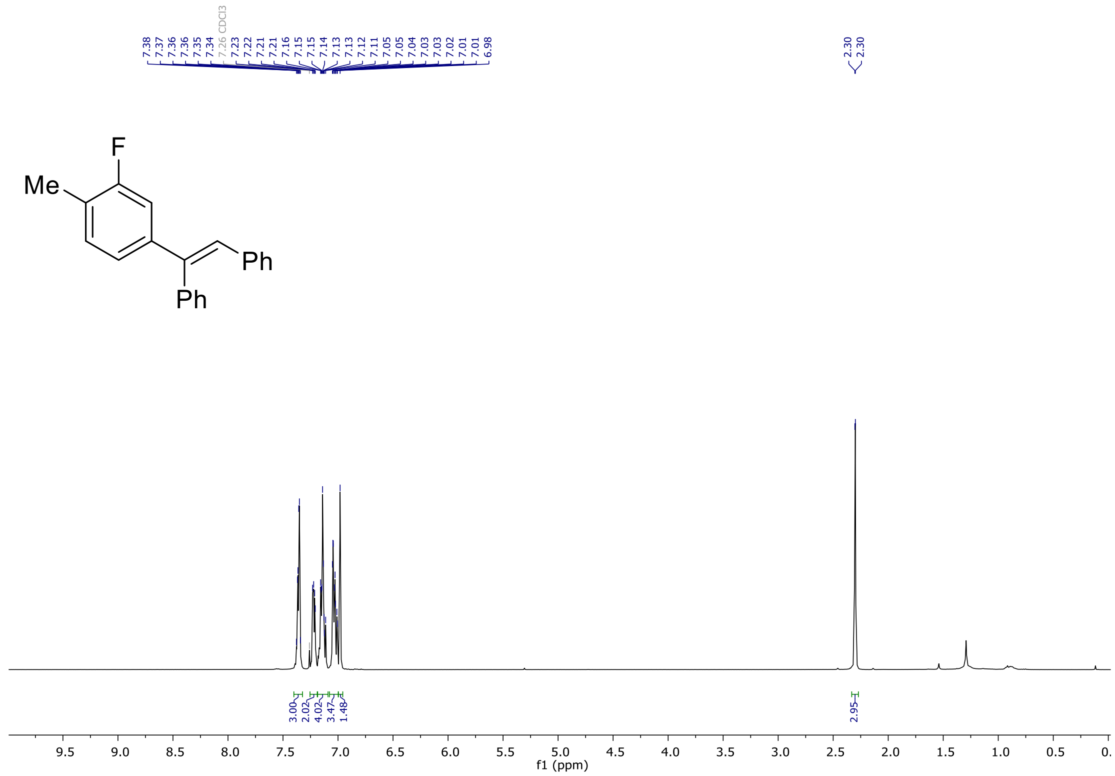

^{19}F NMR (376MHz, $CDCl_3$)

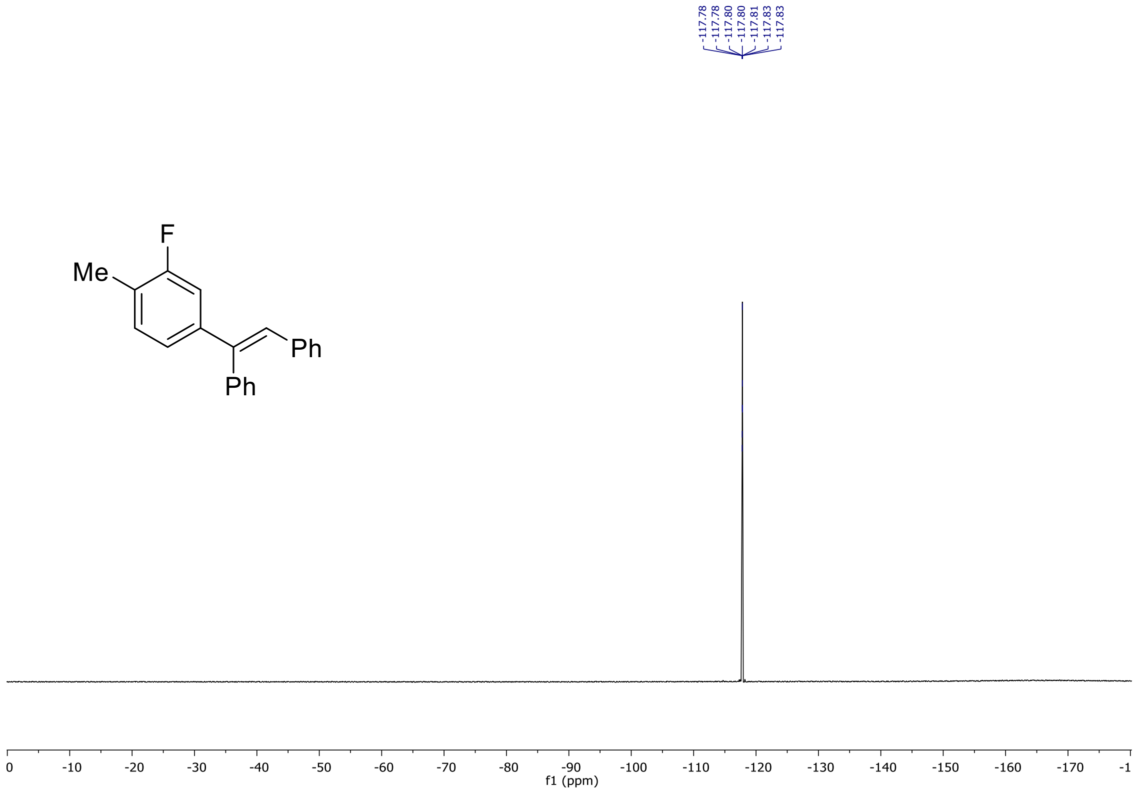

^{13}C NMR (101MHz, $CDCl_3$)

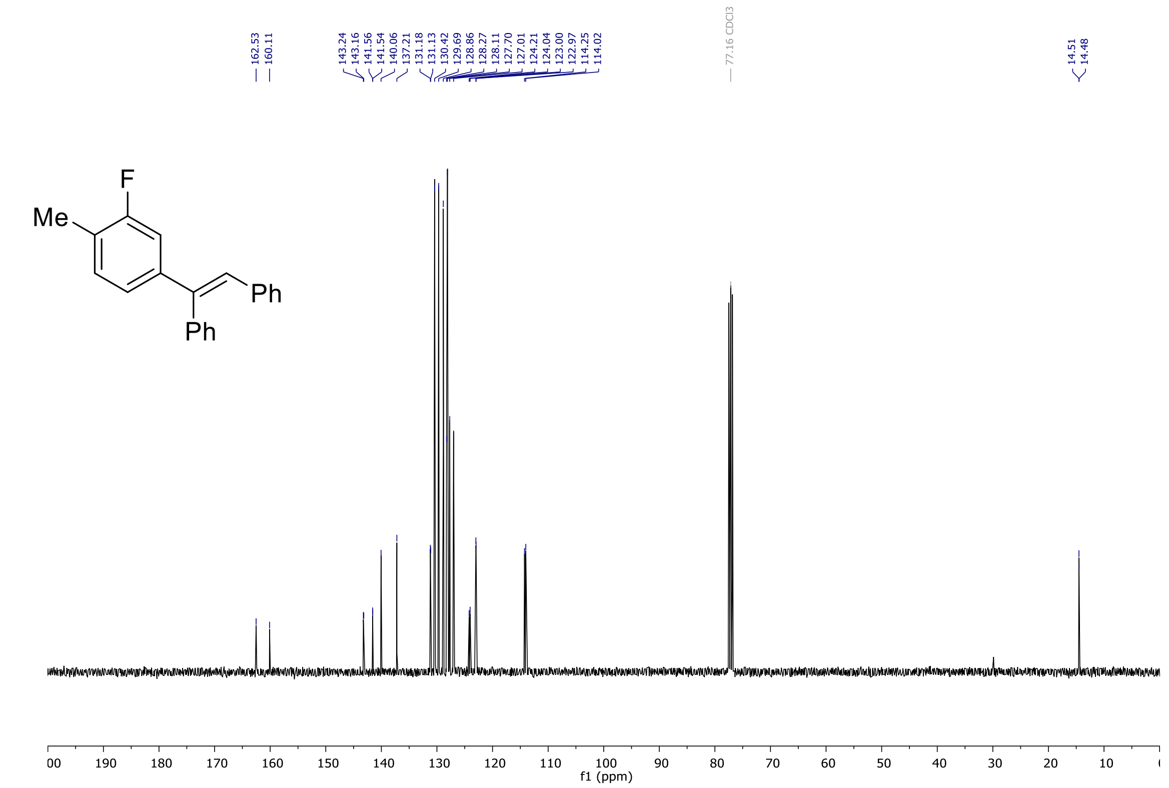

^{1}H NMR (400MHz, $CDCl_3$)

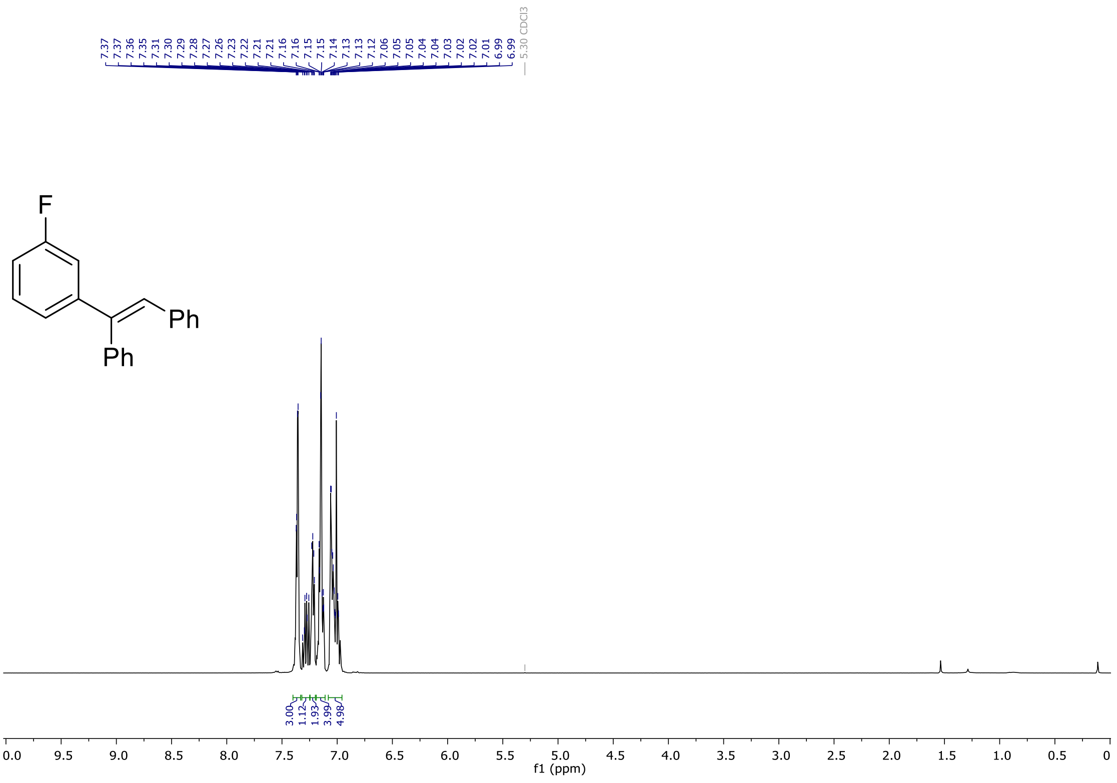

^{19}F NMR (376MHz, $CDCl_3$)

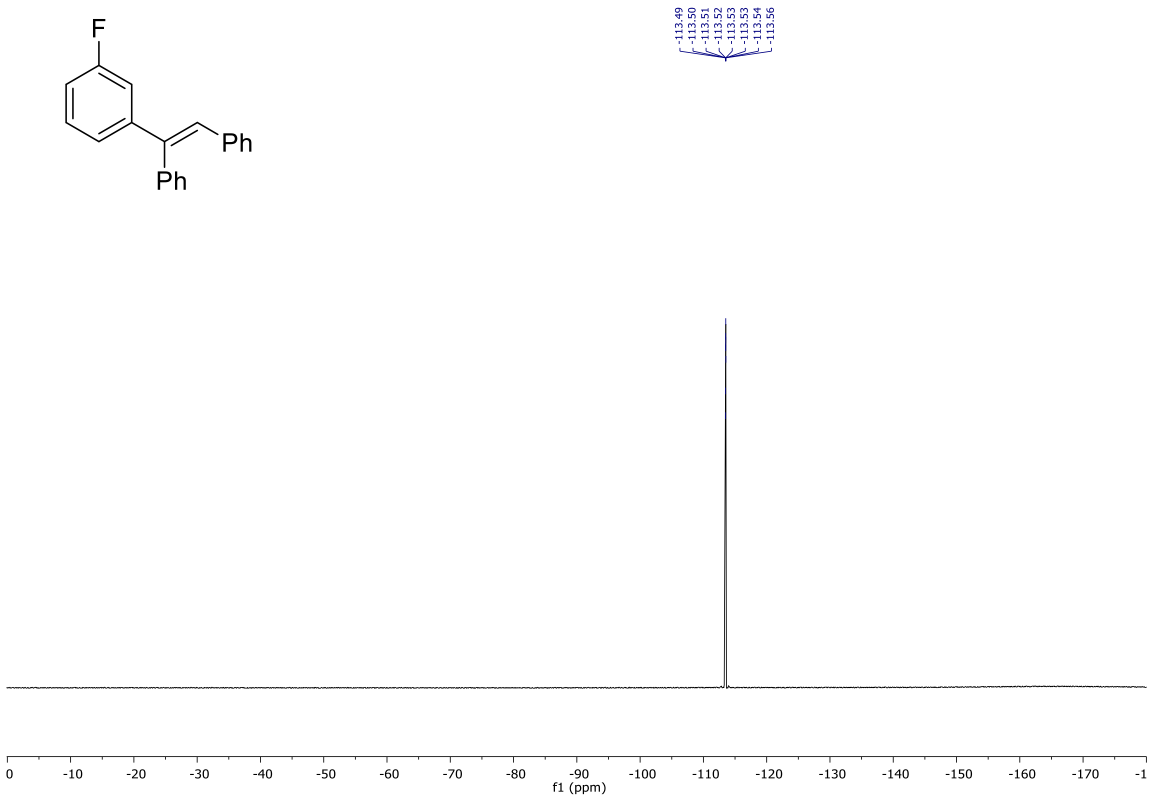

^{13}C NMR (101MHz, $CDCl_3$)

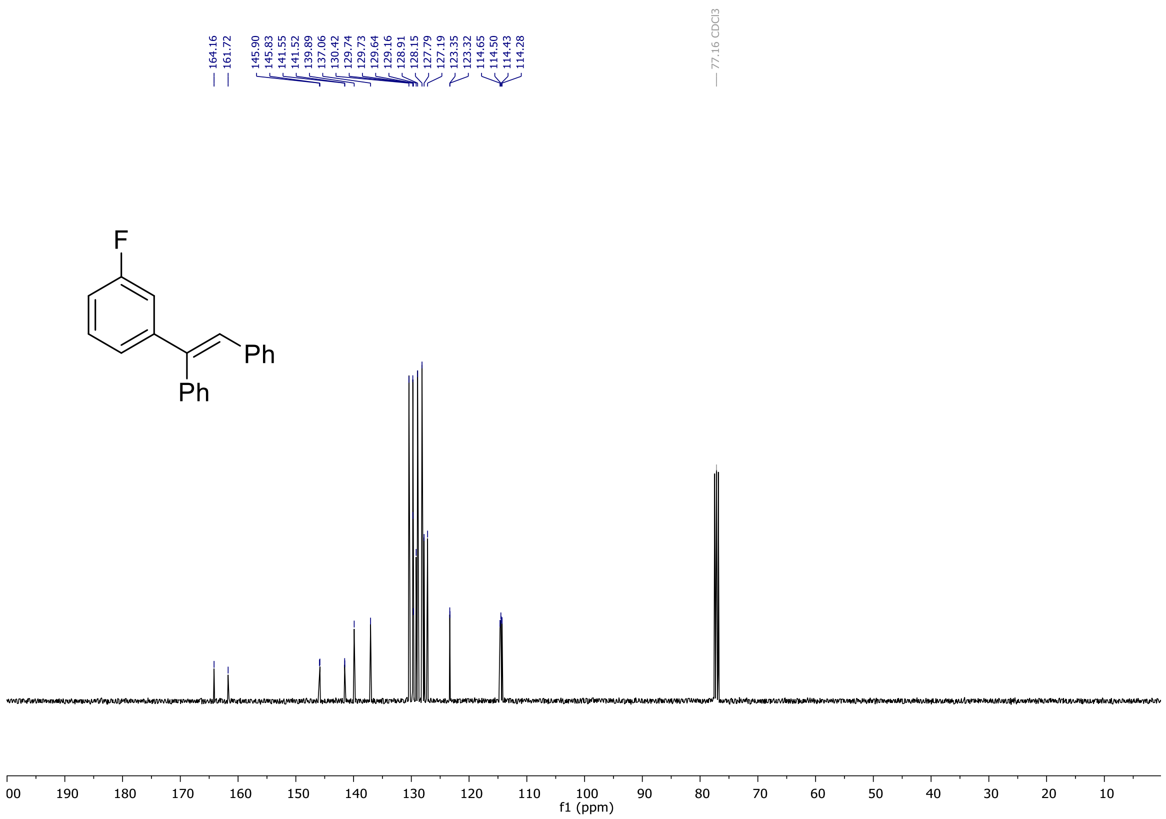

¹H NMR (400MHz, $CDCl_3$)

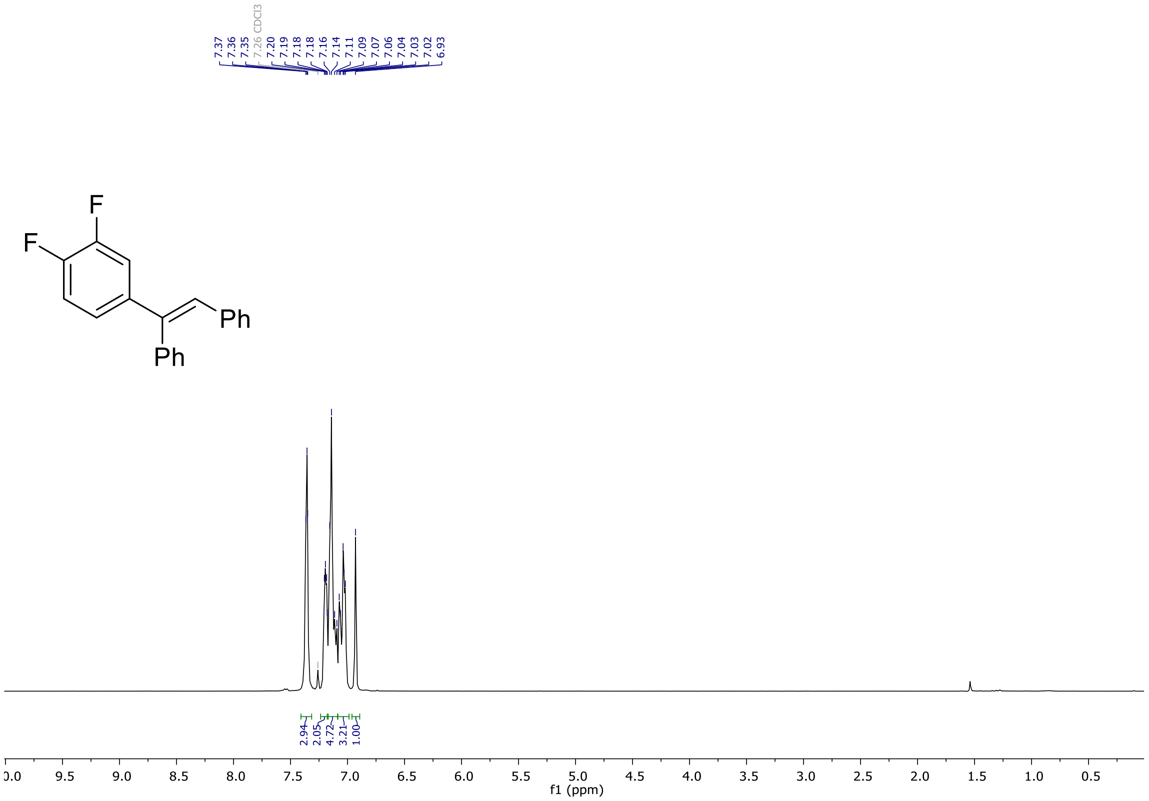

¹⁹F NMR (376MHz, $CDCl_3$)

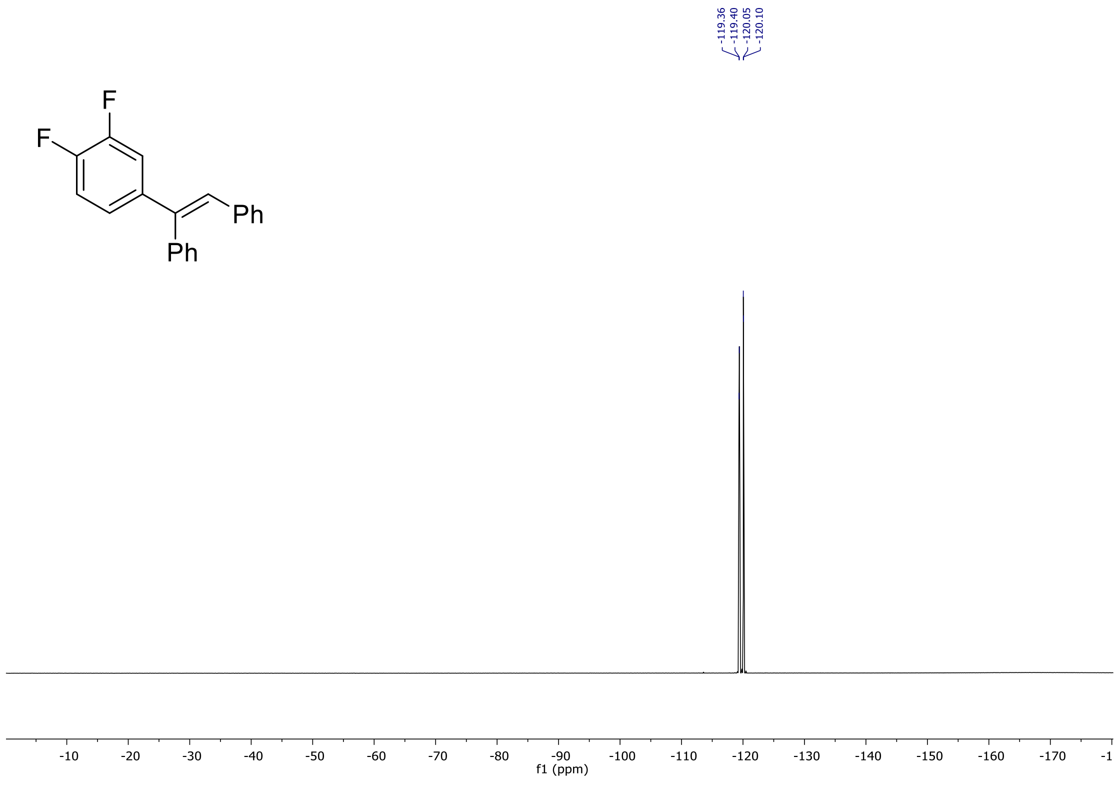

^{13}C NMR (101MHz, $CDCl_3$)

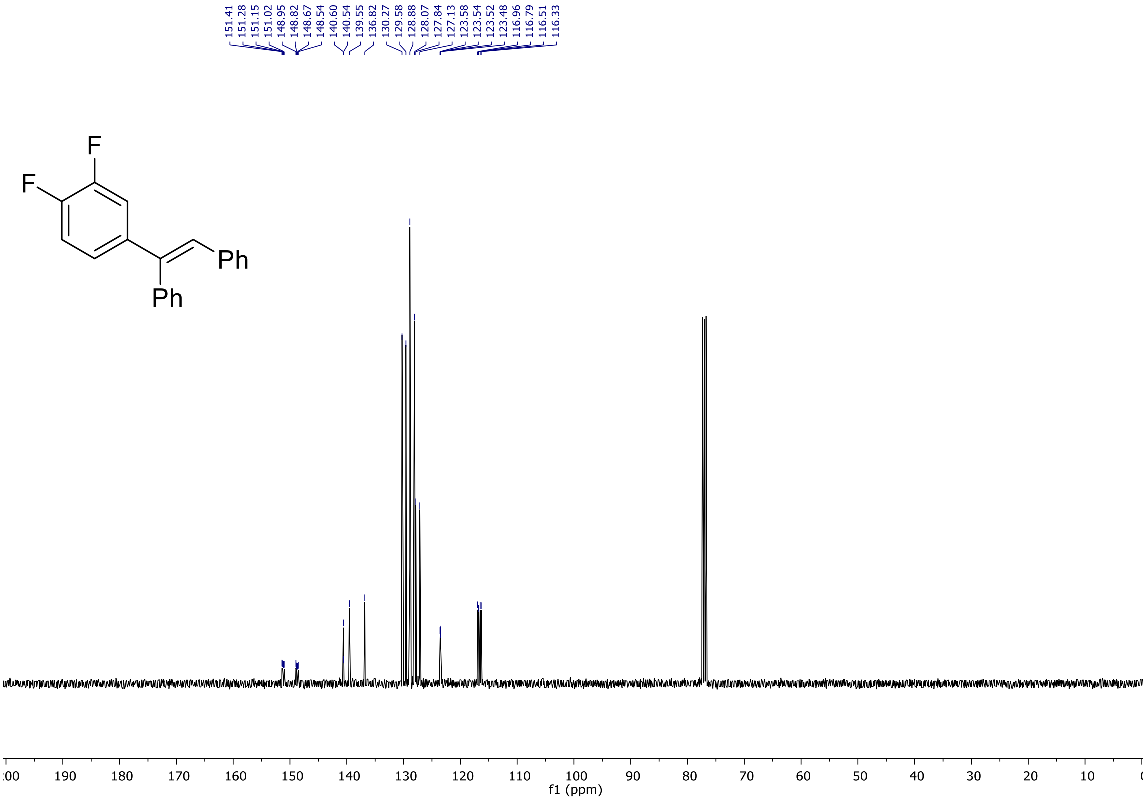

^{1}H NMR (400MHz, $CDCl_3$)

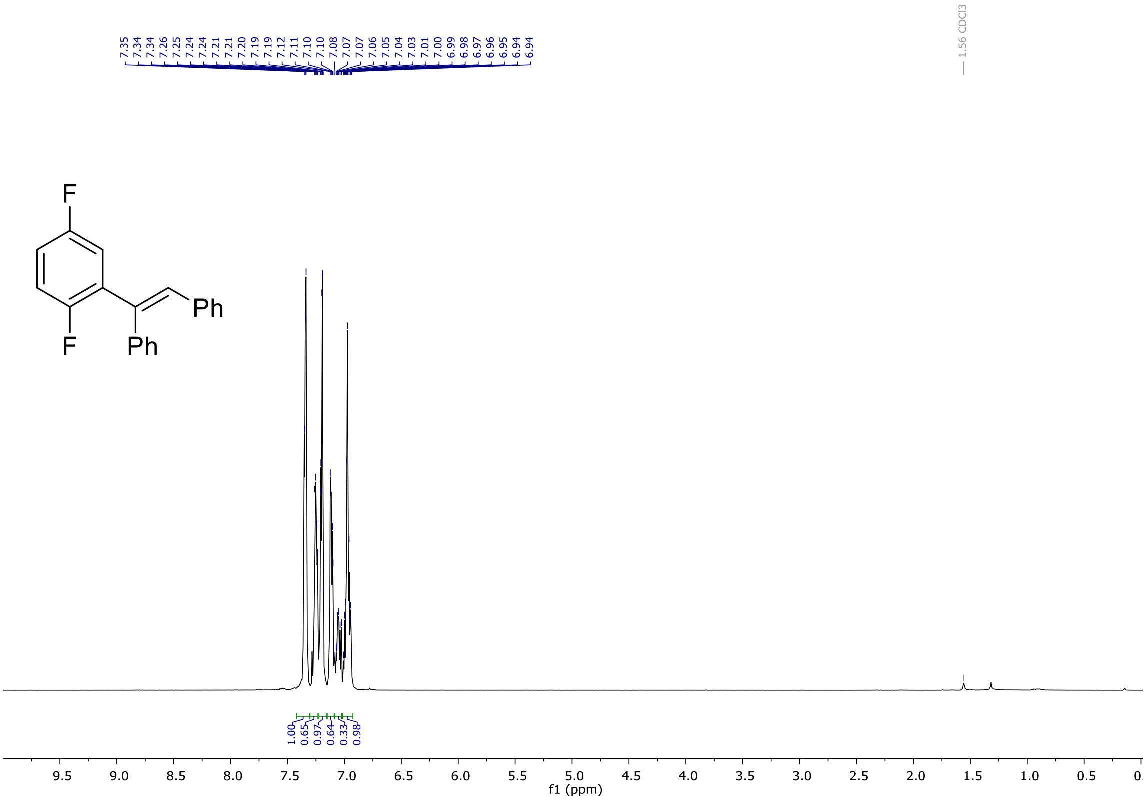

^{19}F NMR (376MHz, $CDCl_3$)

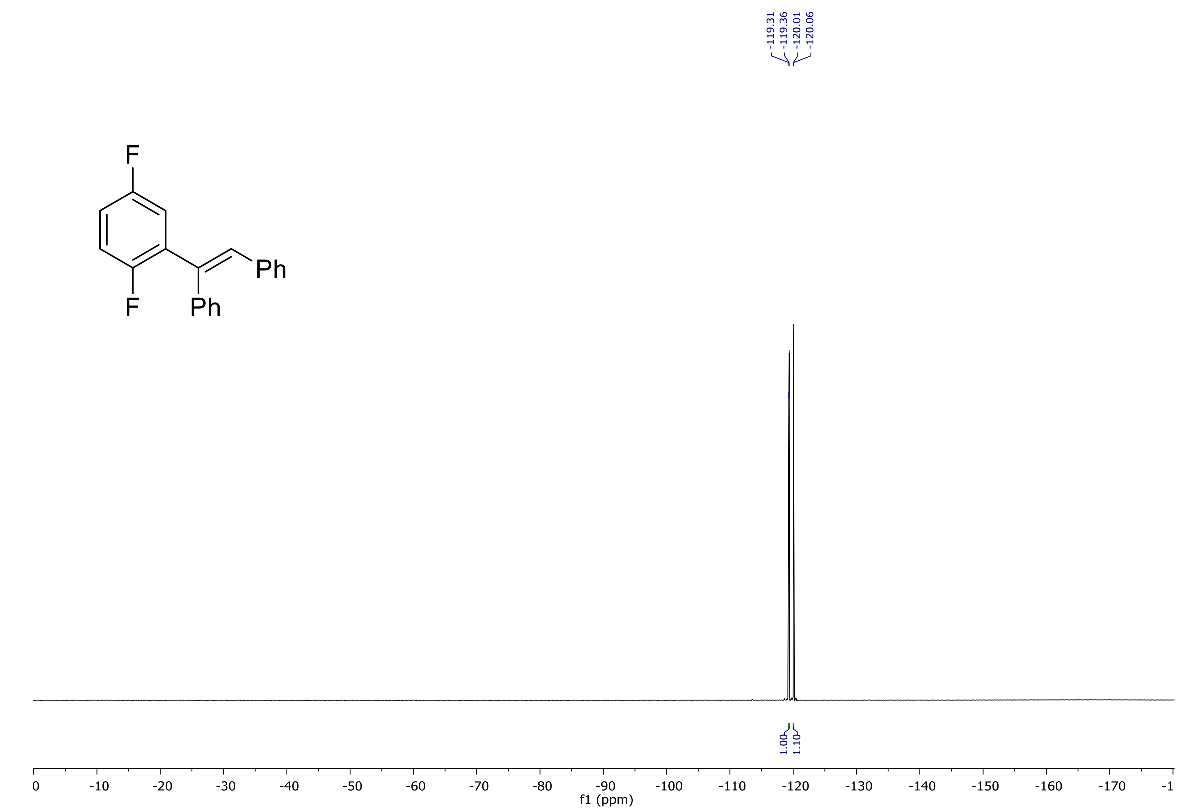

^{13}C NMR (101MHz, $CDCl_3$)

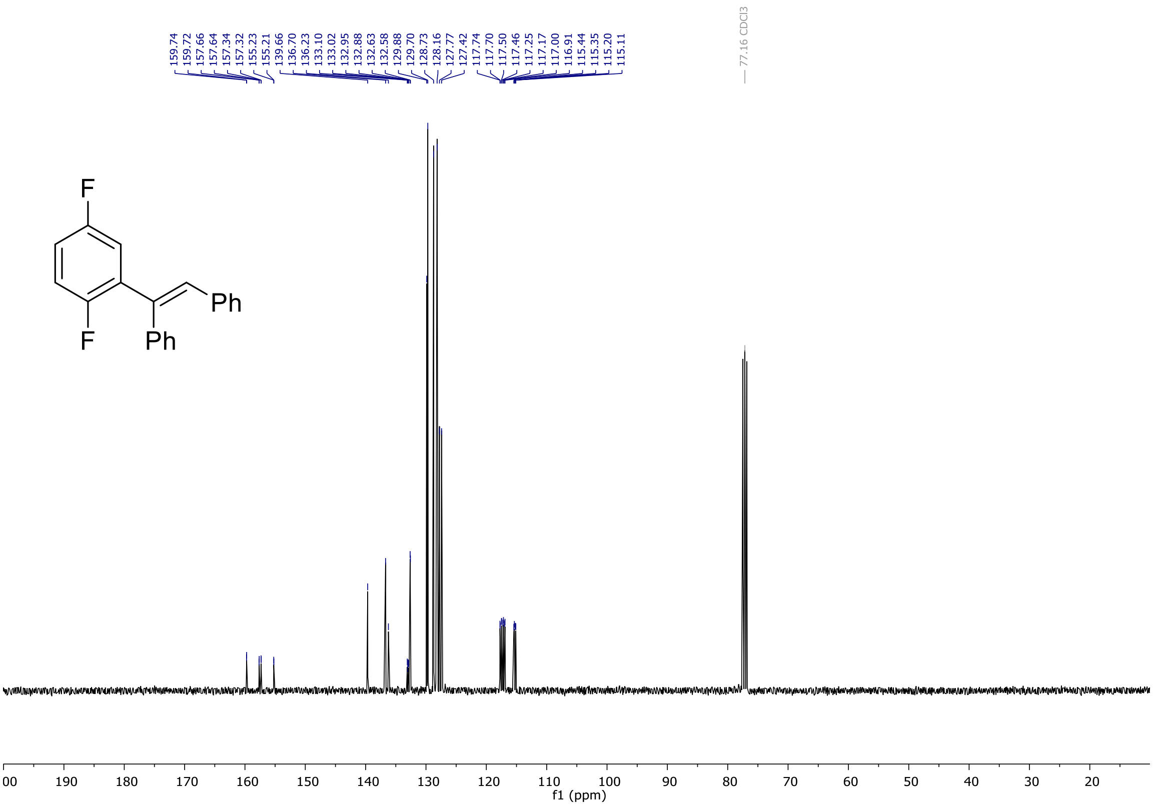

¹H NMR (500MHz, $CDCl_3$)

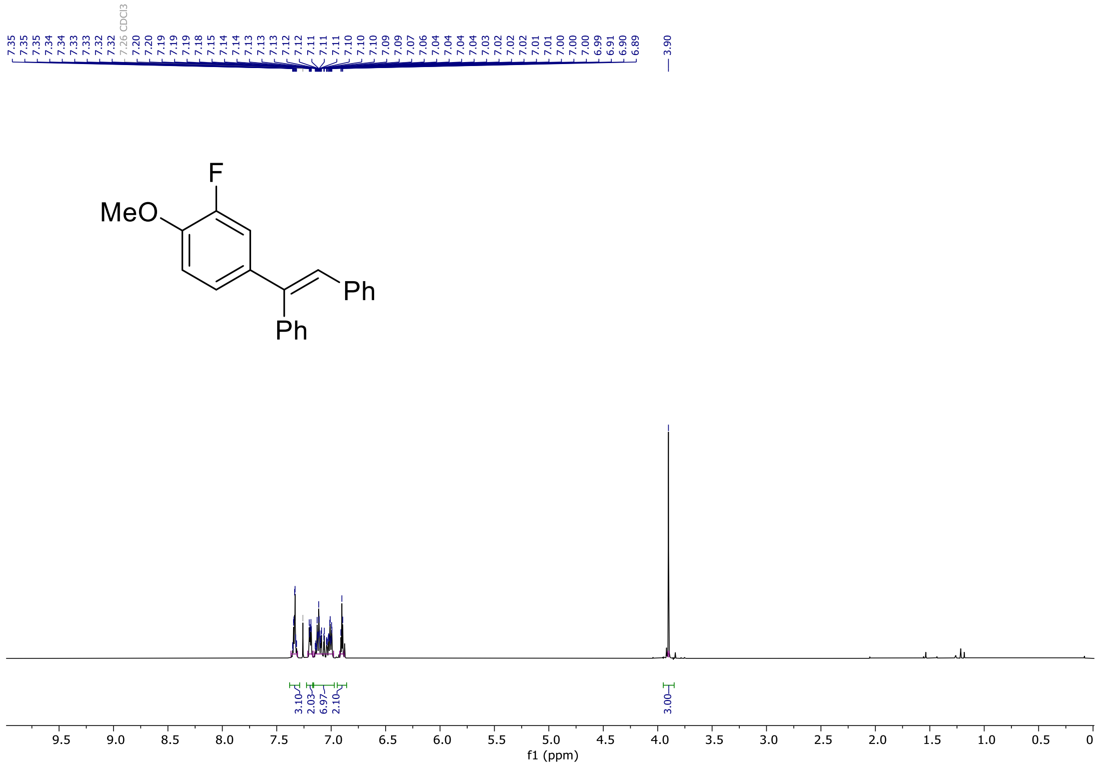

¹³C NMR (126MHz, $CDCl_3$)

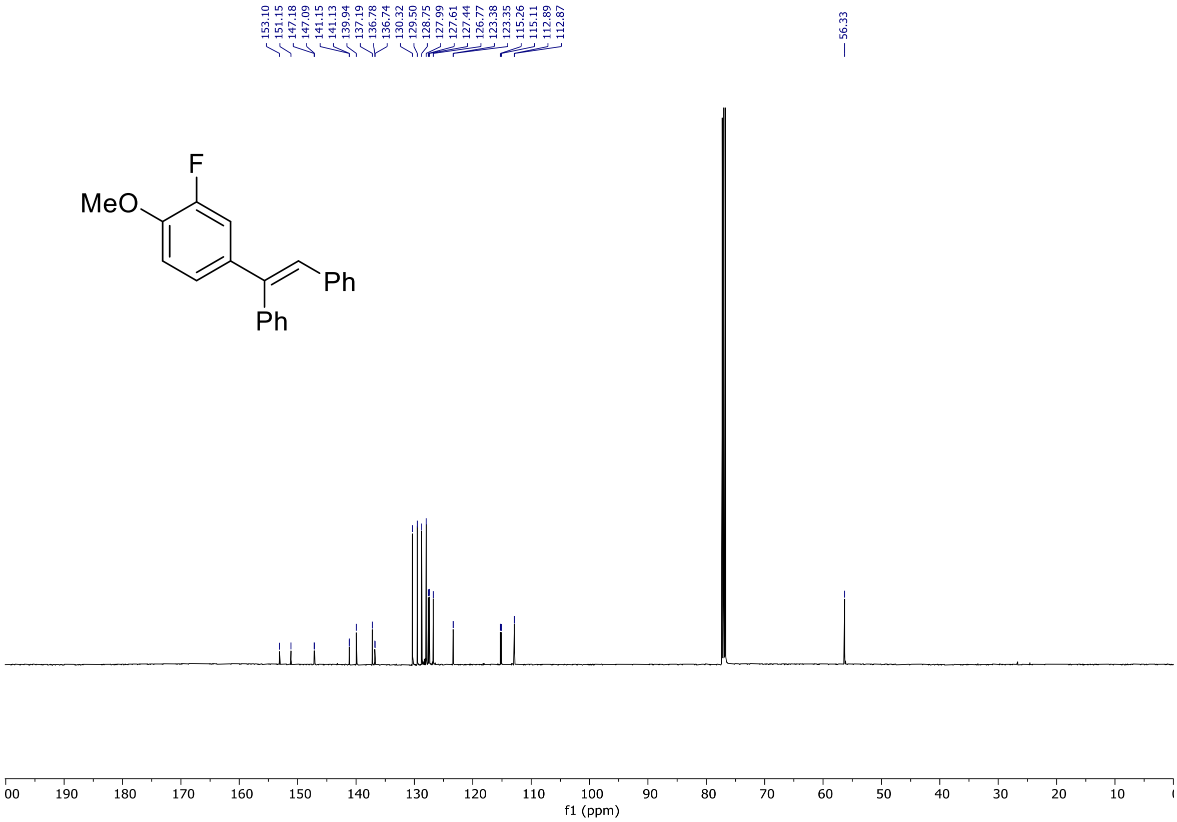

^{1}H NMR (400MHz, $CDCl_3$)

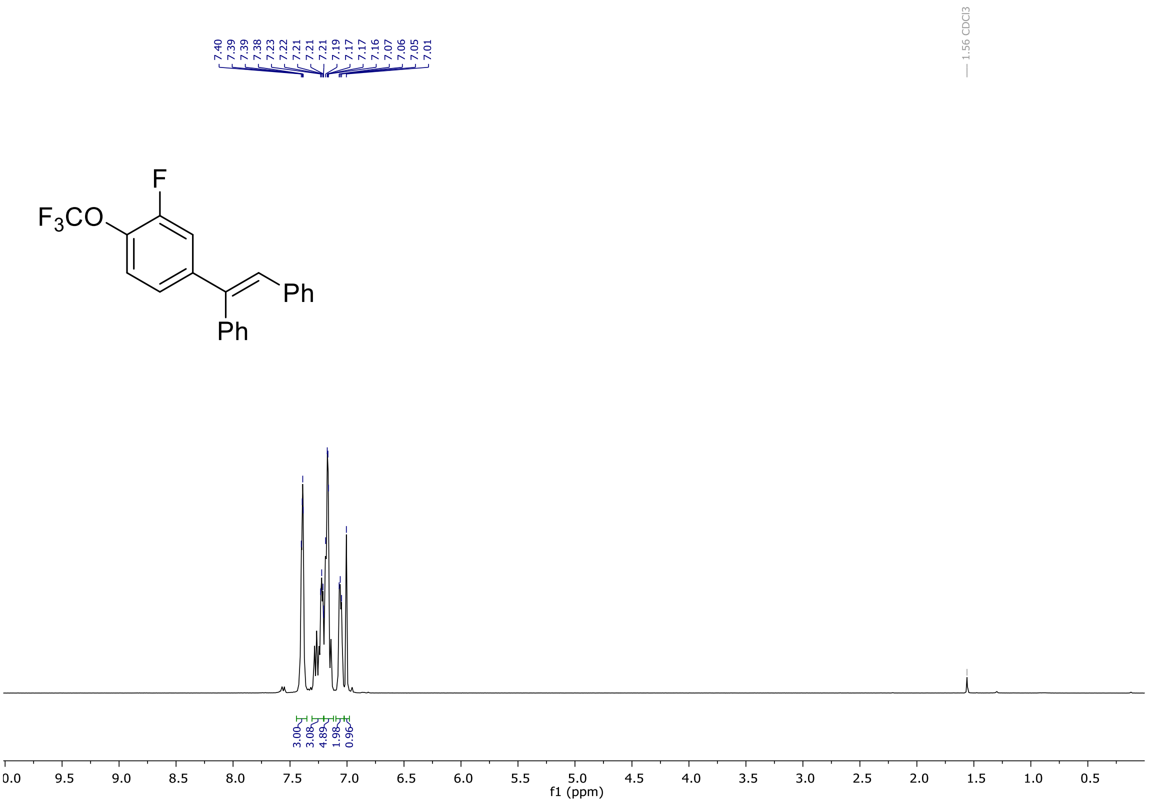

^{19}F NMR (376MHz, $CDCl_3$)

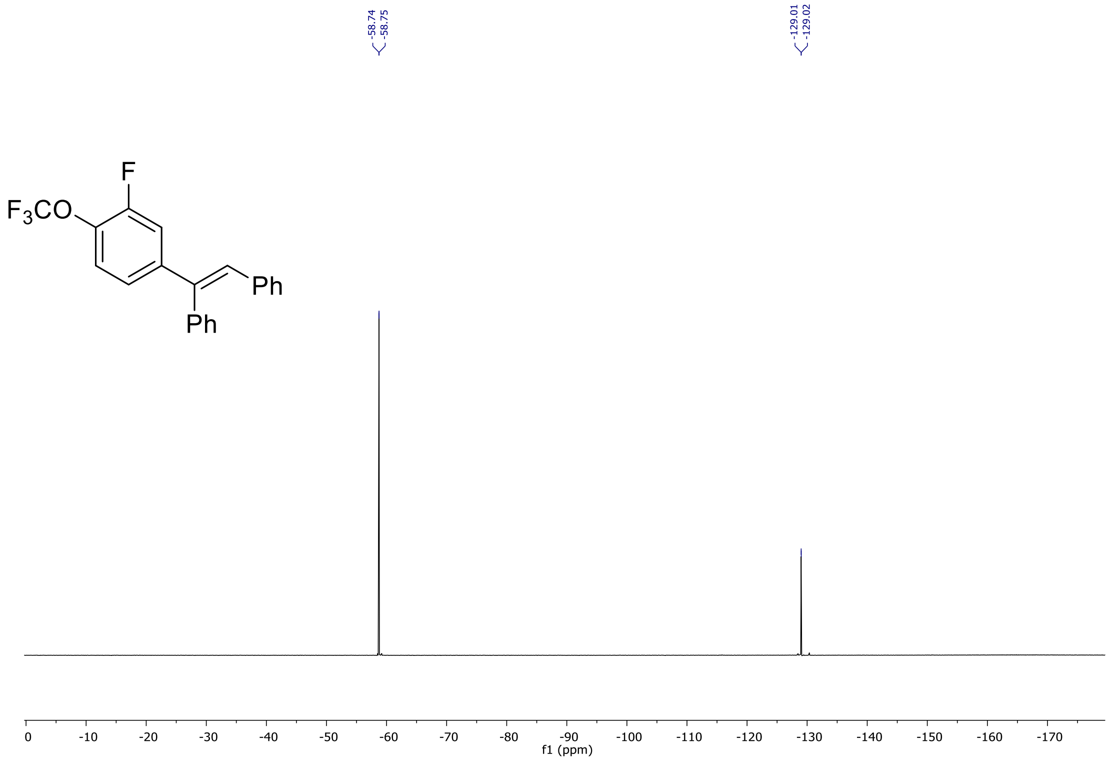

^{13}C NMR (101MHz, $CDCl_3$)

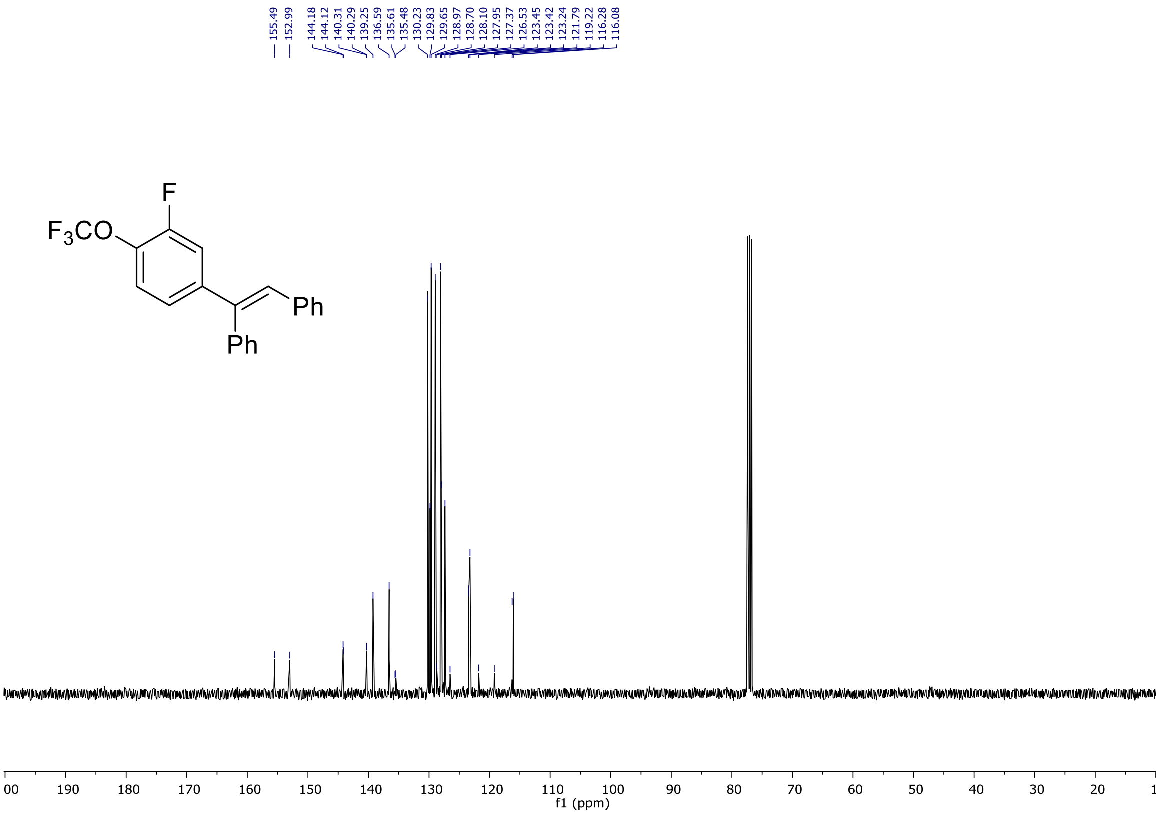

^{1}H NMR (400MHz, $CDCl_3$)

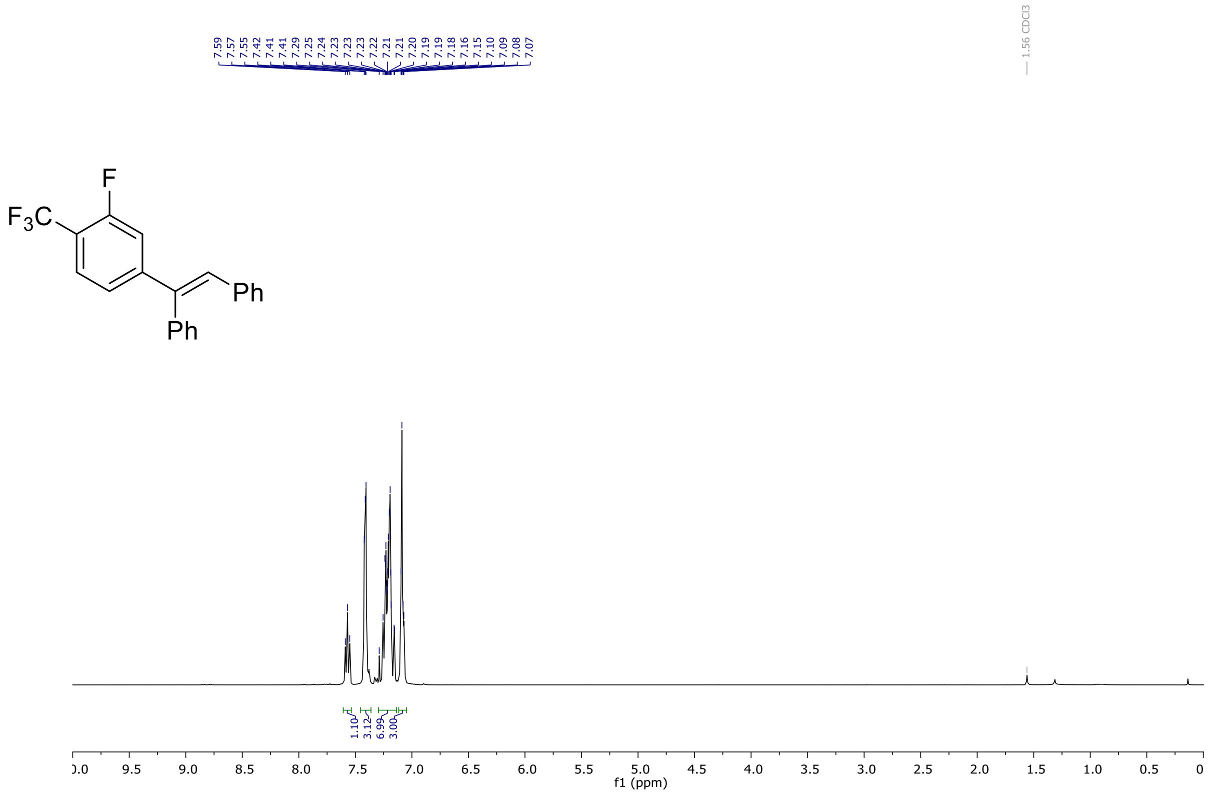

^{19}F NMR (376MHz, $CDCl_3$)

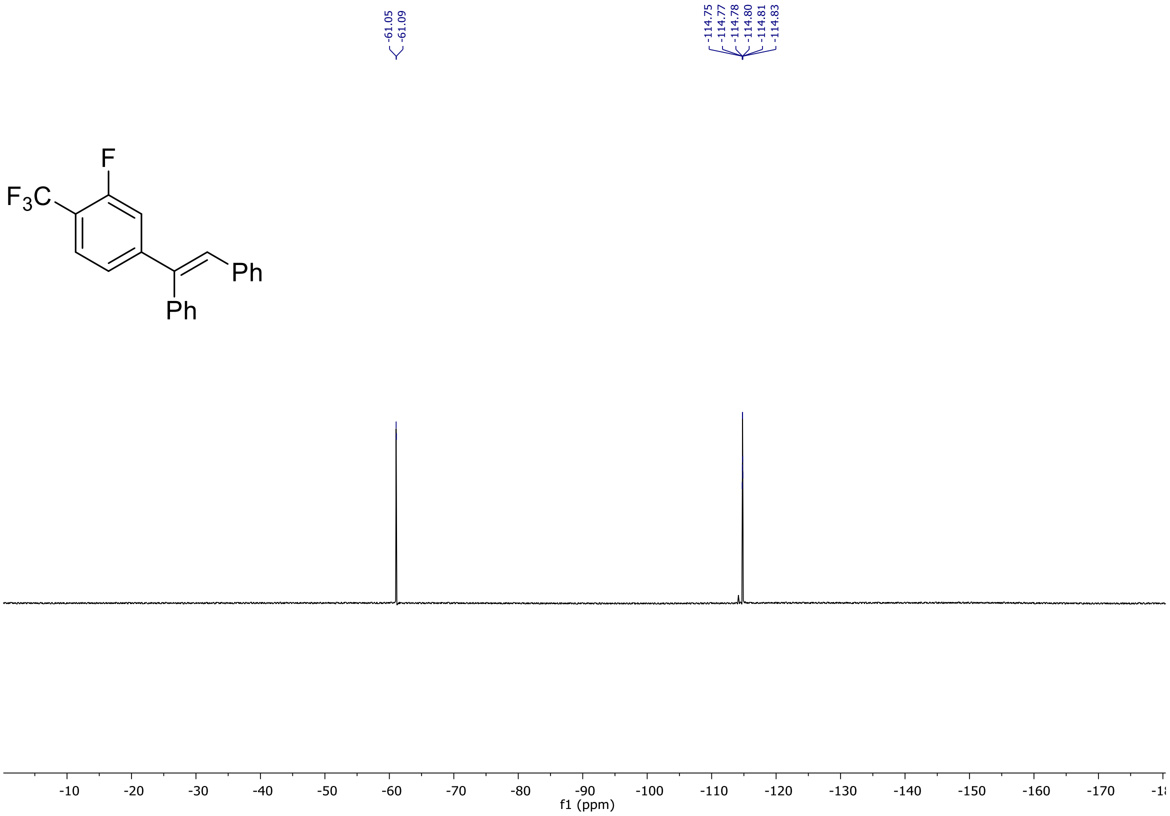

^{13}C NMR (126MHz, $CDCl_3$)

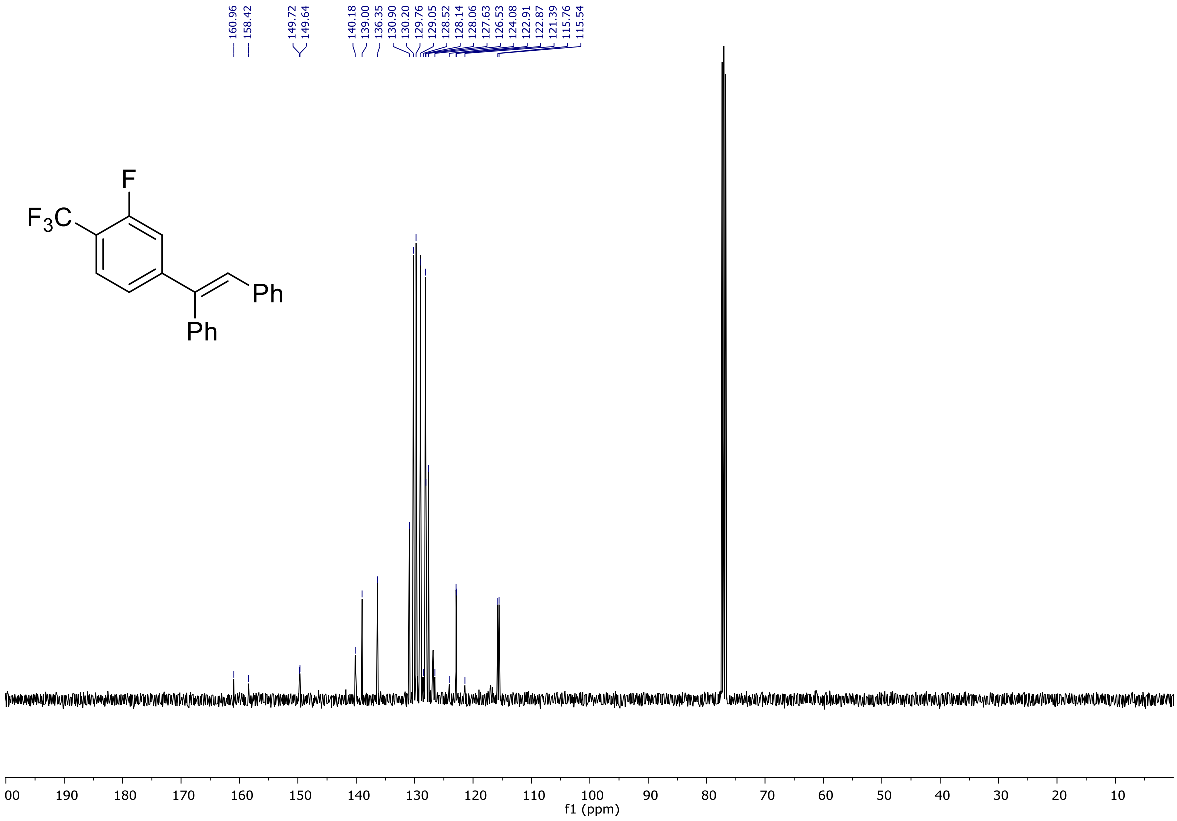

¹H NMR (400MHz, $CDCl_3$)

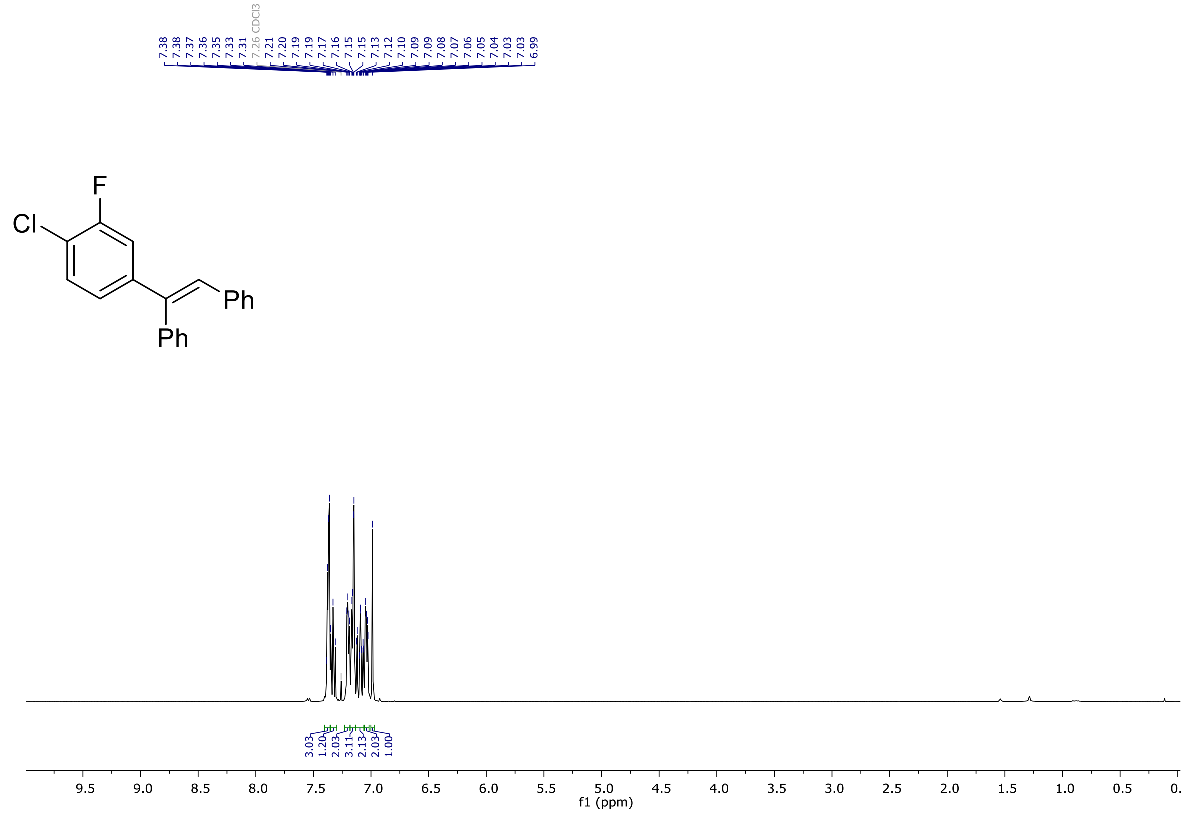

¹⁹F NMR (376MHz, $CDCl_3$)

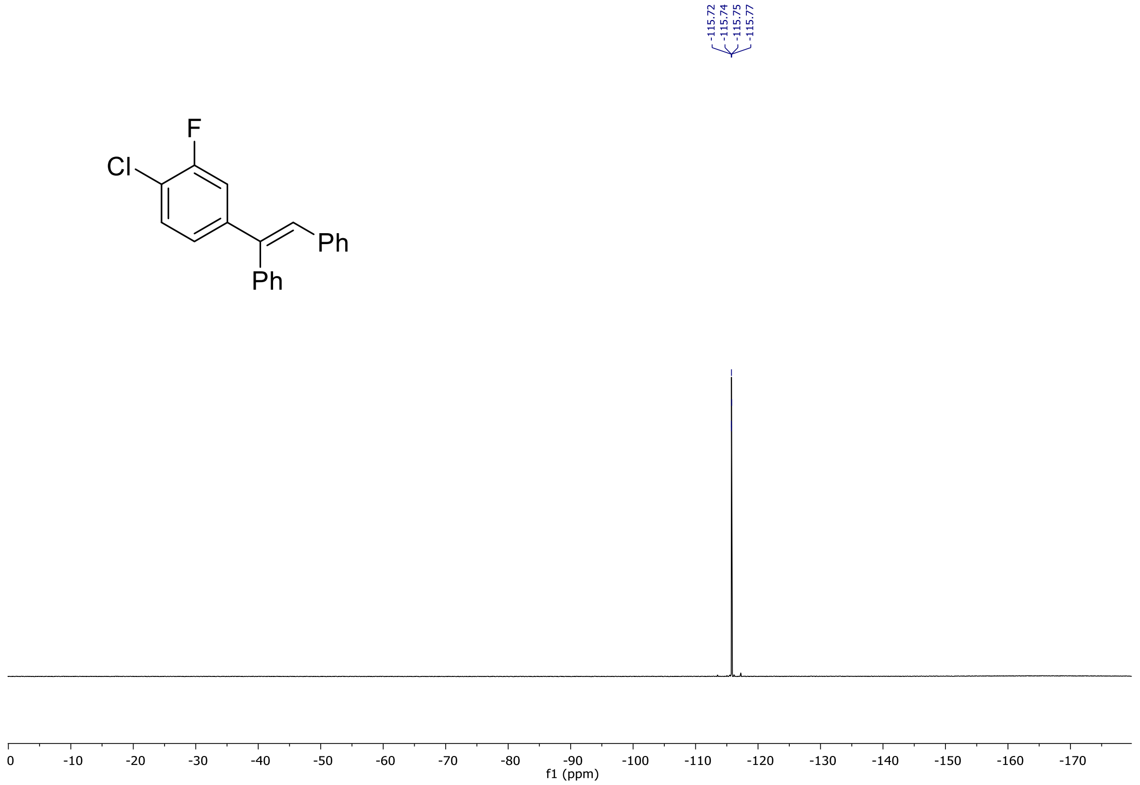

^{13}C NMR (101MHz, $CDCl_3$)

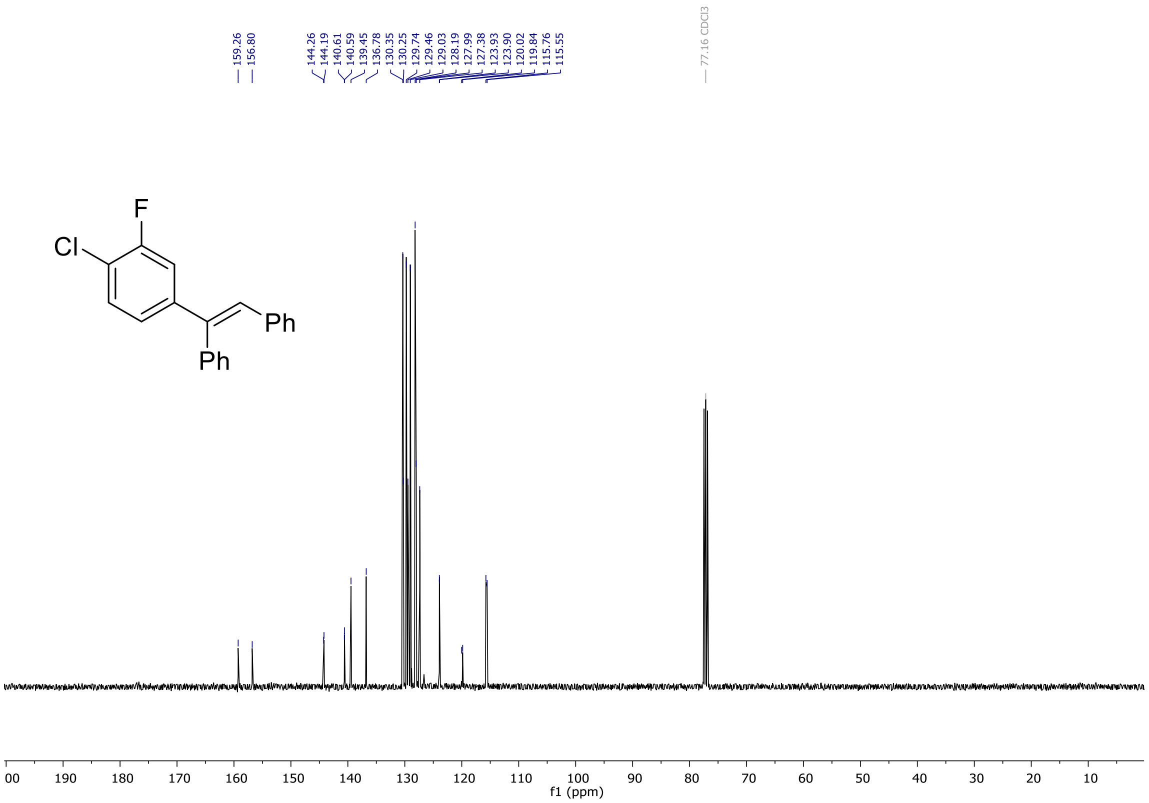

^{1}H NMR (400MHz, $CDCl_3$)

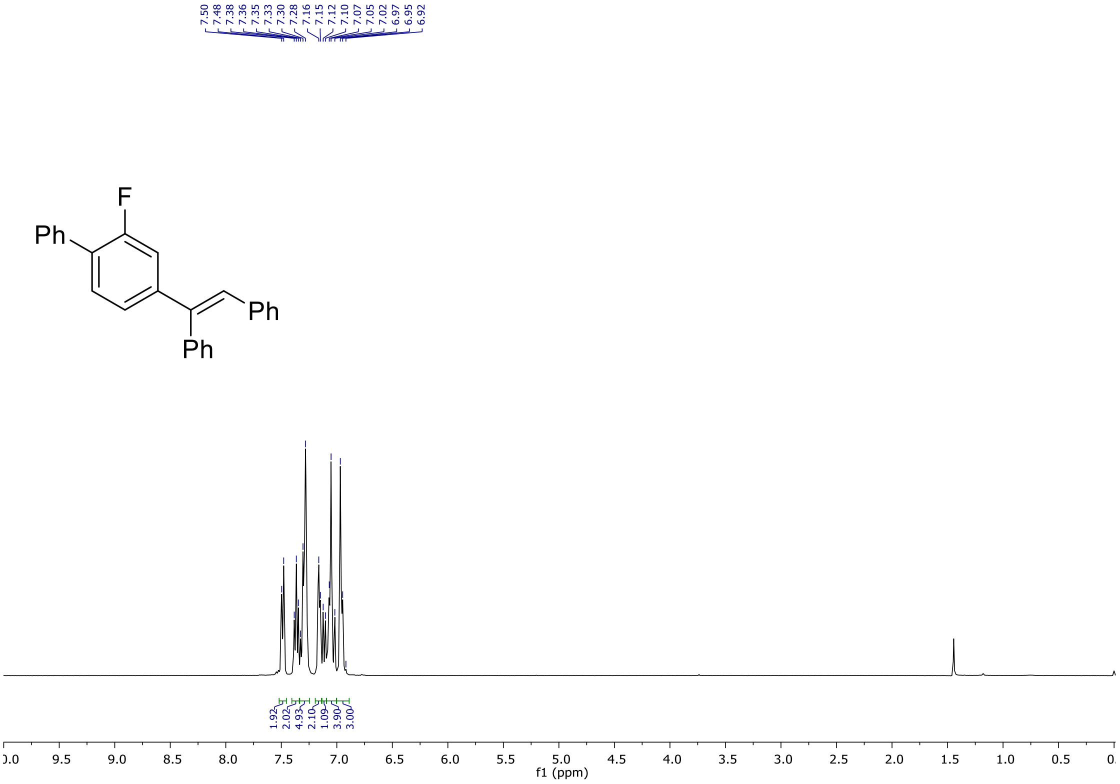

^{19}F NMR (376MHz, $CDCl_3$)

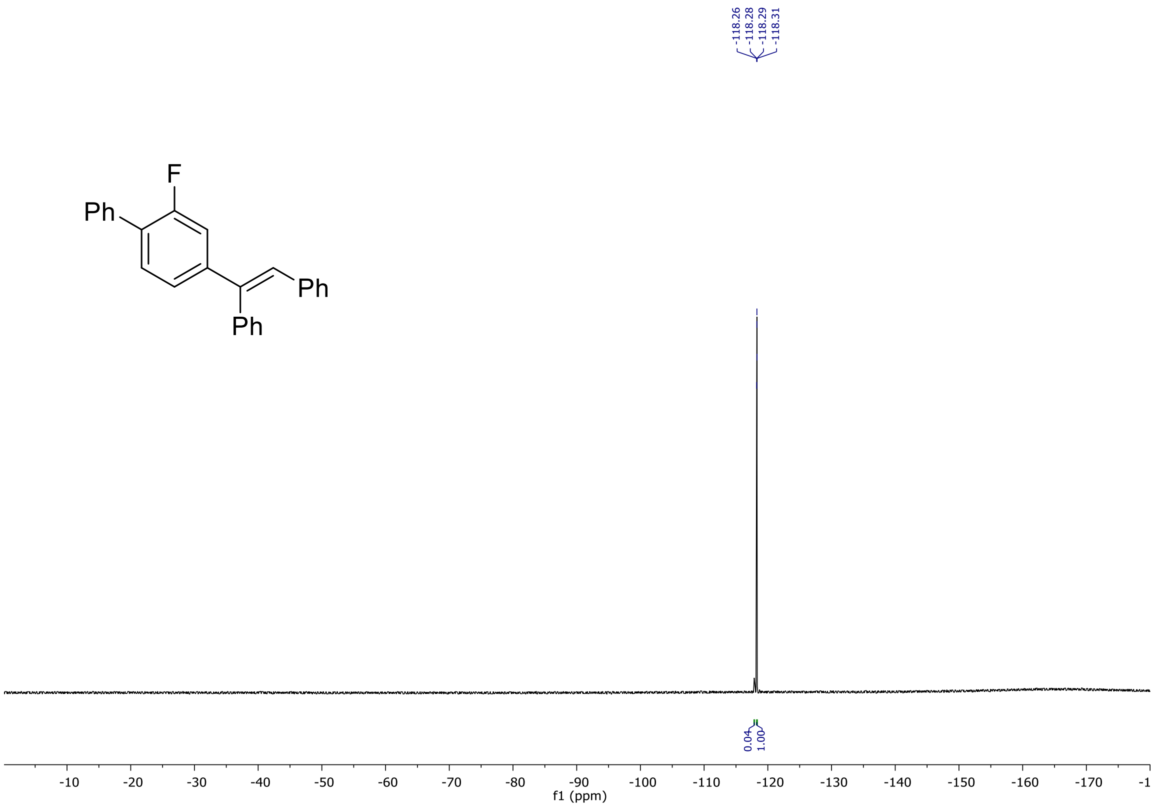

^{13}C NMR (101MHz, $CDCl_3$)

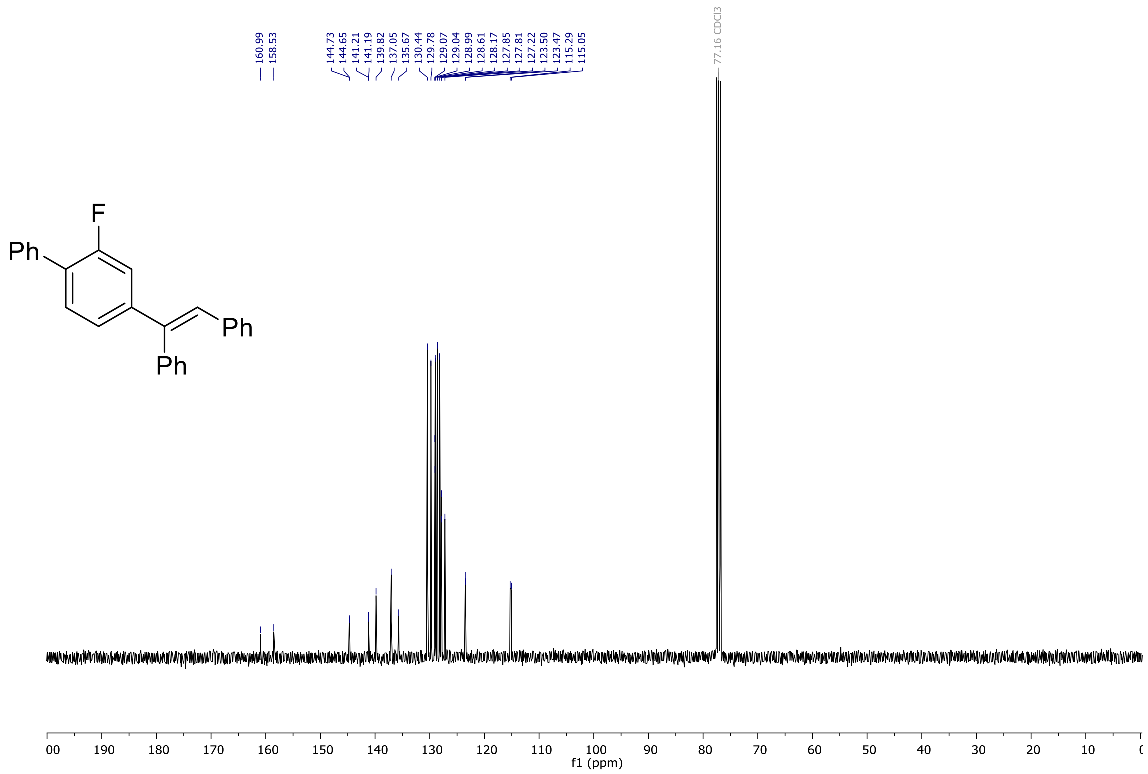

^{1}H NMR (400MHz, $CDCl_3$)

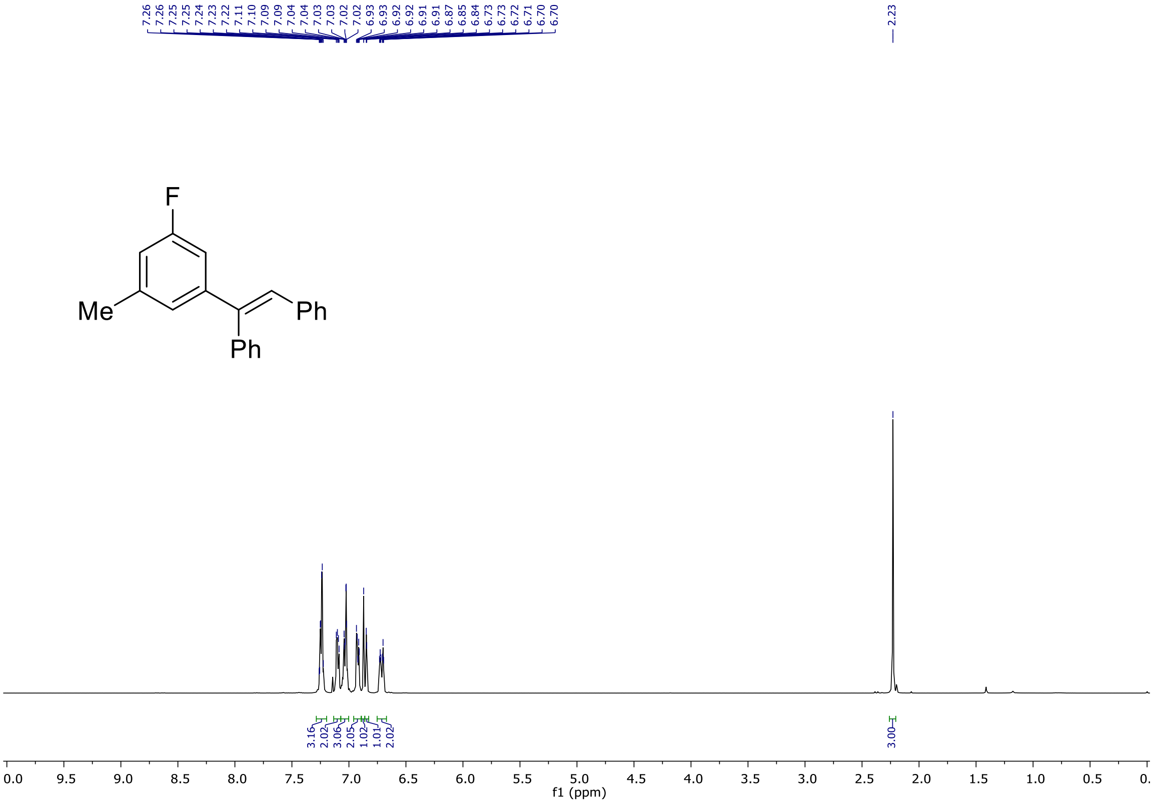

^{19}F NMR (376MHz, $CDCl_3$)

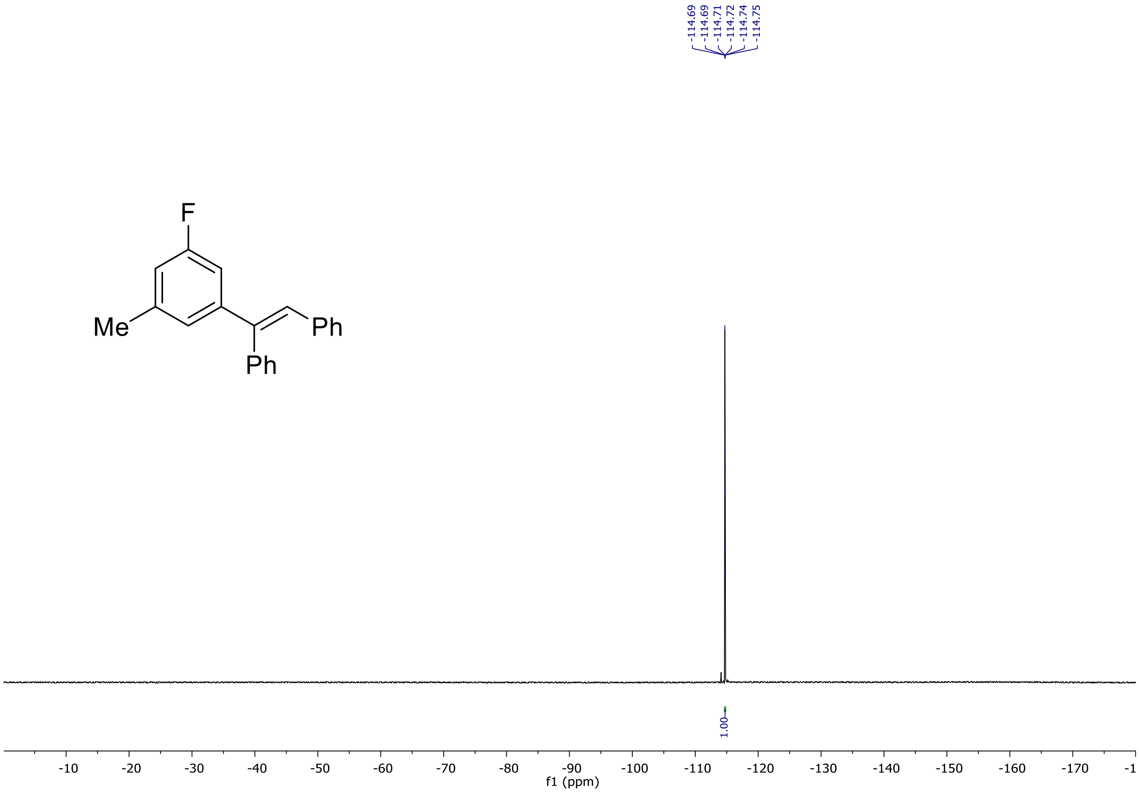

^{13}C NMR (126MHz, $CDCl_3$)

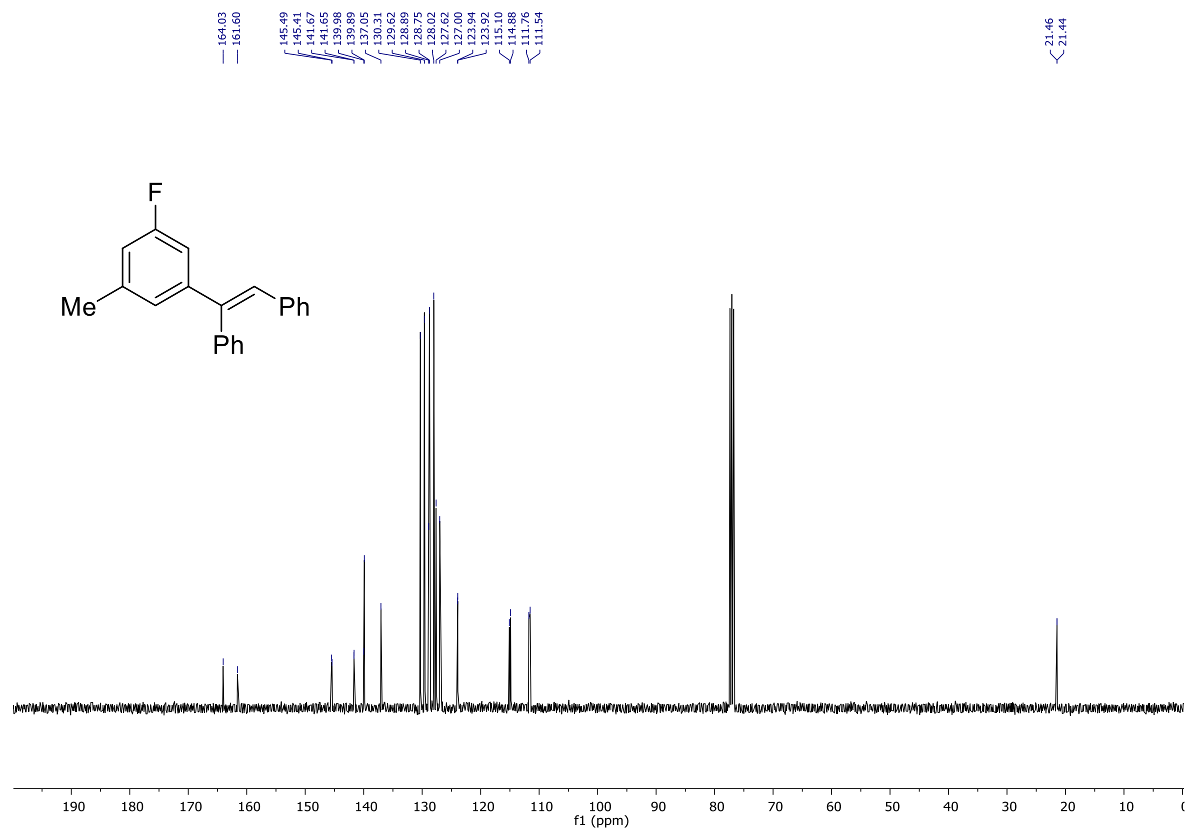

^{1}H NMR (500MHz, $CDCl_3$)

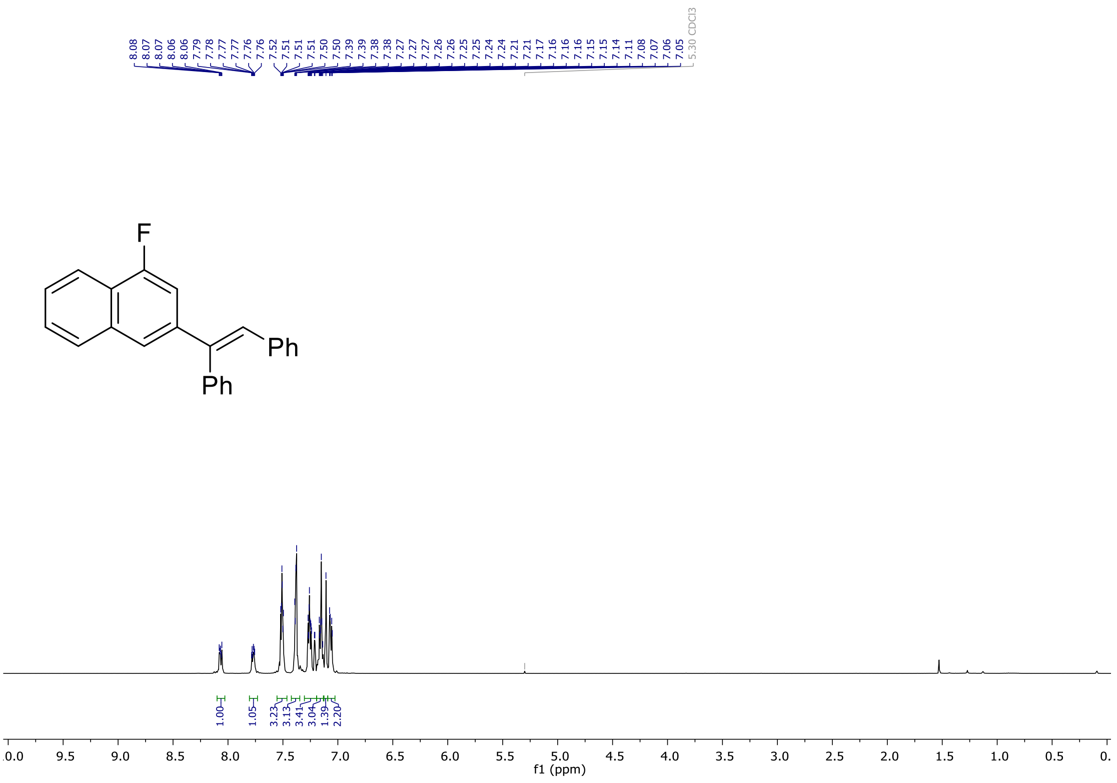

^{19}F NMR (376MHz, $CDCl_3$)

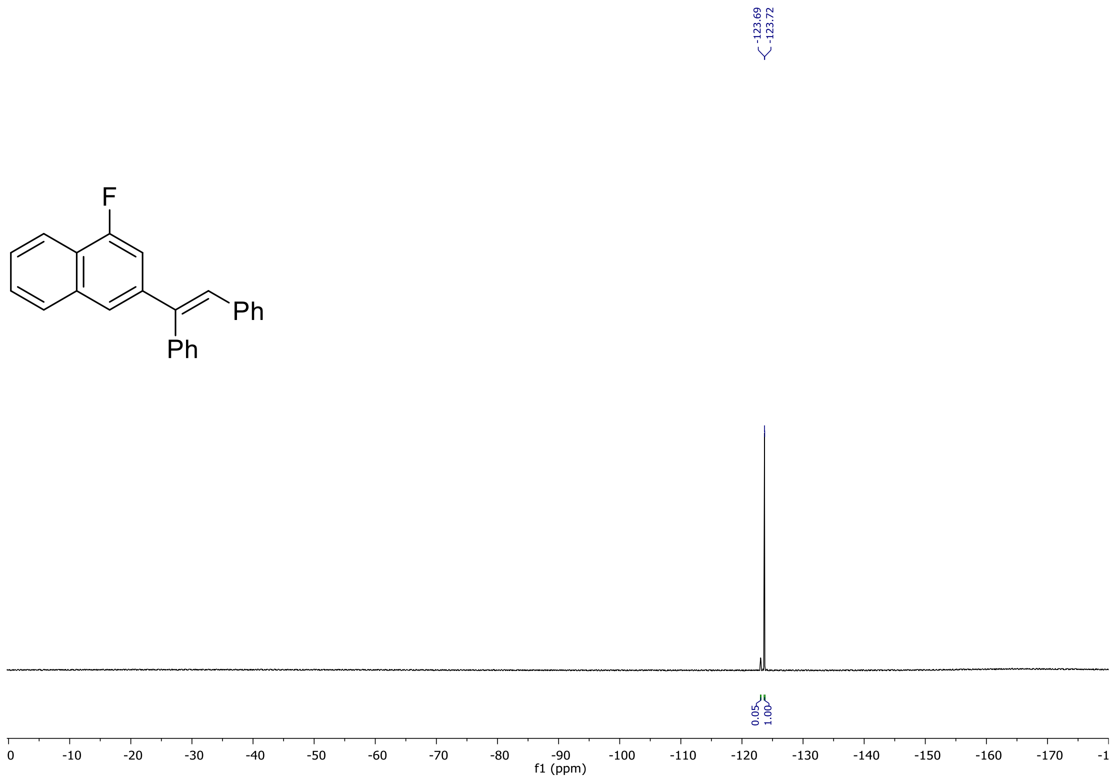

^{13}C NMR (126MHz, $CDCl_3$)

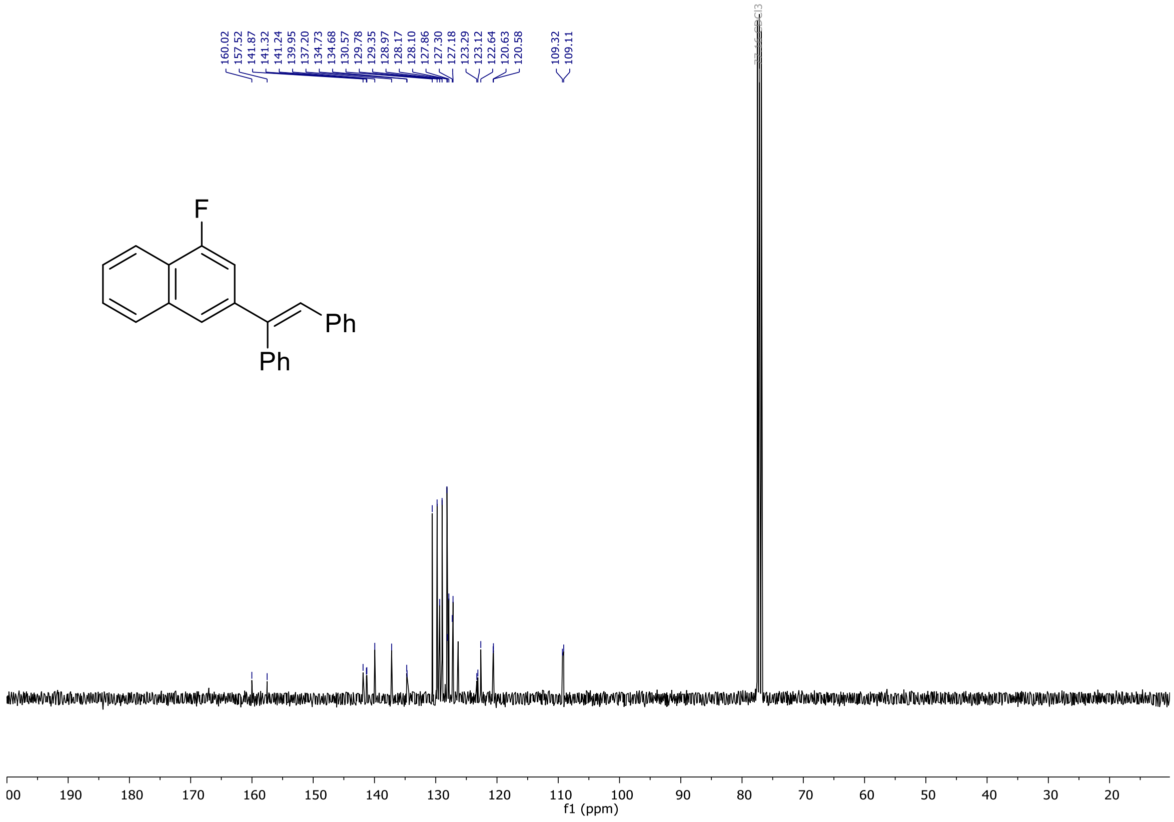

^{1}H NMR (400MHz, $CDCl_3$)

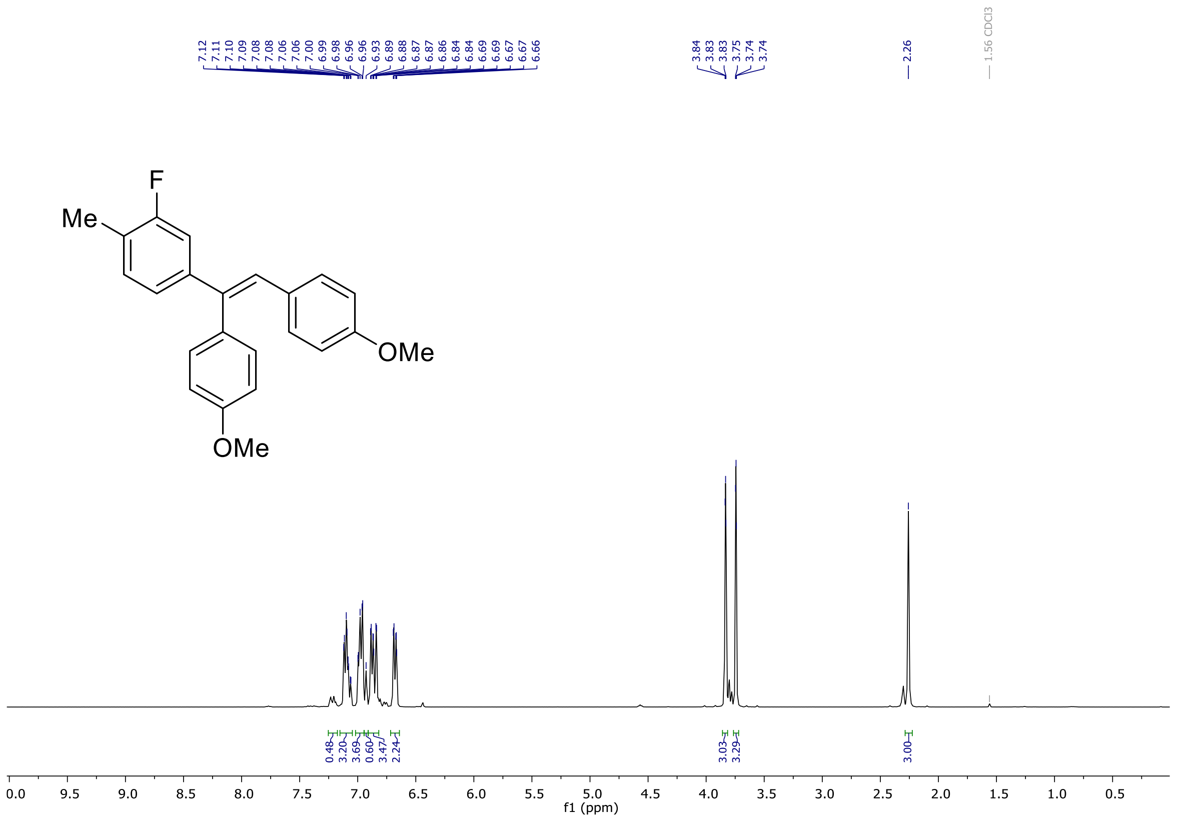

^{19}F NMR (376MHz, $CDCl_3$)

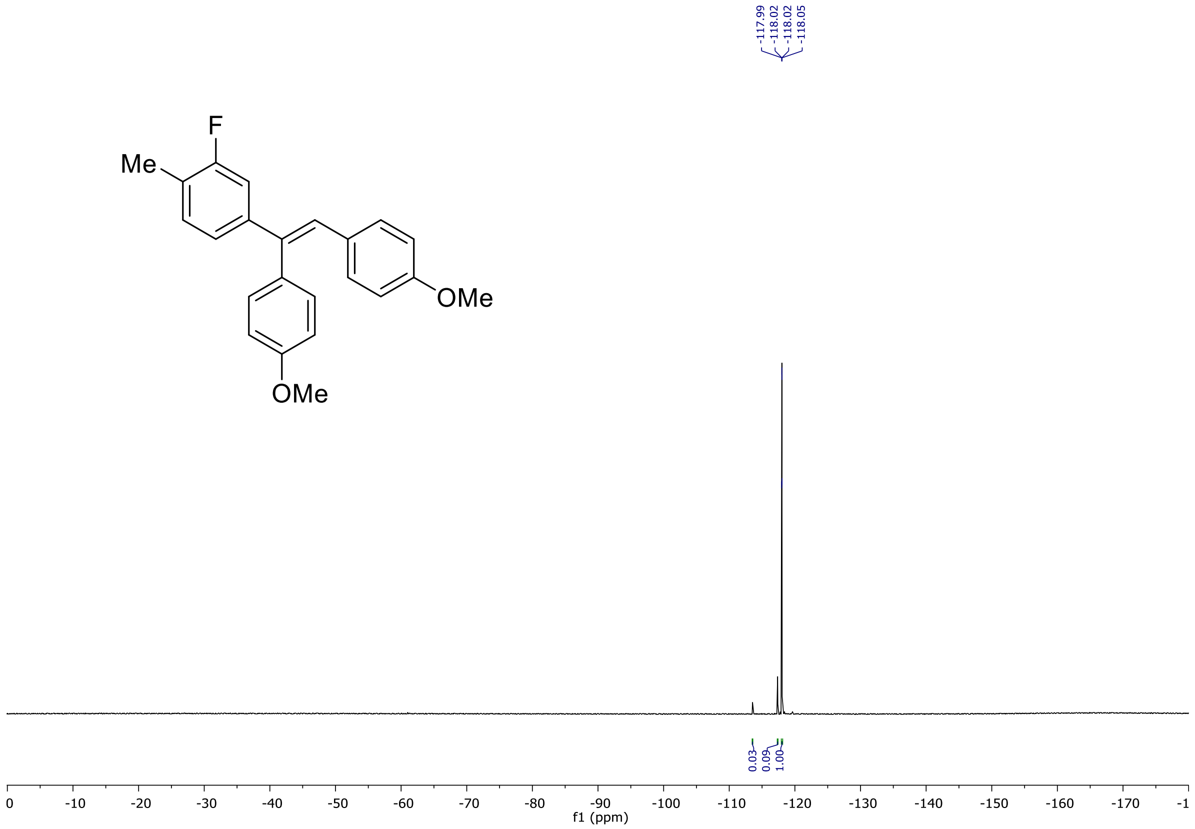

^{13}C NMR (126MHz, $CDCl_3$)

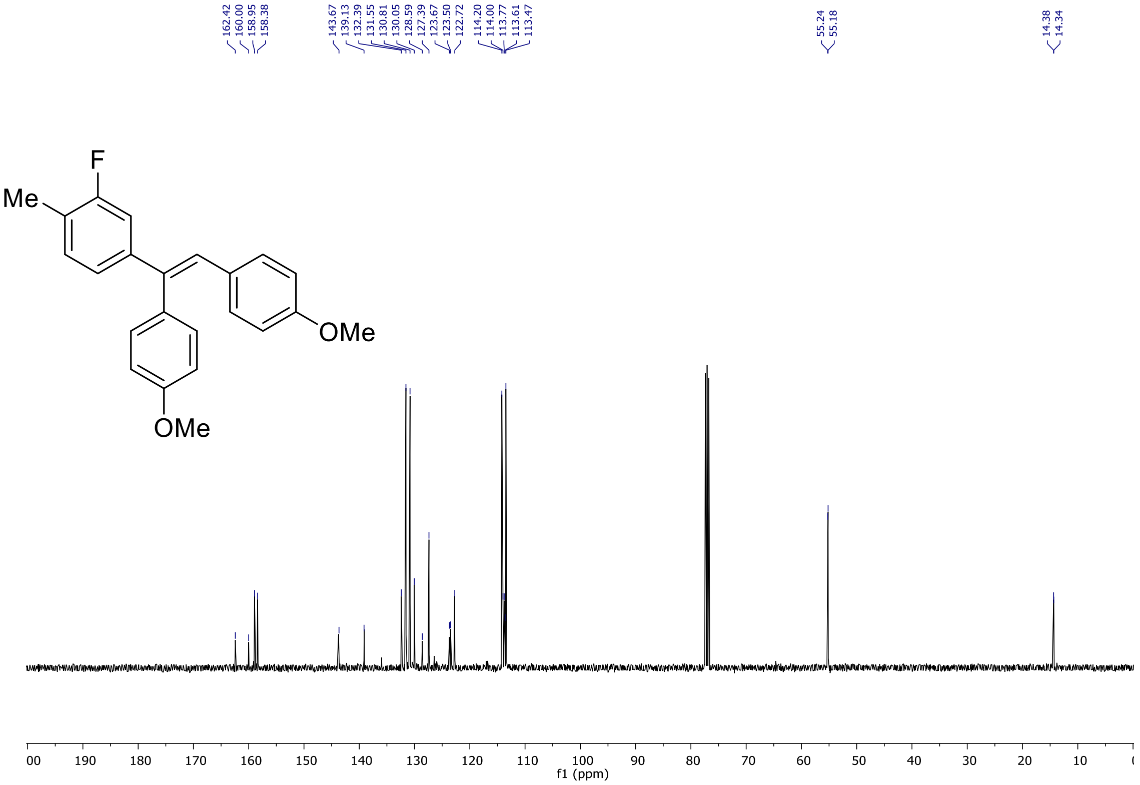

^{1}H NMR (400MHz, $CDCl_3$)

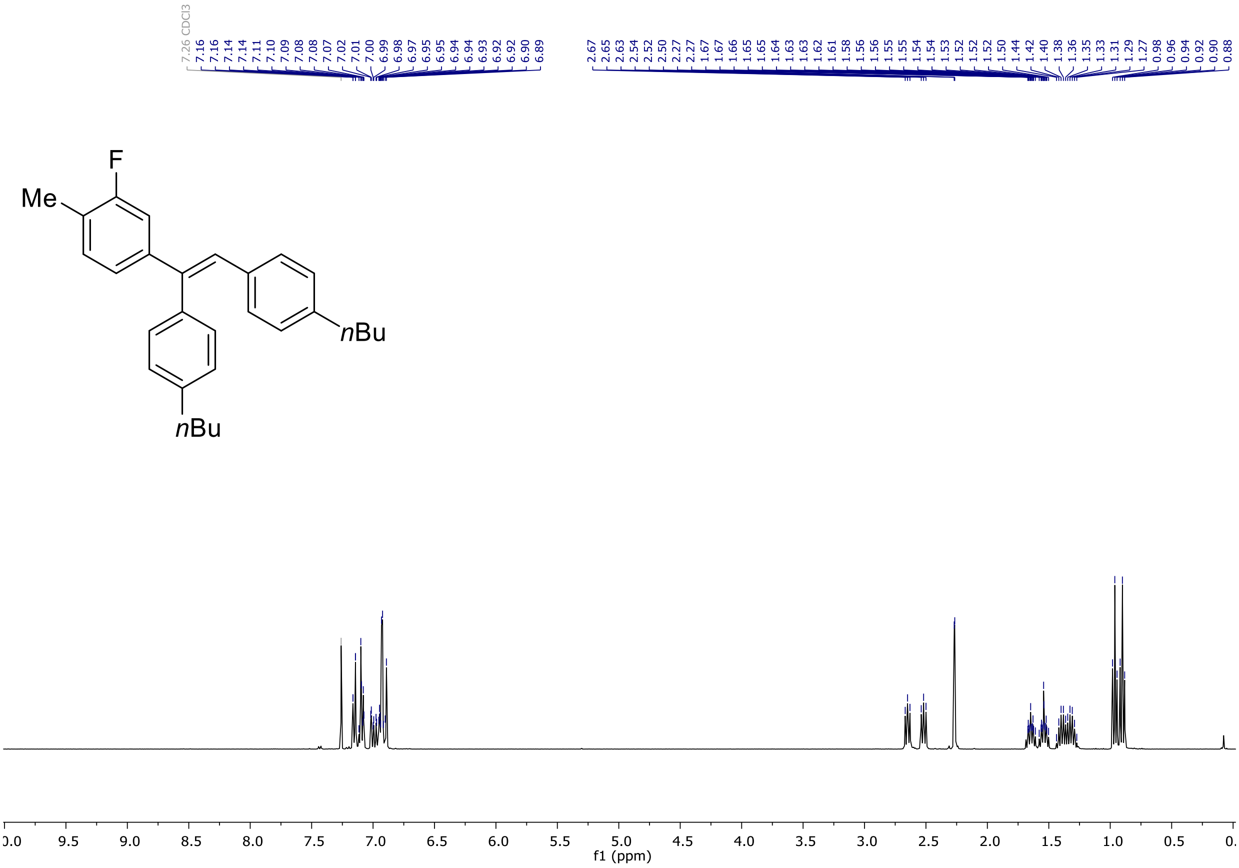

^{19}F NMR (376MHz, $CDCl_3$)

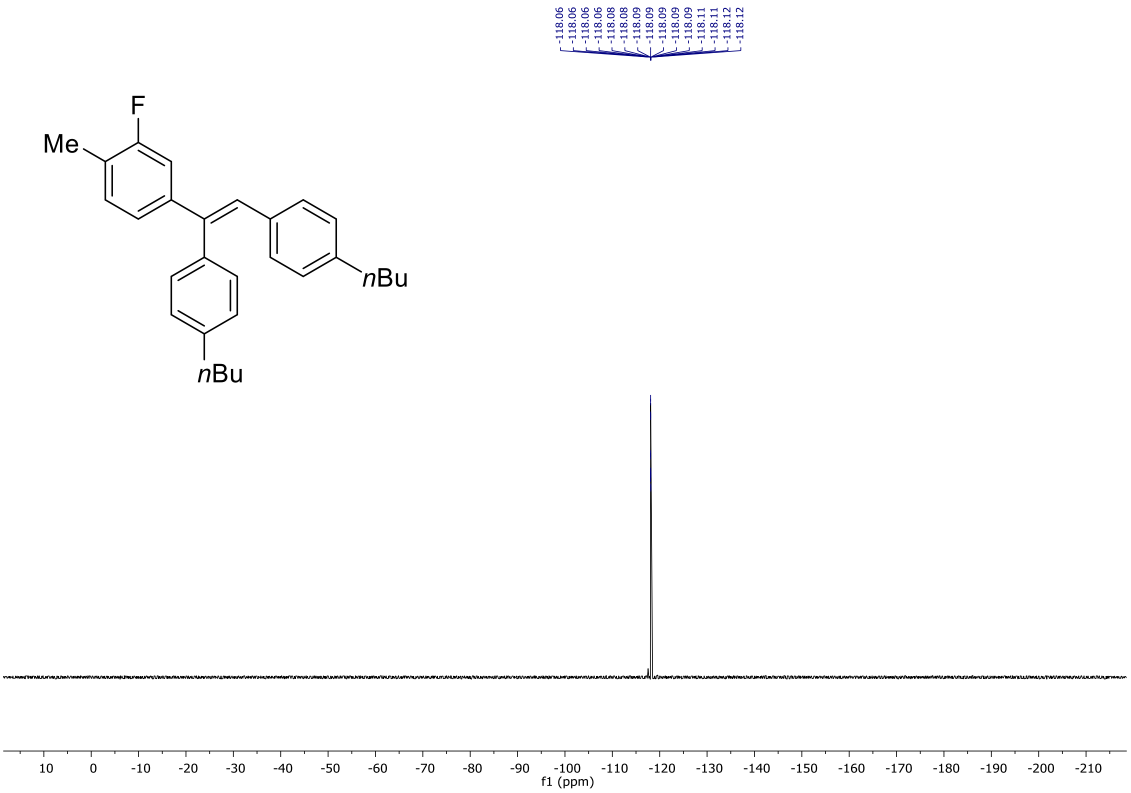

^{13}C NMR (101MHz, $CDCl_3$)

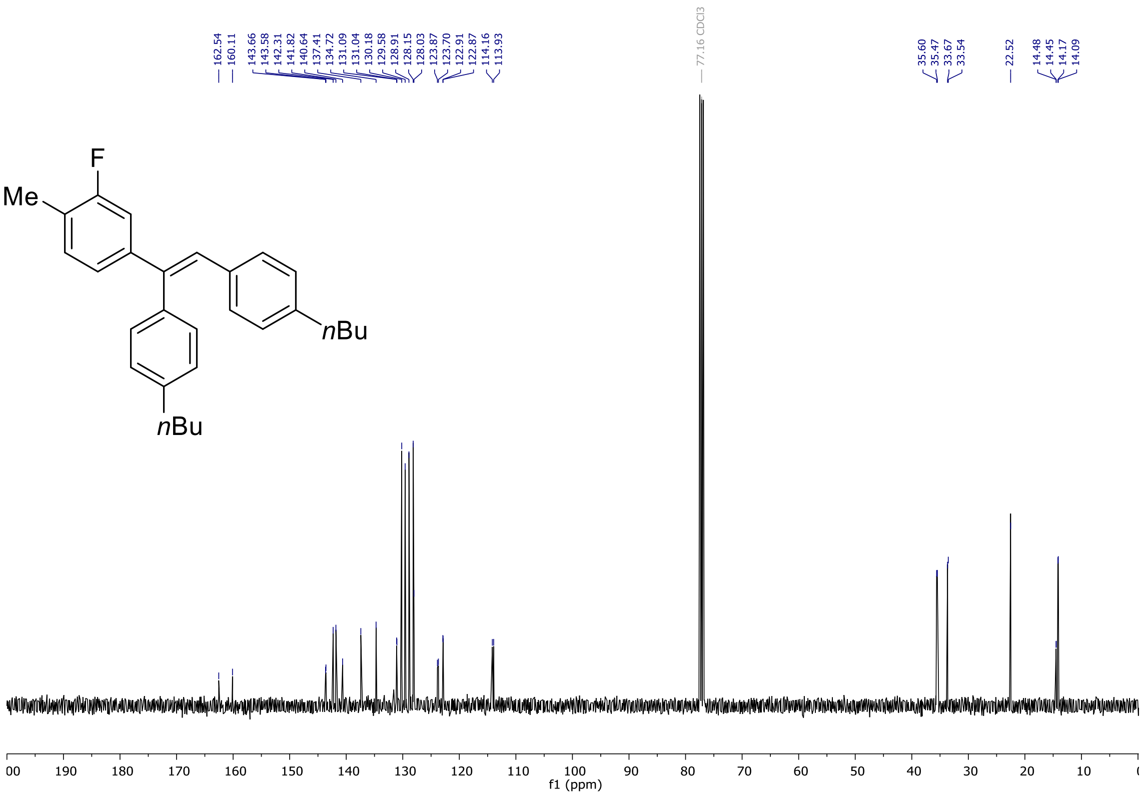

^{1}H NMR (400MHz, $CDCl_3$)

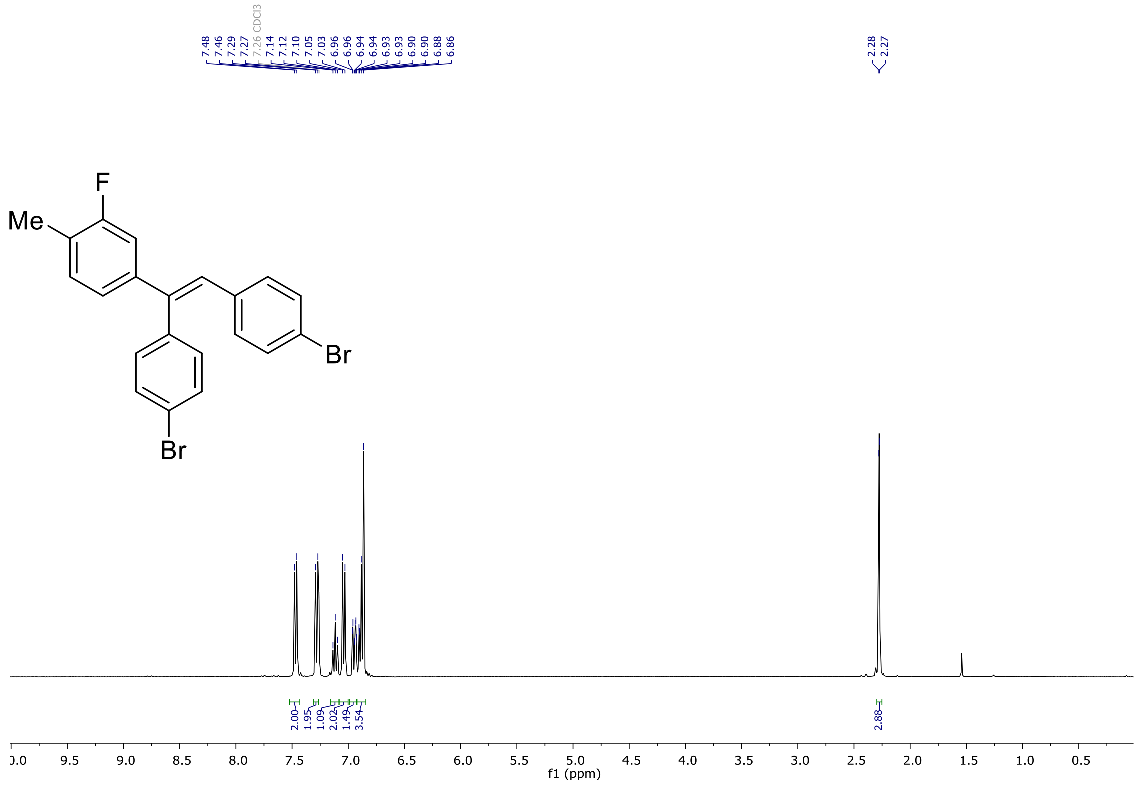

^{19}F NMR (376MHz, $CDCl_3$)

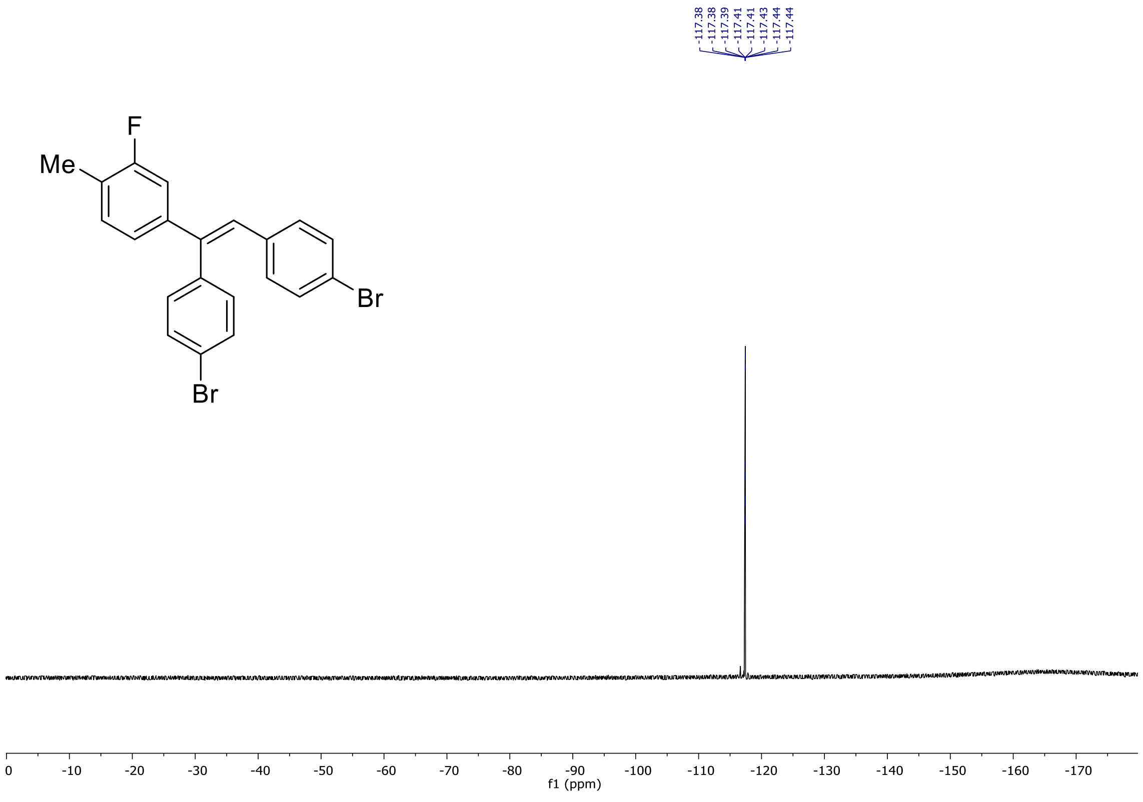

^{13}C NMR (126MHz, $CDCl_3$)

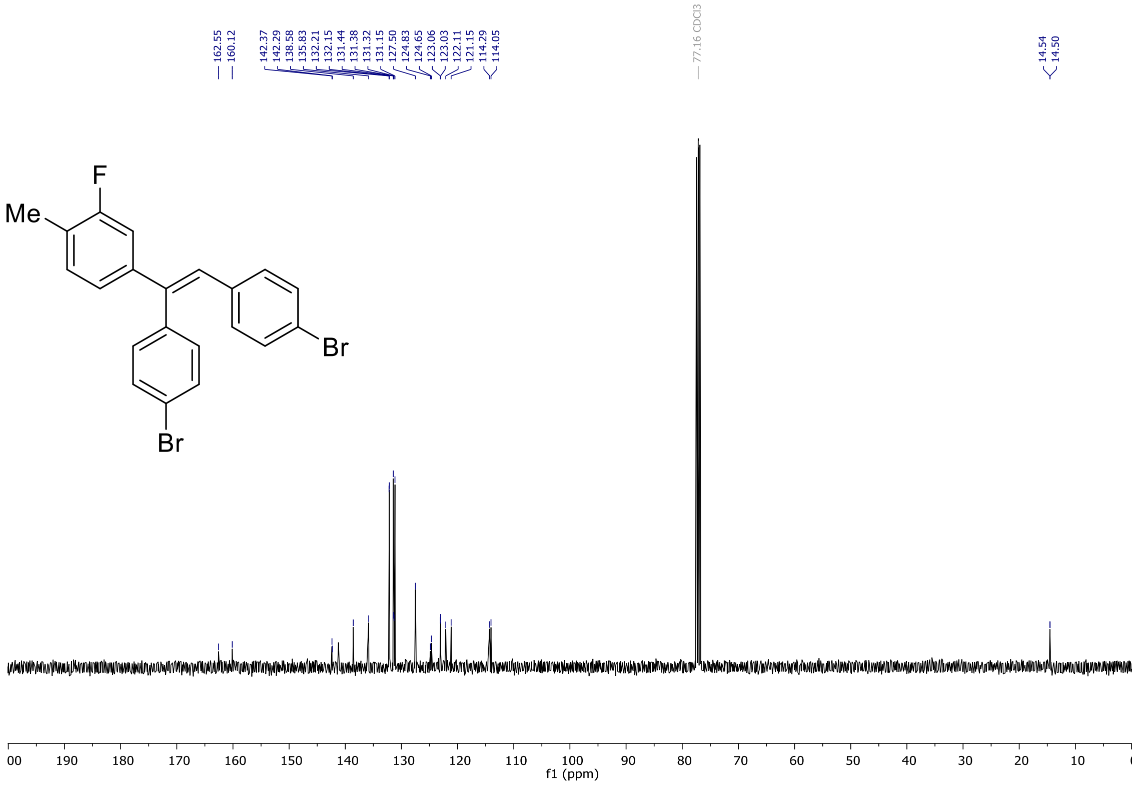

^{1}H NMR (400MHz, $CDCl_3$)

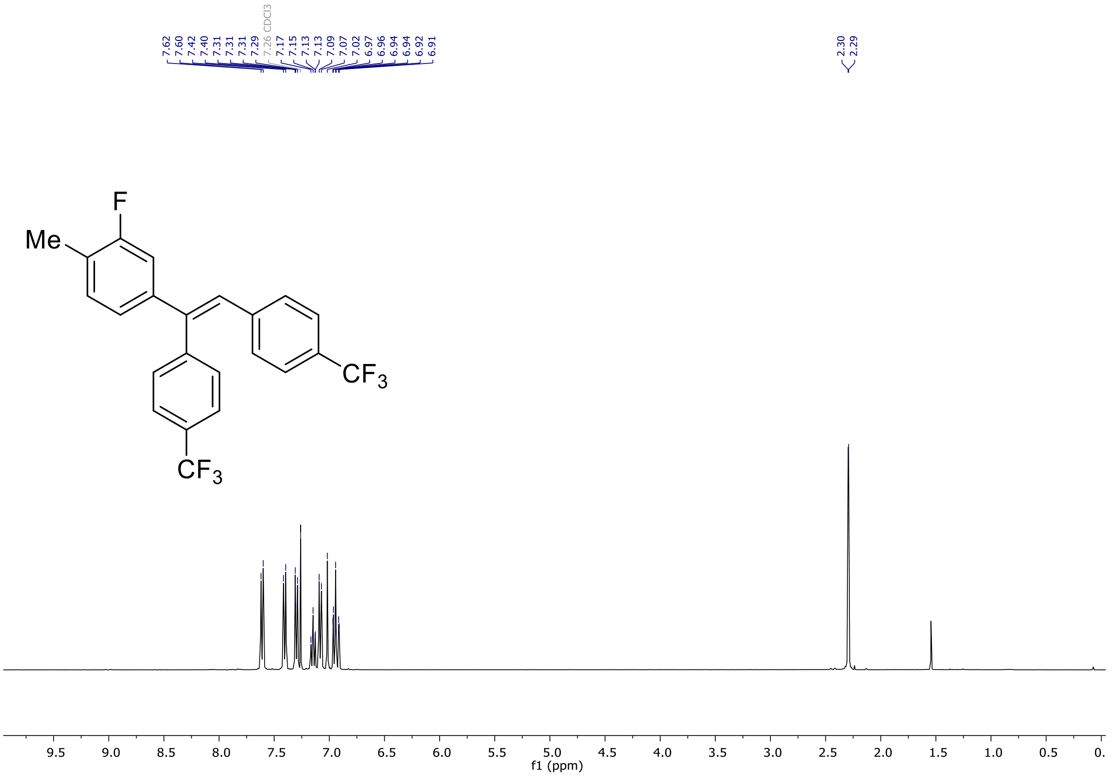

^{19}F NMR (376MHz, $CDCl_3$)

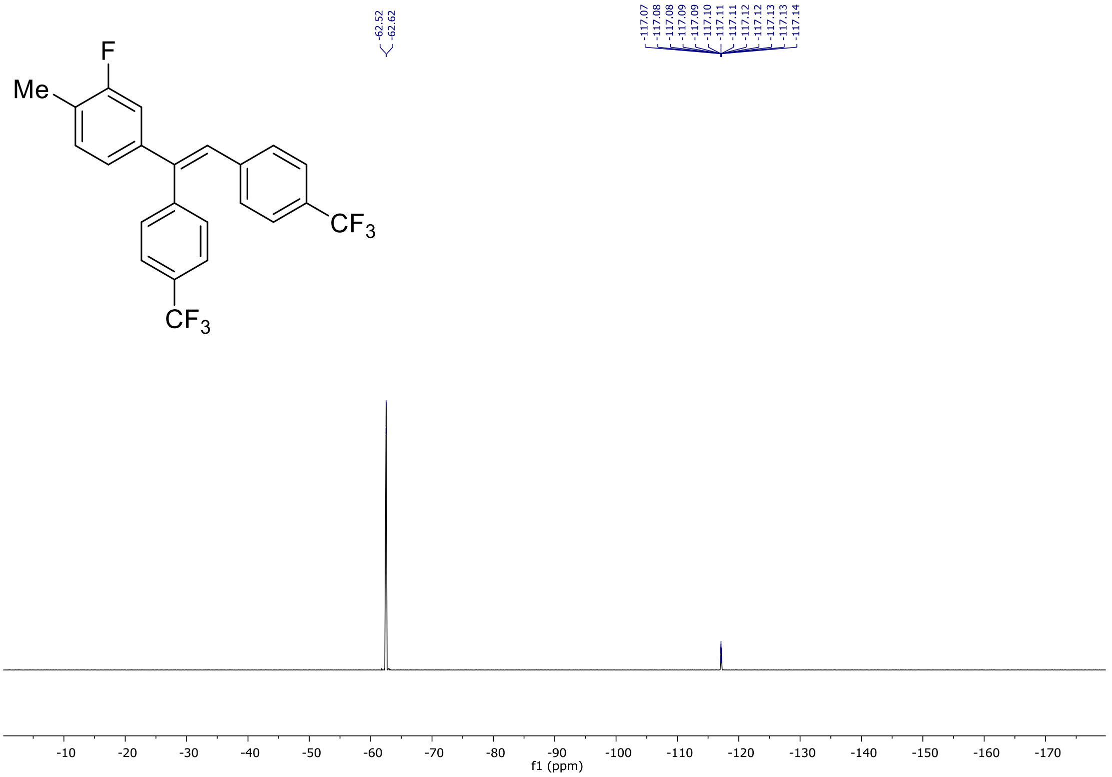

^{13}C NMR (101MHz, $CDCl_3$)

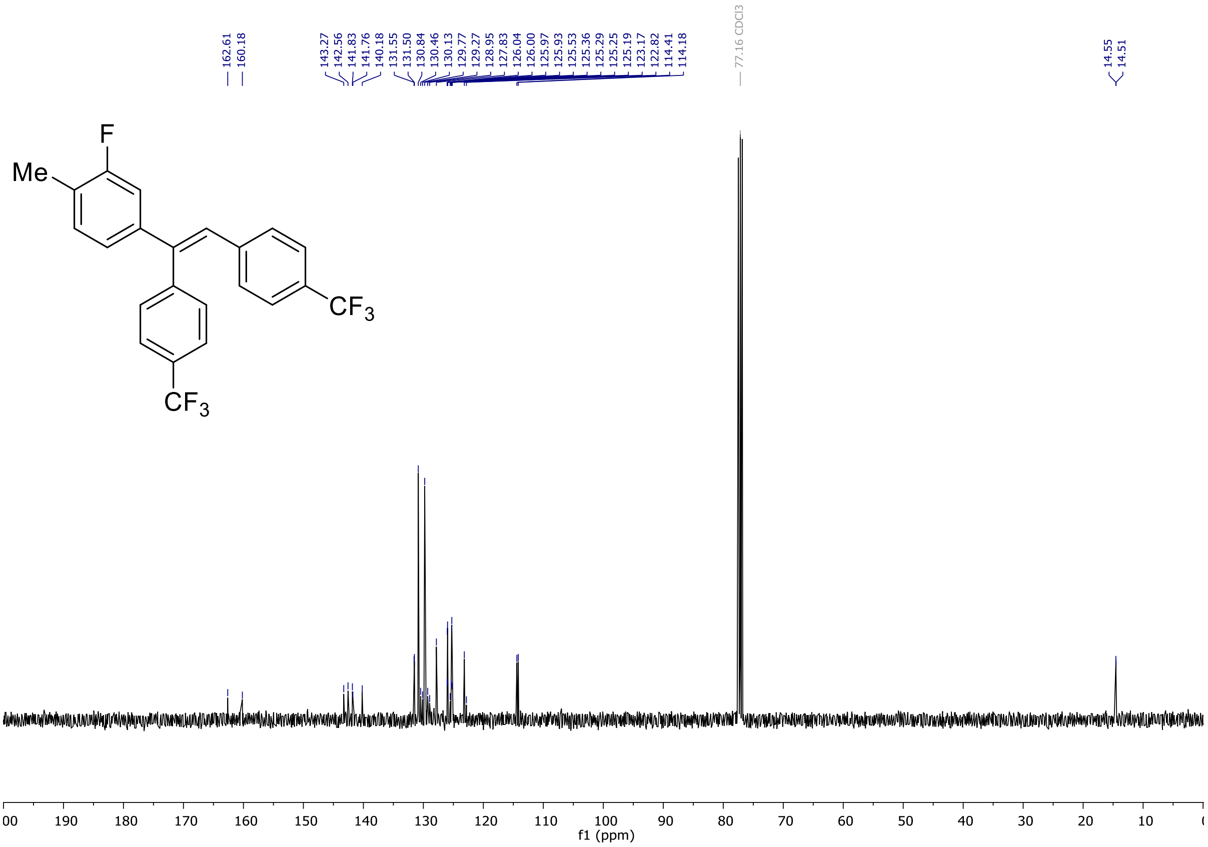

^{1}H NMR (400MHz, $CDCl_3$)

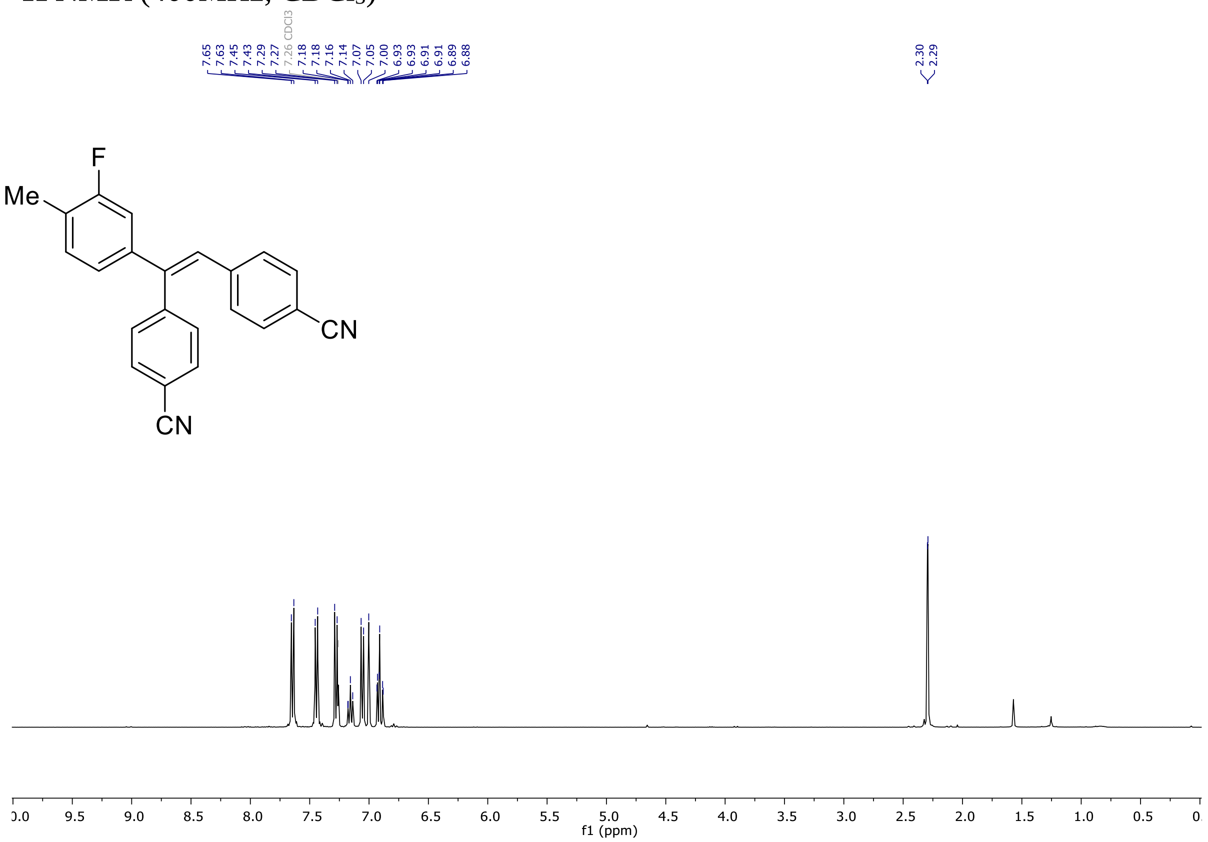

^{19}F NMR (376MHz, $CDCl_3$)

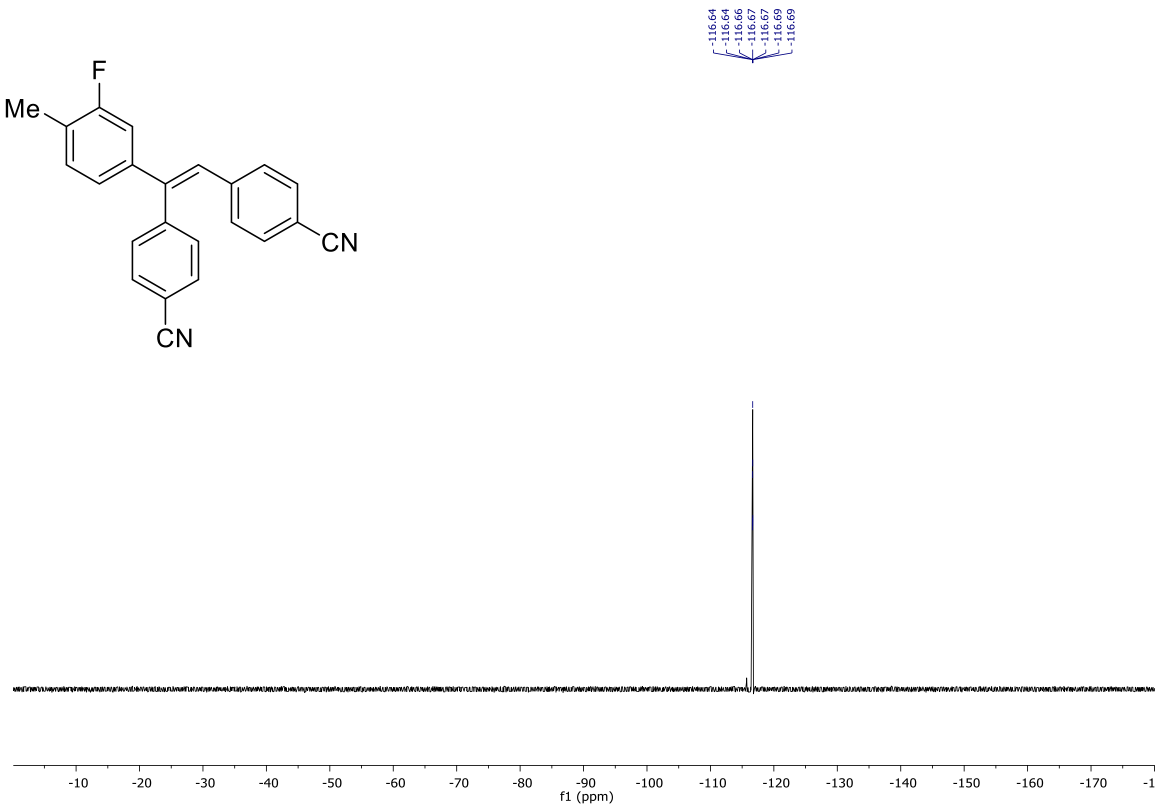

^{13}C NMR (101MHz, $CDCl_3$)

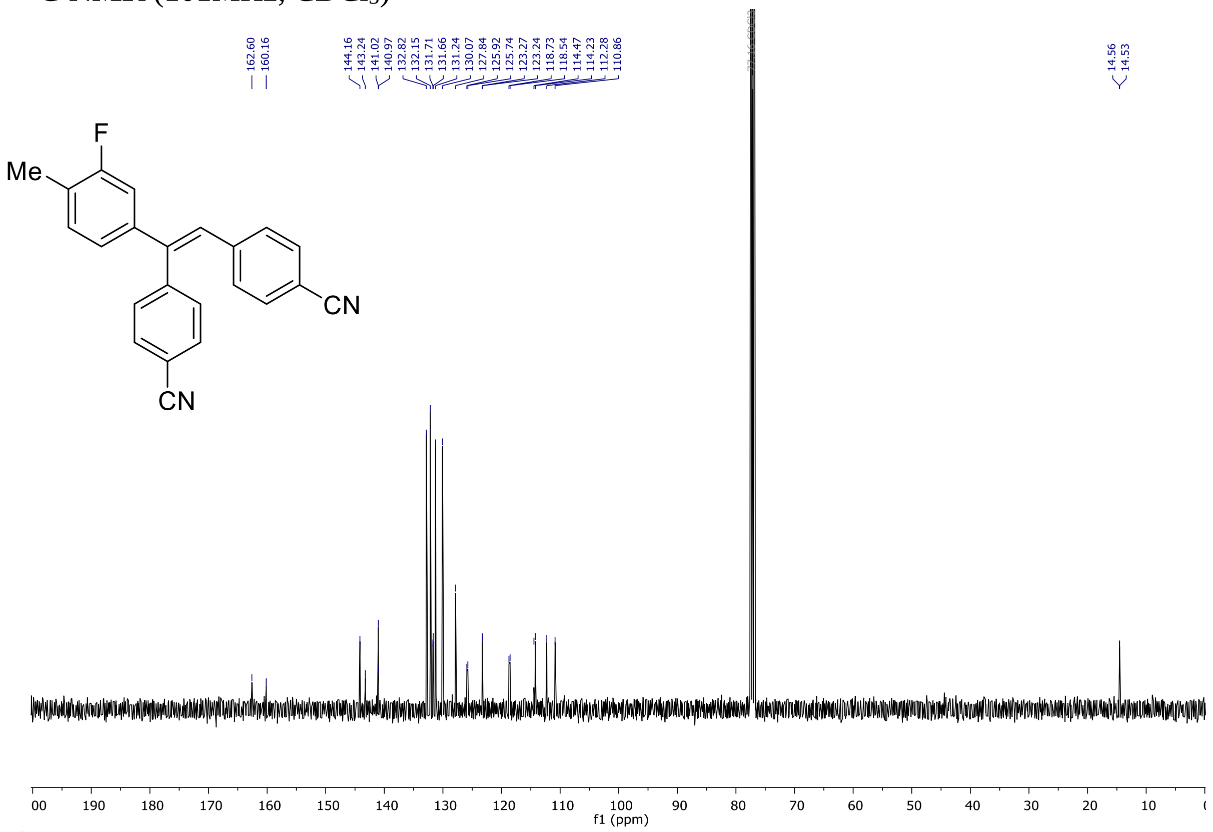

^{1}H NMR (400MHz, $CDCl_3$)

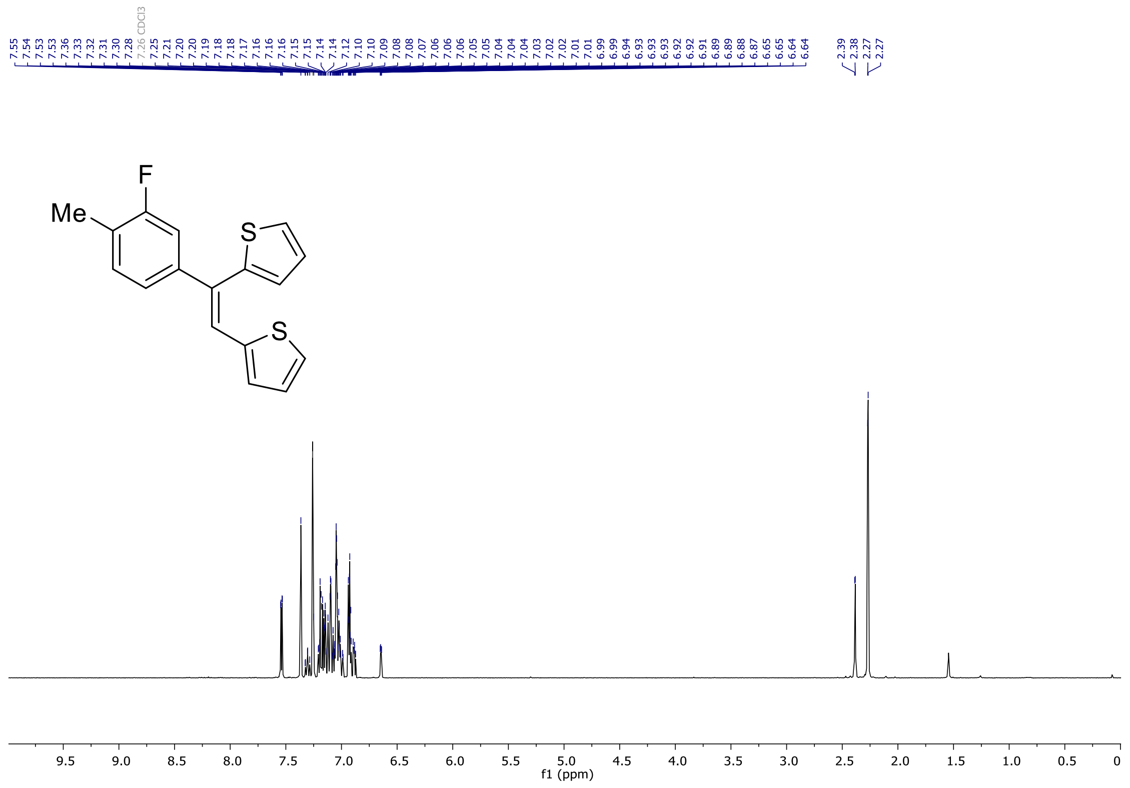

^{19}F NMR (376MHz, $CDCl_3$)

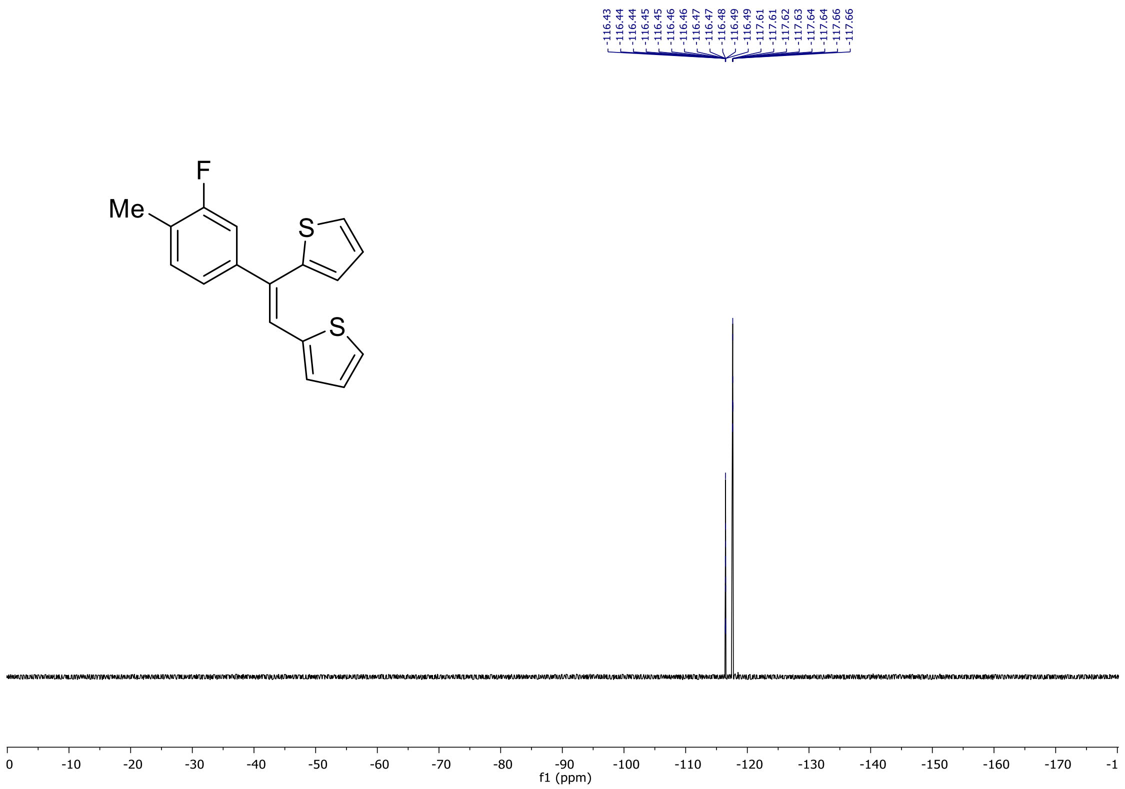

^{13}C NMR (101MHz, $CDCl_3$)

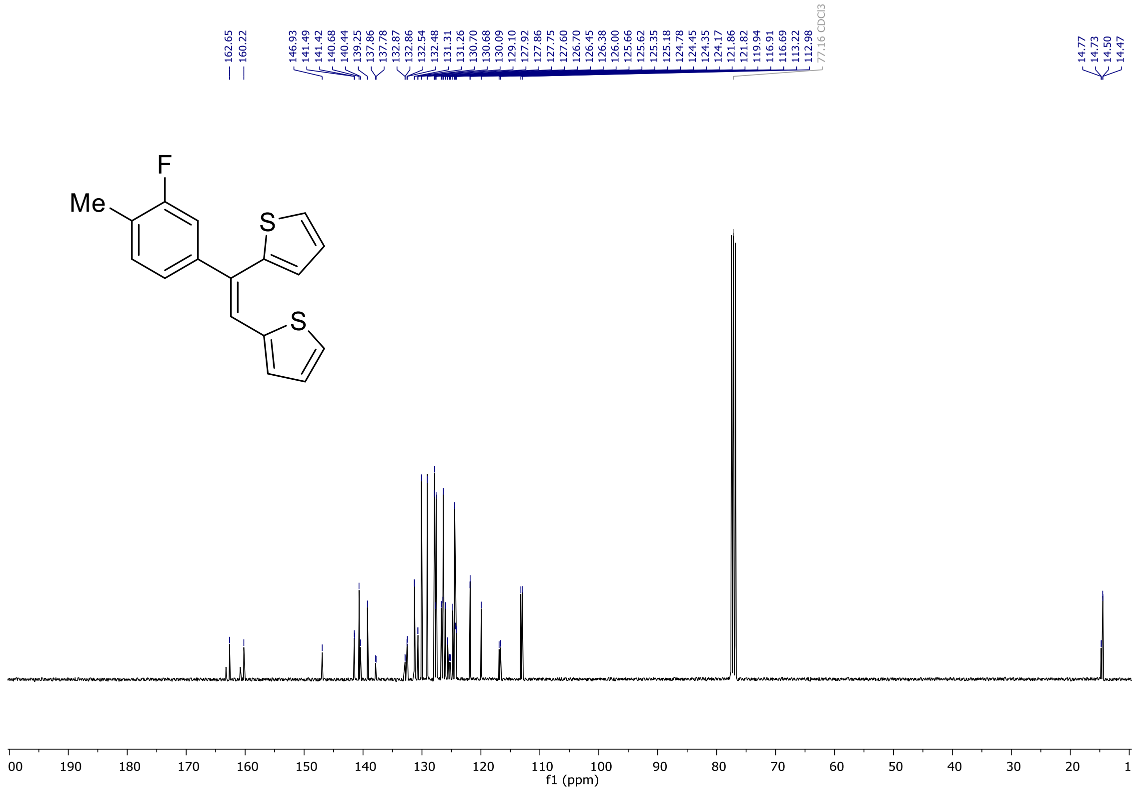

^{1}H NMR (400MHz, $CDCl_3$)

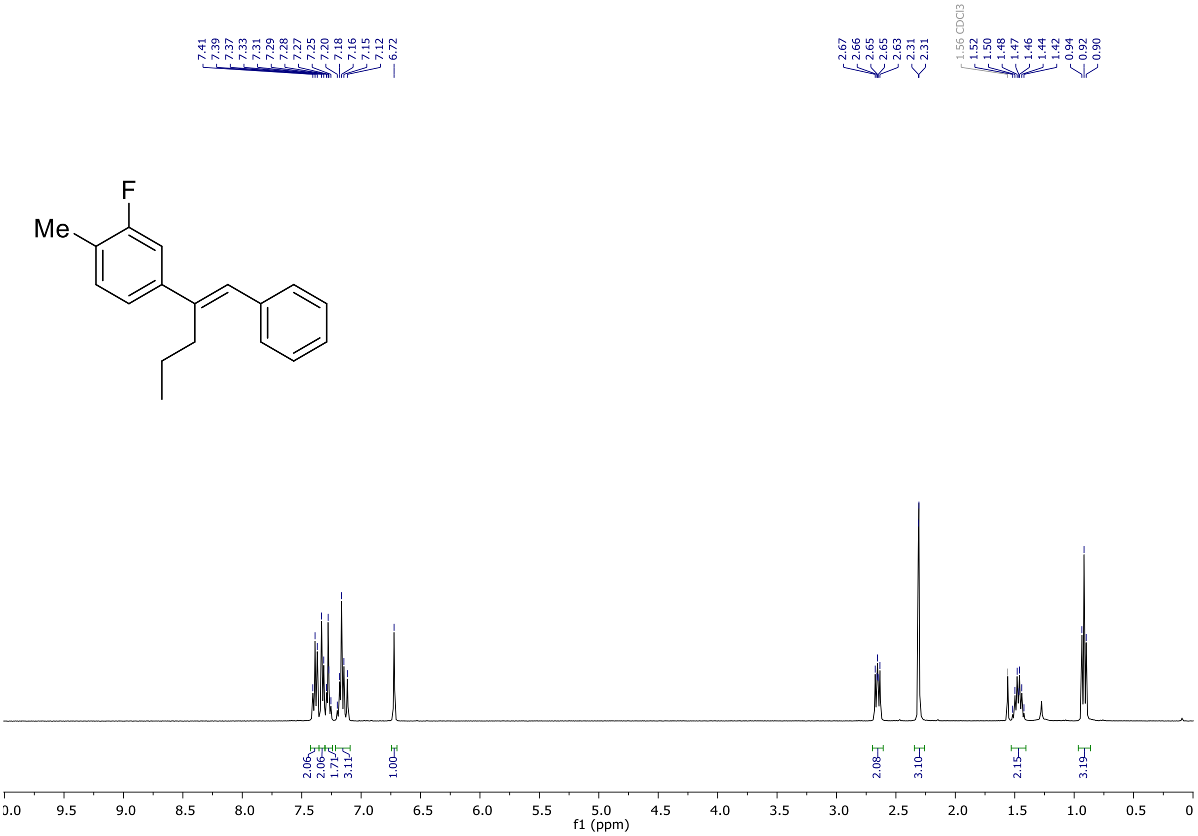

^{19}F NMR (376MHz, $CDCl_3$)

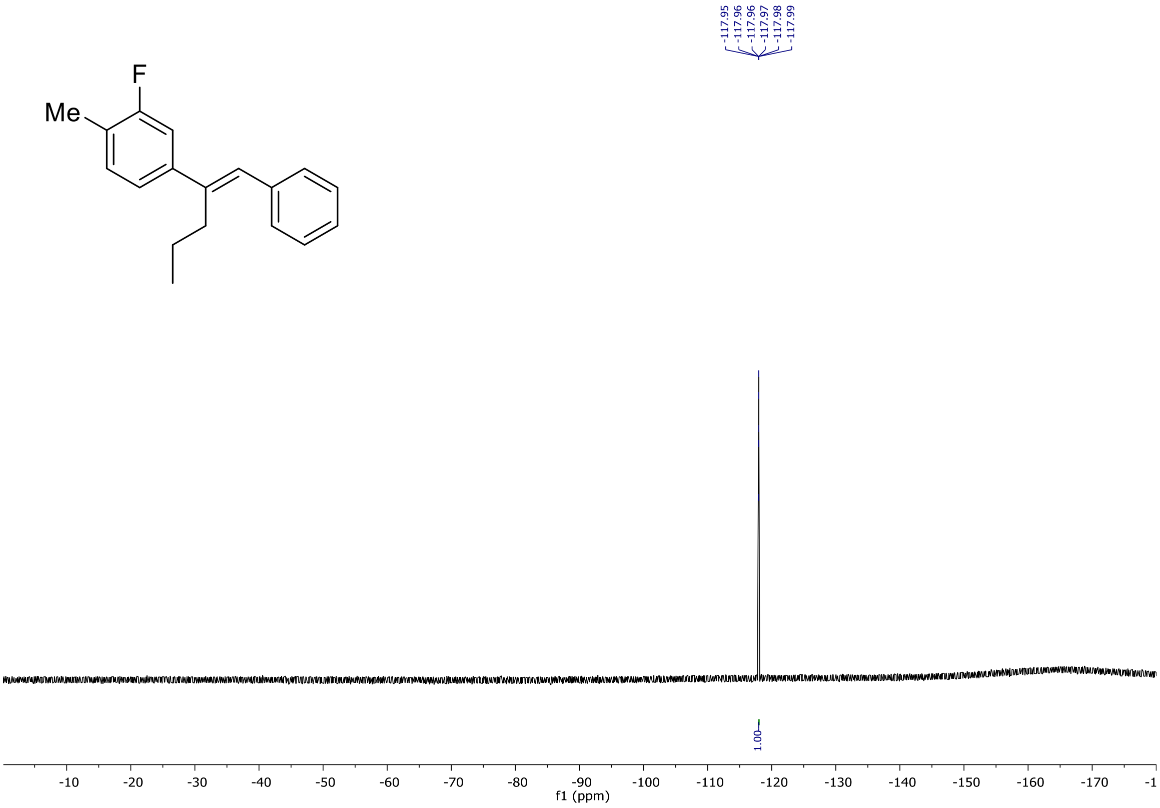

^{13}C NMR (126MHz, $CDCl_3$)

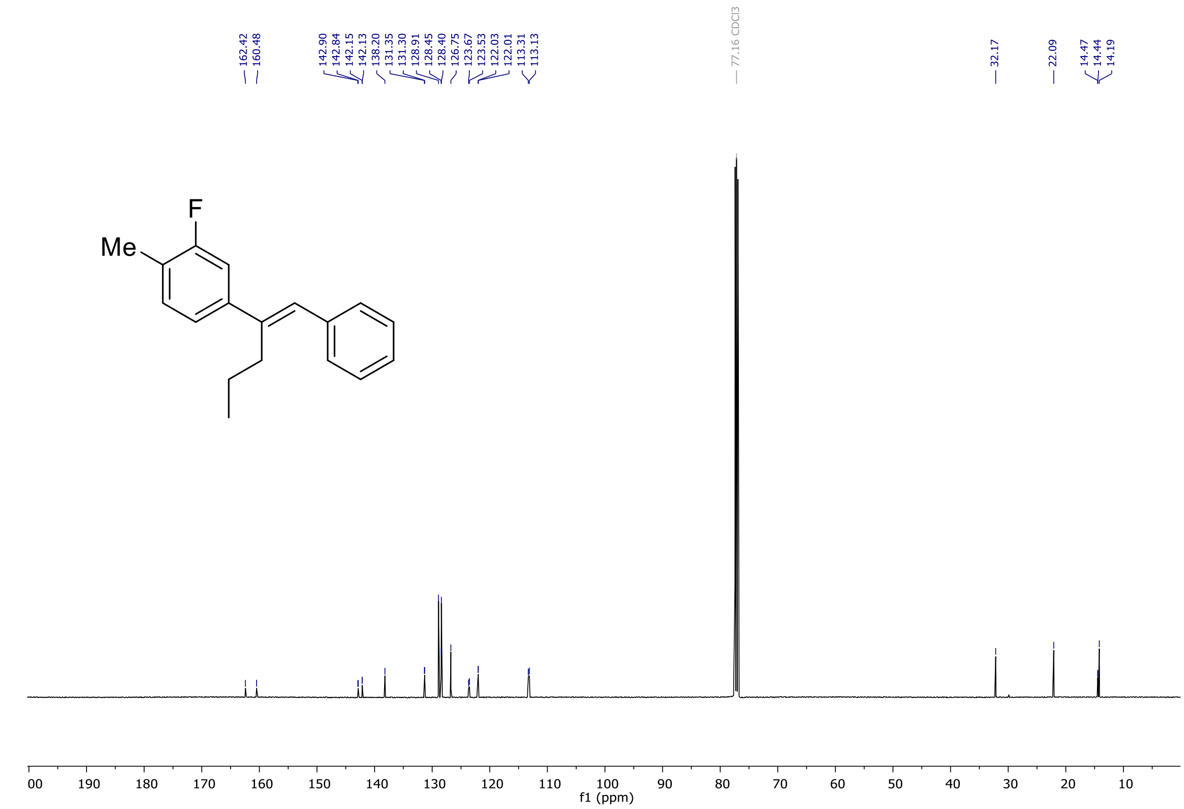

^{1}H NMR (400MHz, $CDCl_3$)

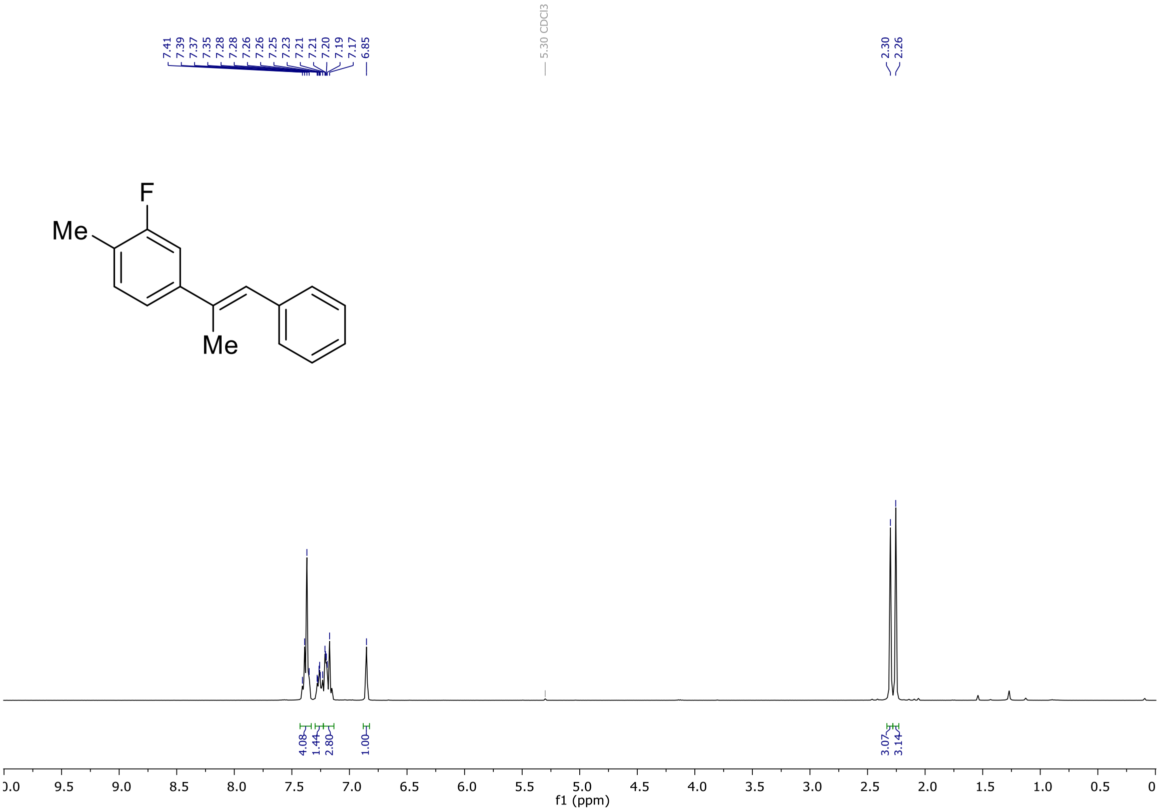

^{19}F NMR (376MHz, $CDCl_3$)

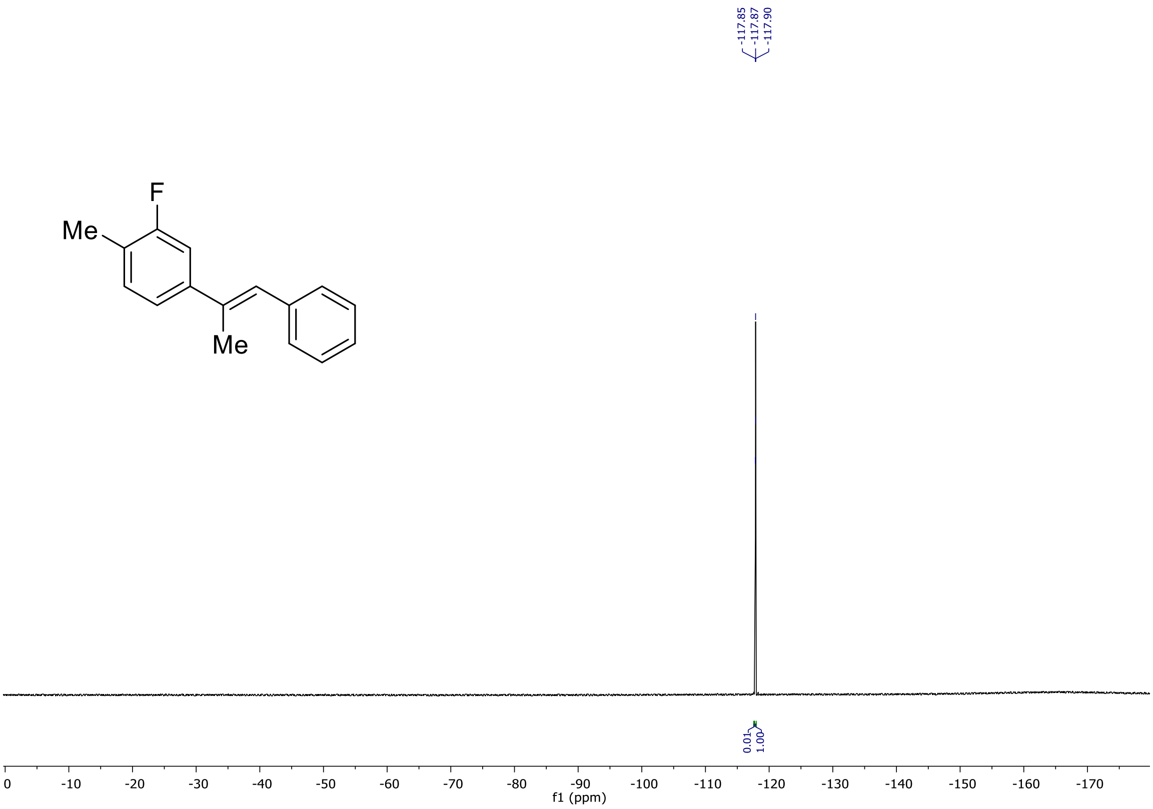

^{13}C NMR (126MHz, $CDCl_3$)

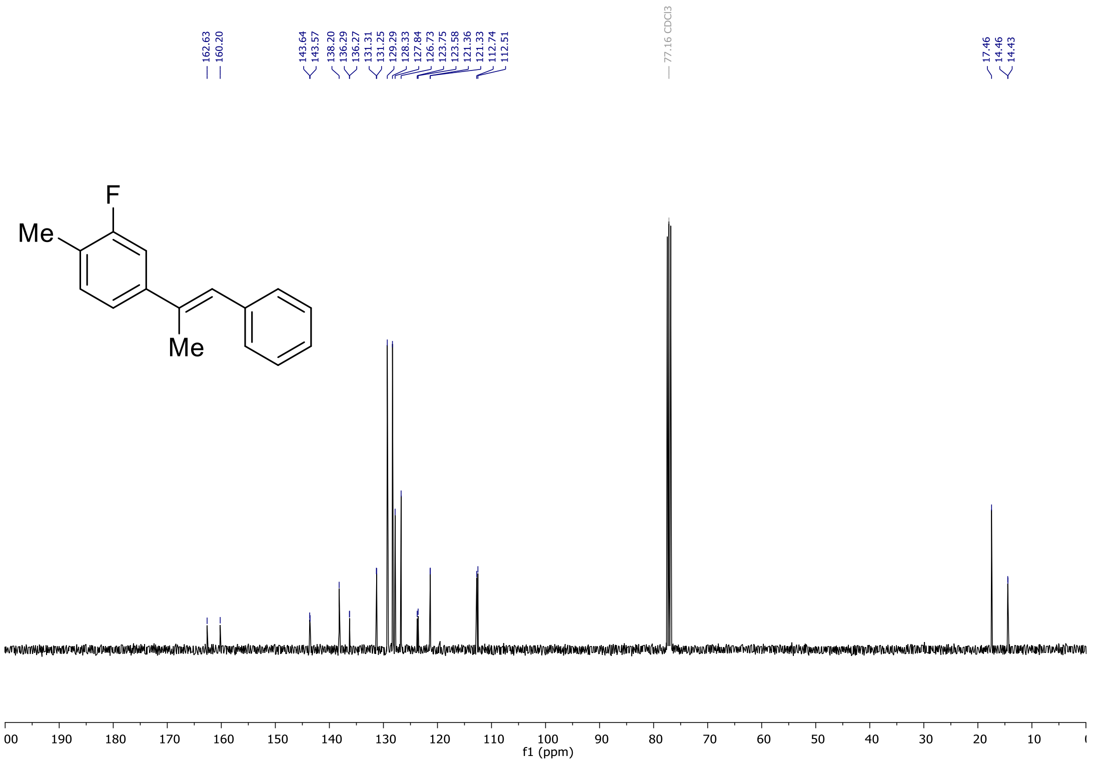

^{1}H NMR (400MHz, $CDCl_3$)

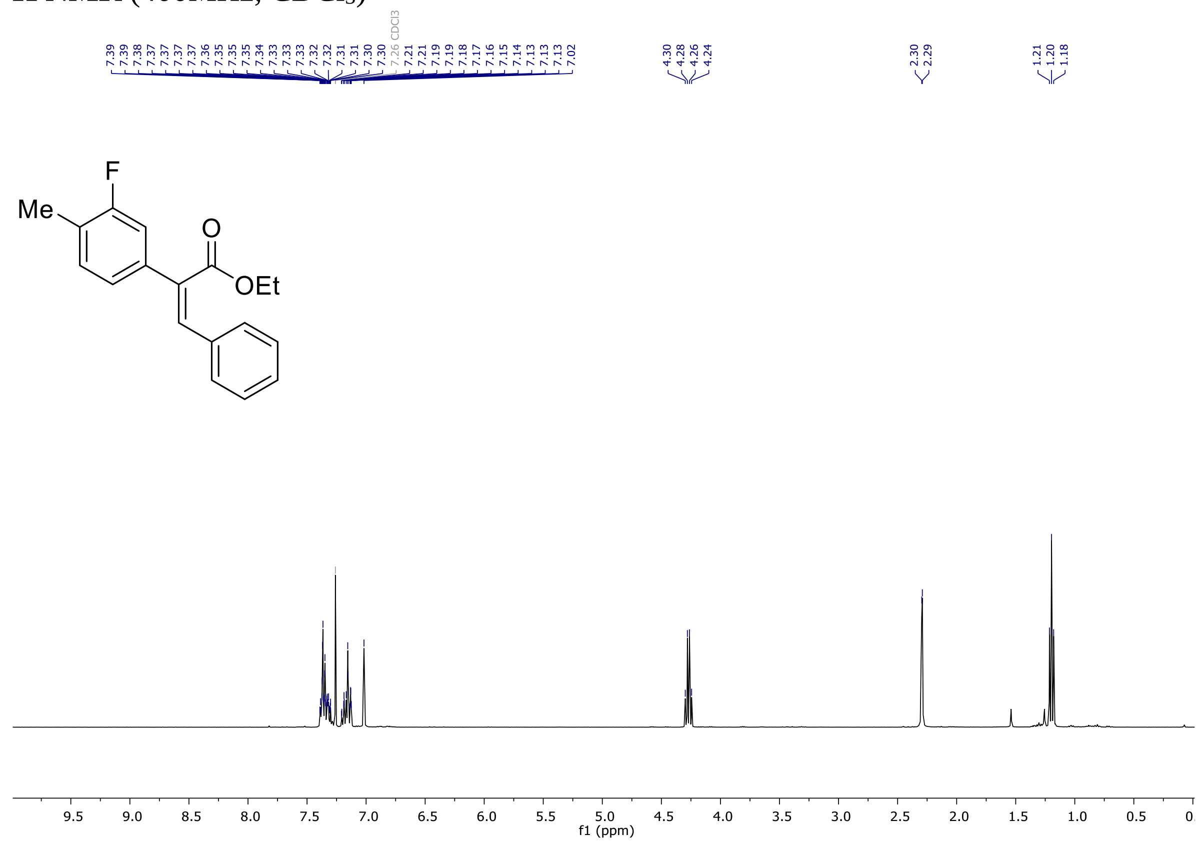

^{19}F NMR (376MHz, $CDCl_3$)

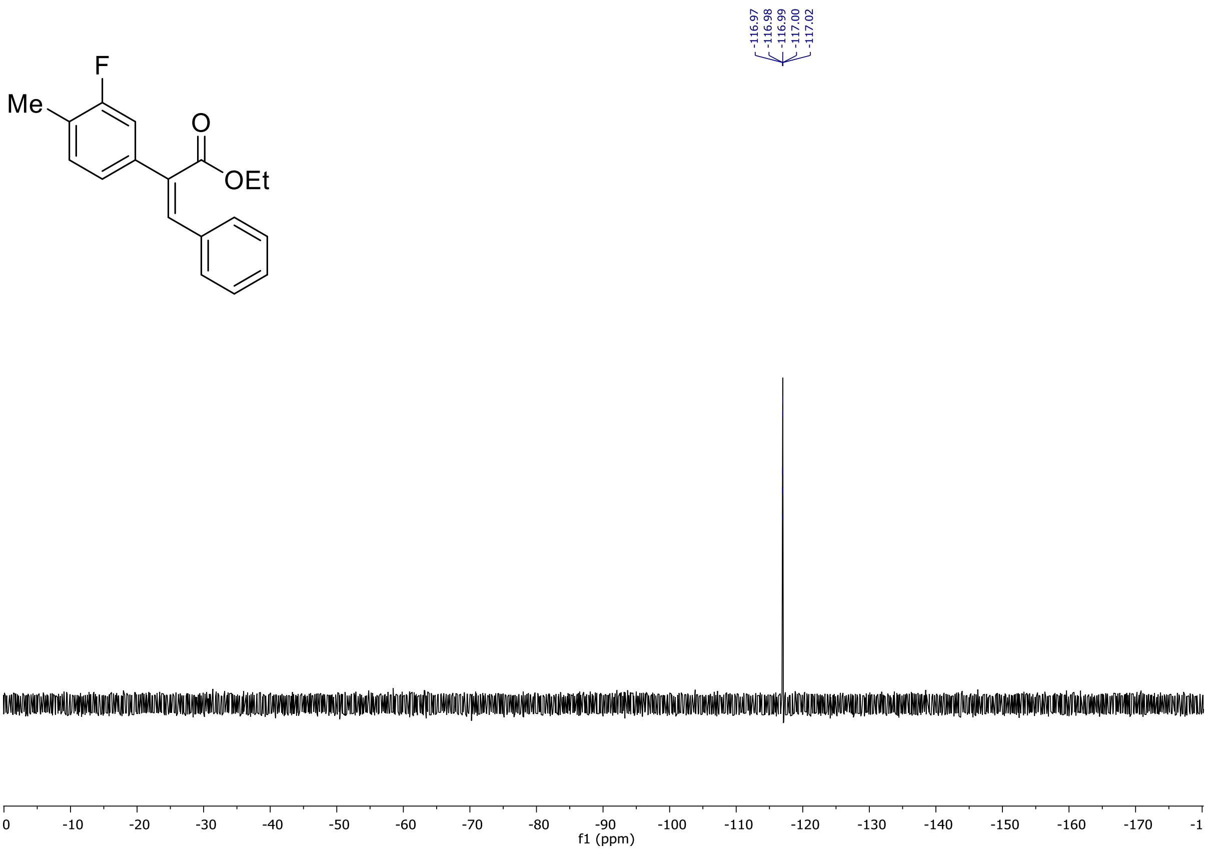

^{13}C NMR (101MHz, $CDCl_3$)

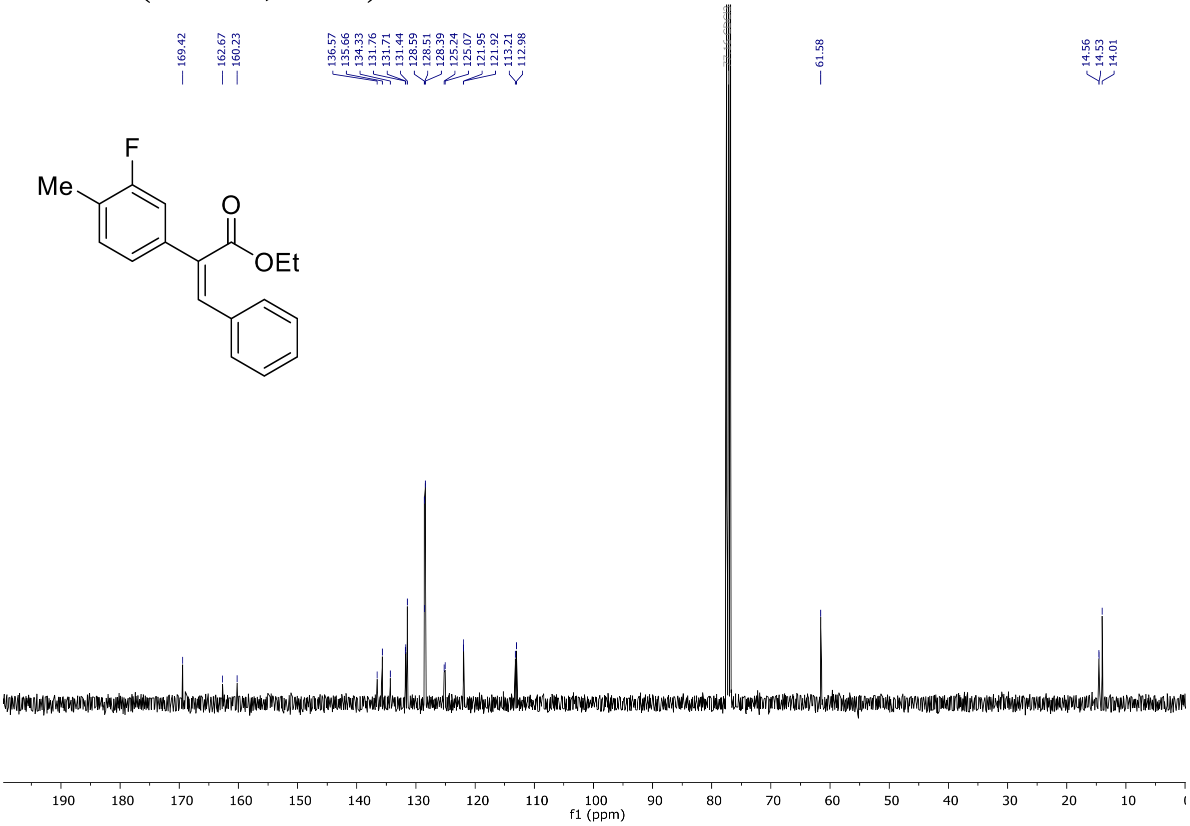

^{1}H NMR (400MHz, $CDCl_3$)

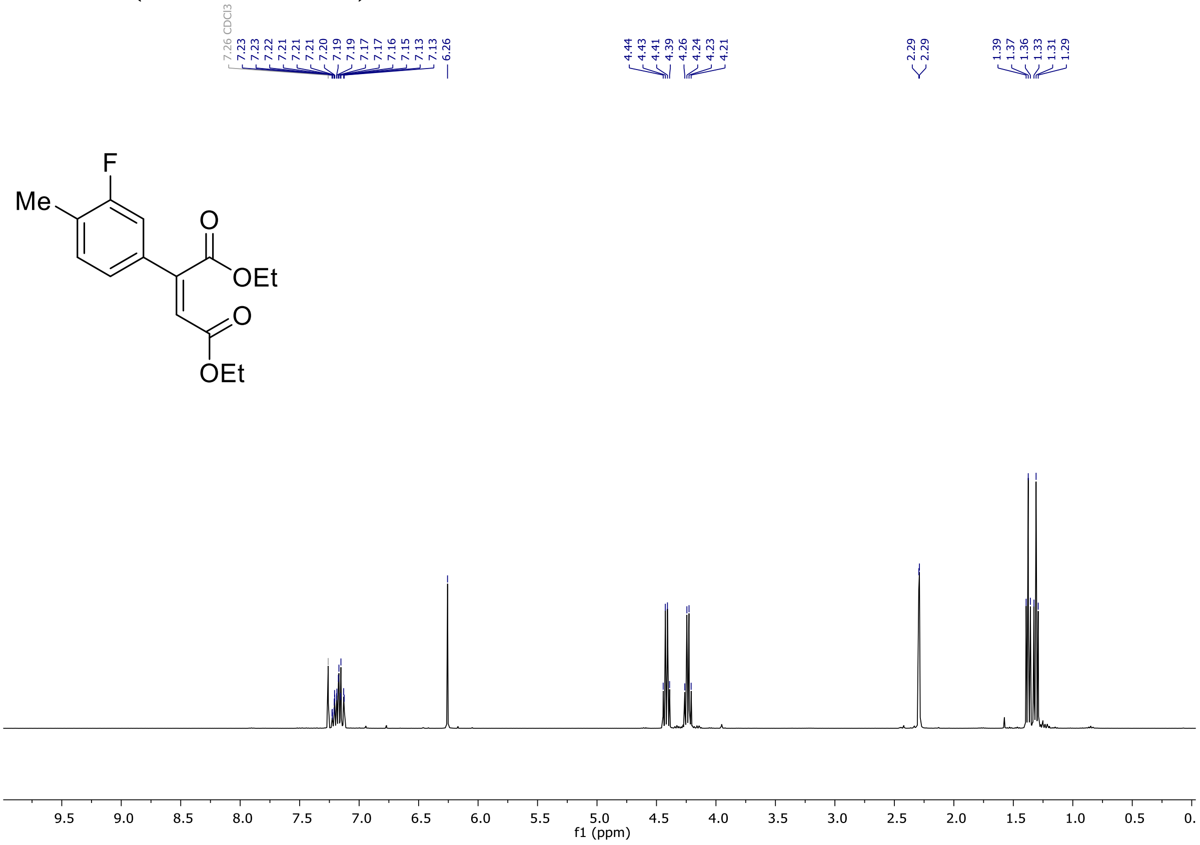

^{19}F NMR (376MHz, $CDCl_3$)

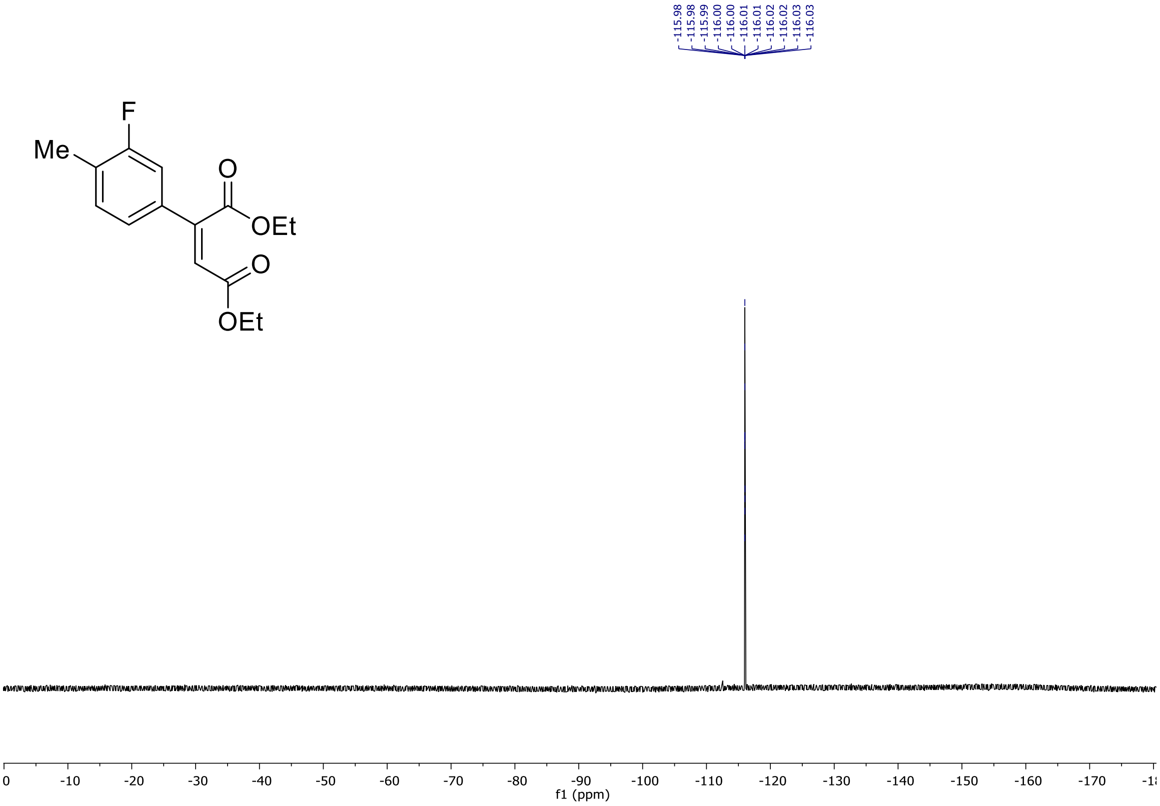

^{13}C NMR (101MHz, $CDCl_3$)

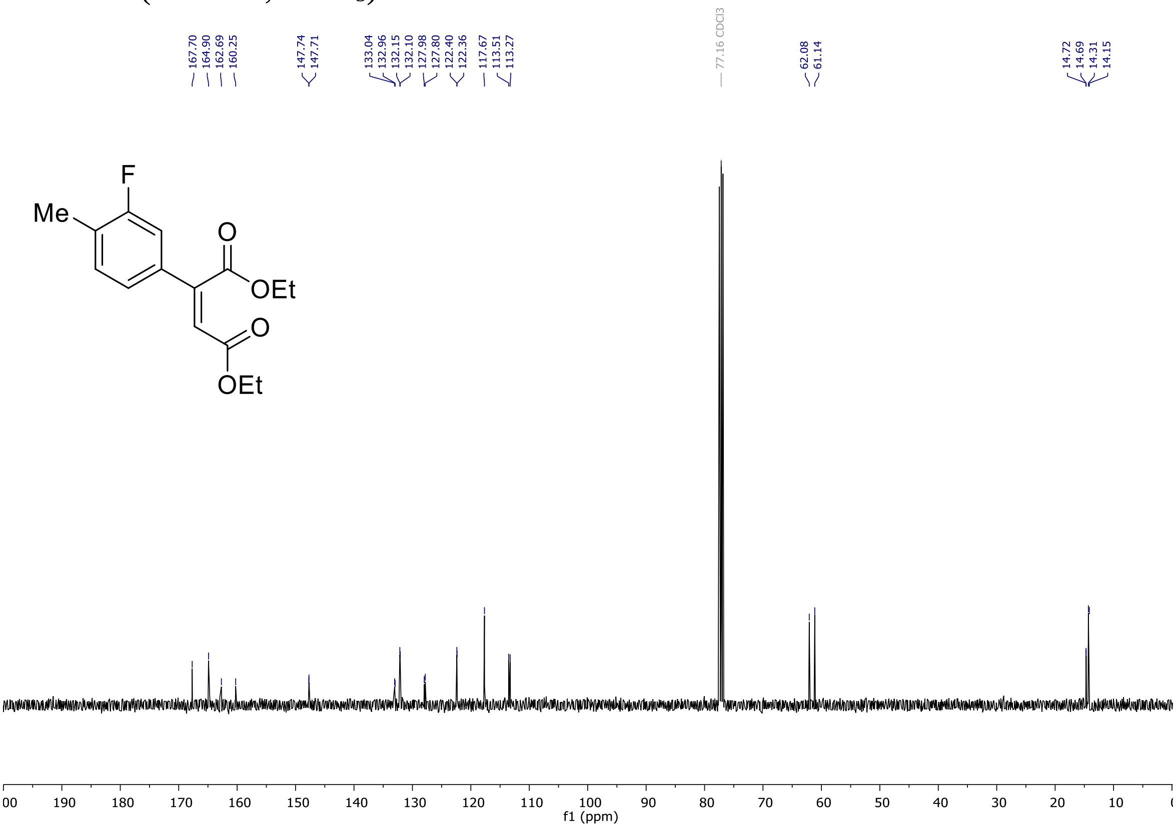

Supplement: SC-011-D0SC01138J-s001 [file SC-011-D0SC01138J-s001.pdf]
